# Delivery of Allied Health Interventions Using Telehealth Modalities: A Rapid Systematic Review of Randomized Controlled Trials

**DOI:** 10.3390/healthcare12121217

**Published:** 2024-06-18

**Authors:** Melissa J. Raymond, Lauren J. Christie, Sharon Kramer, Carla Malaguti, Zaneta Mok, Betina Gardner, Melita J. Giummarra, Serena Alves-Stein, Claire Hudson, Jill Featherston, Anne E. Holland, Natasha A. Lannin

**Affiliations:** 1 School of Translational Medicine, Monash University, Melbourne 3004, Australia; sharon.kramer@monash.edu (S.K.); melita.giummarra@monash.edu (M.J.G.); s.alvesstein@alfred.org.au (S.A.-S.); anne.holland@monash.edu (A.E.H.); natasha.lannin@monash.edu (N.A.L.); 2 Allied Health Research Unit, St Vincent’s Health Network Sydney, Darlinghurst 2000, Australia; lauren.christie@svha.org.au; 3 Nursing Research Institute, St Vincent’s Health Network Sydney, St Vincent’s Hospital Melbourne and Australian Catholic University, Darlinghurst 2010, Australia; 4 Alfred Health, Melbourne 3000, Australia; z.mok@alfred.org.au (Z.M.); c.hudson@alfred.org.au (C.H.); 5 Department of Cardiorespiratory and Skeletal Muscle, Federal University of Juiz de Fora, Juiz de Fora 36036-900, Brazil; 6 Private Practice, Melbourne 3000, Australia; betinagardner.neuro@gmail.com; 7 School of Medicine, Cardiff University, Wales CF10 2AF, UK; 8 Western Sydney Podiatry, Penrith 2750, Australia; 9 Institute for Breathing and Sleep, Melbourne 3084, Australia

**Keywords:** telerehabilitation, telehealth, rehabilitation, allied health, physiotherapy, occupational therapy, psychology, speech therapy, language, systematic review, recovery of function, patient satisfaction

## Abstract

**Objectives:** To determine whether allied health interventions delivered using telehealth provide similar or better outcomes for patients compared with traditional face-to-face delivery modes. **Study design:** A rapid systematic review using the Cochrane methodology to extract eligible randomized trials. **Eligible trials:** Trials were eligible for inclusion if they compared a comparable dose of face-to-face to telehealth interventions delivered by a neuropsychologist, occupational therapist, physiotherapist, podiatrist, psychologist, and/or speech pathologist; reported patient-level outcomes; and included adult participants. **Data sources:** MEDLINE, CENTRAL, CINAHL, and EMBASE databases were first searched from inception for systematic reviews and eligible trials were extracted from these systematic reviews. These databases were then searched for randomized clinical trials published after the date of the most recent systematic review search in each discipline (2017). The reference lists of included trials were also hand-searched to identify potentially missed trials. The risk of bias was assessed using the Cochrane Risk of Bias Tool Version 1. **Data Synthesis:** Fifty-two trials (62 reports, *n* = 4470) met the inclusion criteria. Populations included adults with musculoskeletal conditions, stroke, post-traumatic stress disorder, depression, and/or pain. Synchronous and asynchronous telehealth approaches were used with varied modalities that included telephone, videoconferencing, apps, web portals, and remote monitoring, Overall, telehealth delivered similar improvements to face-to-face interventions for knee range, Health-Related Quality of Life, pain, language function, depression, anxiety, and Post-Traumatic Stress Disorder. This meta-analysis was limited for some outcomes and disciplines such as occupational therapy and speech pathology. Telehealth was safe and similar levels of satisfaction and adherence were found across modes of delivery and disciplines compared to face-to-face interventions. **Conclusions:** Many allied health interventions are equally as effective as face-to-face when delivered via telehealth. Incorporating telehealth into models of care may afford greater access to allied health professionals, however further comparative research is still required. In particular, significant gaps exist in our understanding of the efficacy of telehealth from podiatrists, occupational therapists, speech pathologists, and neuropsychologists. **Protocol Registration Number:** PROSPERO (CRD42020203128).

## 1. Introduction

Clinicians are increasingly providing allied health treatment using telehealth, where healthcare is delivered using information and communication technologies such as telephone or videoconference [1]. Delivery of interventions via telehealth, rather than face-to-face, is acknowledged to alleviate the barriers of proximity to care, with greater access to clinicians for people with mobility restrictions, chronic health conditions, living remotely, and/or the inability to travel [2]. It may also help patients avoid taking time away from work, family, and/or other commitments, and reduce direct and indirect costs incurred by both patients and clinicians [3]. The convenience of telehealth may also increase treatment attendance rates and improve adherence to exercise interventions [4].

While telephone-based psychotherapy interventions have been delivered for decades [5,6,7], other allied health professions have been slower to adopt a telehealth mode of delivery. Key barriers include preference for face-to-face delivery reported by both patients and clinicians, technology and internet access issues, and lack of support for reimbursement by organizations and health insurance [4]. For older adults, additional challenges that impact the acceptability of telehealth can include digital literacy [2], as well as issues with fatigue, hearing, vision, and/or cognitive impairment [8,9].

Previous systematic reviews that have synthesized the evidence for allied health interventions delivered via telehealth have been condition-specific [10,11,12,13] or included trials that did not compare the efficacy of telehealth relative to traditional face-to-face interventions [14,15,16]. Consequently, it is unclear whether patient outcomes following telehealth-delivered allied health interventions are comparable to face-to-face interventions across a range of diagnoses and clinical populations. In 2020, many governments worldwide imposed restrictions on travel and non-emergency face-to-face healthcare as a response to COVID-19 [17]. For most healthcare domains, this led to a rapid transition to the use of telehealth models of care, often with little or no prior experience in this delivery modality. Evidence arising throughout that period predominantly failed to compare outcomes with face-to-face interventions, so gaps in the synthesis of evidence remain.

In response to these identified issues, the aim of this rapid review was to evaluate the efficacy of delivering allied health interventions using telehealth modalities for any condition. Comparisons between telehealth delivery and face-to-face delivery were specifically examined to assist clinician decision-making regarding the most effective mode of delivery. The specific research questions were as follows:

1. How effective are allied health interventions delivered using telehealth modalities compared with interventions delivered face-to-face of a comparable dose?
  - What are the telehealth modalities and interventions used by allied health clinicians?
  - What outcomes are influenced (impairment, activity limitation, participation)?
  - Are there differences in adherence and safety between telehealth and face-to-face delivery?

## 2. Method

The review protocol was registered on PROSPERO (CRD42020203128) prior to commencement. The review methodology met the criteria for abbreviated systematic review methods as described in the Cochrane Rapid Review guidelines [18].

### 2.1. Identification and Selection of Trials

A three-phase search strategy was undertaken. First, CENTRAL, CINAHL, EMBASE, and MEDLINE were searched to identify existing systematic reviews from inception to August 2020, and from these, the included RCTs were screened for inclusion in this rapid review using the pre-defined eligibility criteria (Box 1). Second, these same databases were searched for RCTs from the year of the latest systematic reviews in each discipline (2017) to October 2023. The third step was to hand-search reference lists of included trials for published RCTs, cluster-RCTs, or quasi-RCTs published in English that met the pre-defined eligibility criteria. The searches were conducted by one investigator (MJR) and checked by a second investigator (NAL) (Supplementary Table S1).

To expedite this rapid review, a computer-assisted screening of citations was undertaken with Abstrackr (beta version; Rhode Island, USA) [19] to reduce the workload on dual-screening citations through machine learning by predicting the relevancy of citations [20]. All citations from phase one were loaded into Abstrackr and screened once (MJR, LJC, CM) with dual screening until no remaining records were predicted to be relevant. Text mining was then used to reduce the assessment of full-text publications for potential eligibility. All PDFs were loaded into WordStat (MJG) (Version 7.1.21; Provalis, Montreal, QC, Canada) via QDA Miner (Version 5.0.21; Provalis, Montreal, QC, Canada), and a dictionary of terms for allied health disciplines was used to identify reviews of the relevant literature that mentioned any allied health discipline in the review methods or results sections (MJG, MJR) (Supplementary Table S2). Manual eligibility screening was then limited to systematic review full-text articles that included one or more allied health terms, telehealth terms, and RCT terms. Full texts were reviewed by one reviewer (MJR, LJC, SK, CM, JF, BG, SA, CH, JF, SK, ZM, AL). A second reviewer (MJR) cross-checked 20% of all excluded records for accuracy. Discrepancies were resolved by consensus. Reasons for exclusion were recorded for all trials that were excluded after a full-text review (Supplementary Table S3).

Box 1 **Inclusion criteria.**

Design:
Systematic reviews (phase 1), randomized (individual patient or clustered), or quasi-randomized controlled trials (phase 2 and phase 3)
Participants:
Adults requiring allied health intervention
Intervention: allied health intervention delivered via telehealth At least one allied health professional (physiotherapy, occupational therapy, speech pathology, psychology, neuropsychology, or podiatry) providing an intervention
The intervention could also include allied health professionals other than those specified aboveThe intervention could include a mix of telehealth and face-to-face with or without additional technology, such as virtual reality, smartphone applications, or personal computer/tablet programsLocation may include the participant’s home (community), or another hospital (not co-located)
Comparison: face-to-face allied health intervention Delivered by at least one allied health professional: physiotherapist, occupational therapist, speech pathologist, psychologist, neuropsychologist, and/or podiatrist
Comparable dose and type of intervention were provided to the intervention and comparator groups (e.g., one hour of weekly face-to-face strength training versus telehealth strength training)
Outcome measures:
Outcome measures reported at pre-treatment and treatment completion with or without follow-up for up to 12 months

Patient level outcomes were measured against the International Classification of Functioning Codes (World Health Organization, 2009) relevant to neuropsychology, occupational therapy, physiotherapy, podiatry, psychology, and speech pathologyThe primary outcome was independence assessed post-intervention, encompassing self-care, mobility, and/or participation domains. Any assessment tools were includedSecondary outcomes included self-care, mobility (e.g., walking speed, functional ambulation category), balance, upper extremity function, language and communication, cognition, and depression (global measures)Program duration, participant satisfaction with the intervention; self-reported Health-Related Quality of Life; and adverse events (including falls, hospital use)Feasibility of telehealth
Exclusion criteria:
Trials where more than 20% of the participants were aged younger than 18 years, or the sample was receiving obstetric or peri-natal treatment, or interventions addressing drug, tobacco, or alcohol use (to limit the scope of rapid review)Trials comparing telehealth methods without a face-to-face intervention comparison group

Trials published in any language other than English as the translation was not availableTrials comparing two different types of intervention (e.g., education face-to-face versus active therapy via telehealth)

Trials evaluating allied health assessment or monitoring only with no interventionTrials where the allied health professional providing the telehealth intervention was co-locatedOnline-only/computer-based programs without the involvement of allied health professionals (e.g., computer-based independent exercise program)Interventions that include medical, nursing, or non-listed allied health/pharmacy professionals where the effects of the intervention component delivered by the allied health professional cannot be isolated from the interventions provided by the other professional group/sInterventions delivered by an allied health or rehabilitation assistant without clear supervision or delegation from an allied health professional

### 2.2. Assessment of Quality of Trials

The quality of the included trials was assessed using the Cochrane Risk of Bias Tool Version 1. All trials were reviewed by one reviewer (MJR) and a second reviewer (LJC) cross-checked a random sample of 30% of the studies and discrepancies were resolved by discussion.

### 2.3. Data Extraction and Analysis

Data were extracted from RCTs by one reviewer with clinical expertise in the respective allied health discipline (LJC, CM, BG, ZM, CH, SA, AL) and a second reviewer (LJC, CH, MJR, NAL) checked 100% of the data for completeness and accuracy. Discrepancies were resolved by discussion between reviewers. Data were extracted using pilot-tested data extraction forms by one reviewer (MJR) and verified by a second author (LJC) using an Excel spreadsheet. Data extracted included study author, publication year, study design (including methods, and geographic location), setting, participant characteristics, intervention characteristics (including clinicians, frequency, duration, intensity), comparator characteristics and study effects on outcomes of interest, adverse events, satisfaction, and adherence.

Outcome data were extracted for pre-treatment, treatment completion, and, where available, follow-up periods for up to 12 months post-treatment. Data were extracted according to the International Classification of Functioning criteria [21]. Outcomes were reported post-intervention as well as at secondary time points (three months or longer). Continuous outcomes were reported as mean differences (MDs) and standardized mean differences (SMDs) with 95% Confidence Intervals. To account for between-study heterogeneity, random-effects meta-analyses were performed where possible, where the post-intervention scores were used to obtain the pooled estimate of the effect of telehealth intervention compared with face-to-face intervention using RevMan 5.4 software (Cochrane Collaboration, Oxford, UK). To interpret the treatment effects, the guidelines suggested by Cohen were applied for interpreting the magnitude of the SMD in the social sciences: small SMD = 0.20; medium SMD = 0.50; and large SMD = 0.80 [22]. Dichotomous outcomes were reported as a risk ratio (RR) and 95% Confidence Intervals (CI). Given that the RR describes the multiplication of the risk that occurs with the use of telehealth interventions, we interpreted an RR of 1.00 and 95%CI that included 1.00 to mean that the estimated effects are the same for both interventions (telehealth or face-to-face intervention). Heterogeneity was assessed using a Chi-Square test and I^2^ statistic. Sensitivity analyses were planned by comparing results with and without quasi-randomized trials and trials with an unclear or high risk of bias for allocation concealment. Estimations of the sample mean and standard deviation for inclusion in meta-analyses were calculated using the methods outlined by Wan et al. [23]. Missing summary statistics for within-study means were calculated using the formula for combining groups outlined in the Cochrane Handbook [18] and missing mean differences were calculated using RevMan 5.4 software (Cochrane Collaboration, Oxford, UK). Forest plots were generated for sufficiently homogenous trials within disciplines, with similar patient cohorts, interventions, and outcomes. Where a meta-analysis was not possible, intervention effects were reported as a narrative synthesis. There were a range of populations included in the trials and, as the telehealth model may potentially be more beneficial for certain disciplines, each discipline was analyzed separately.

## 3. Results

### 3.1. The Flow of Trials through the Review

**Figure 1:**
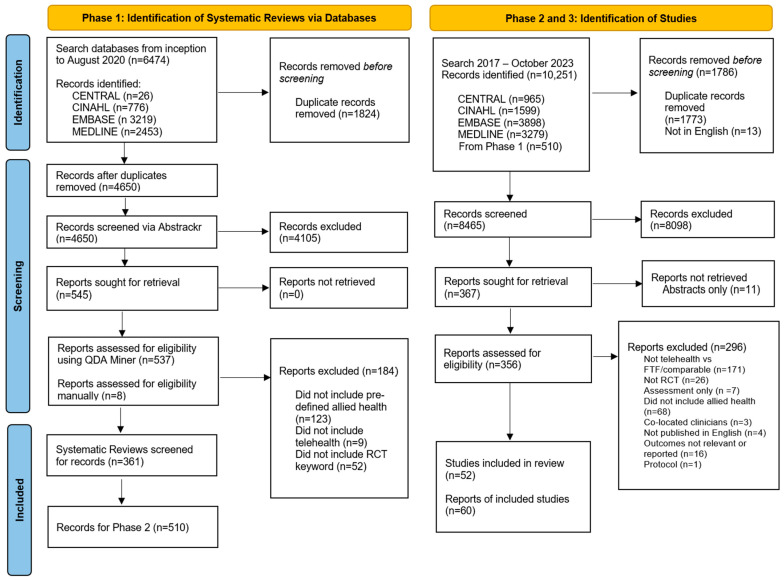
PRISMA flow chart of studies [24].

The search for systematic reviews yielded 6474 titles. From this search, 545 potentially relevant reviews that compared telehealth with face-to-face intervention were identified. From these two phases, a total of 8465 titles and abstracts were screened (see Figure 1) and 60 publications (52 trials) were identified to have met the inclusion criteria.

### 3.2. Characteristics of Included Trials

A total of 4395 participants were included in the 52 included trials (Table 1). Sample sizes ranged from 17 [25,26,27] to 325 [28] participants and interventions ranged from 2 weeks [29,30] to 6 months [31]. Neuropsychologists provided intervention in one trial [26], occupational therapists in six trials [25,30,32,33,34,35], physiotherapists in twenty-six trials (30 reports) [27,29,30,32,34,36,37,38,39,40,41,42,43,44,45,46,47,48,49,50,51,52,53,54,55,56,57,58,59,60], psychologists in seventeen trials (19 reports) [28,31,61,62,63,64,65,66,67,68,69,70,71,72,73,74,75,76,77], and speech pathologists in five trials (7 reports) [78,79,80,81,82,83,84]. We did not identify trials comparing the delivery of podiatry interventions by telehealth with a face-to-face intervention. Nine trials included intervention by health professionals from more than one discipline [30,32,34,39,46,48,49,67,70].

Not all telehealth interventions were delivered independently beyond the initial orientation period; five trials provided in-person support (from an assistant, volunteer, or technical support) with participants to assist with the use of technology and/or interventions [26,27,34], or at the remote clinic for technological troubleshooting or clinical emergencies (clinician or research coordinator) [73,74]. Whilst offering a comparable dose of intervention, some trials also included a mix of face-to-face sessions alongside telehealth; three included initial face-to-face session(s) [33,39,49], four trials included one to three face-to-face sessions for some or all trial participants [38,41,59,68] and the remainder offered weekly or bi-weekly face-to-face interventions [42,51].

### 3.3. Technologies Used

Telehealth communication was primarily delivered via telephone [28,29,31,36,40,41,44,47,49,55,56,60,62,77,78], videoconferencing (VC) [26,27,30,32,33,34,35,37,38,39,43,46,48,52,53,54,57,58,59,61,63,64,66,67,68,69,70,71,73,74,75,76,79,80,81,82,83,84], and remote monitoring and progression by a therapist via a website portal/app [38,41,42]. Additional methods to support participants remotely included virtual reality or input devices [32,51], fax [71], audiotapes [78], pre-recorded slides [48], videos, websites, apps [27,38,41,42,44,49,54,57], CDs/DVDs [77], logbooks, workbooks or diaries [25,28,39,40,43,47,55,56,60,62,77,84], and devices to monitor for range, movement, and/or vital signs [27,29,39,42,46,47,49]. Asynchronous methods, where participants exercised independently and received support and feedback from their allied health professional via telehealth, were also utilized [29,37,38,40,41,42,47,49,55,56,59,60]. The equipment for VC included participant-owned technology or equipment provided specifically for the trial. Further trial details are reported in Table 1.

### 3.4. Risk of Bias

**Figure 2:**
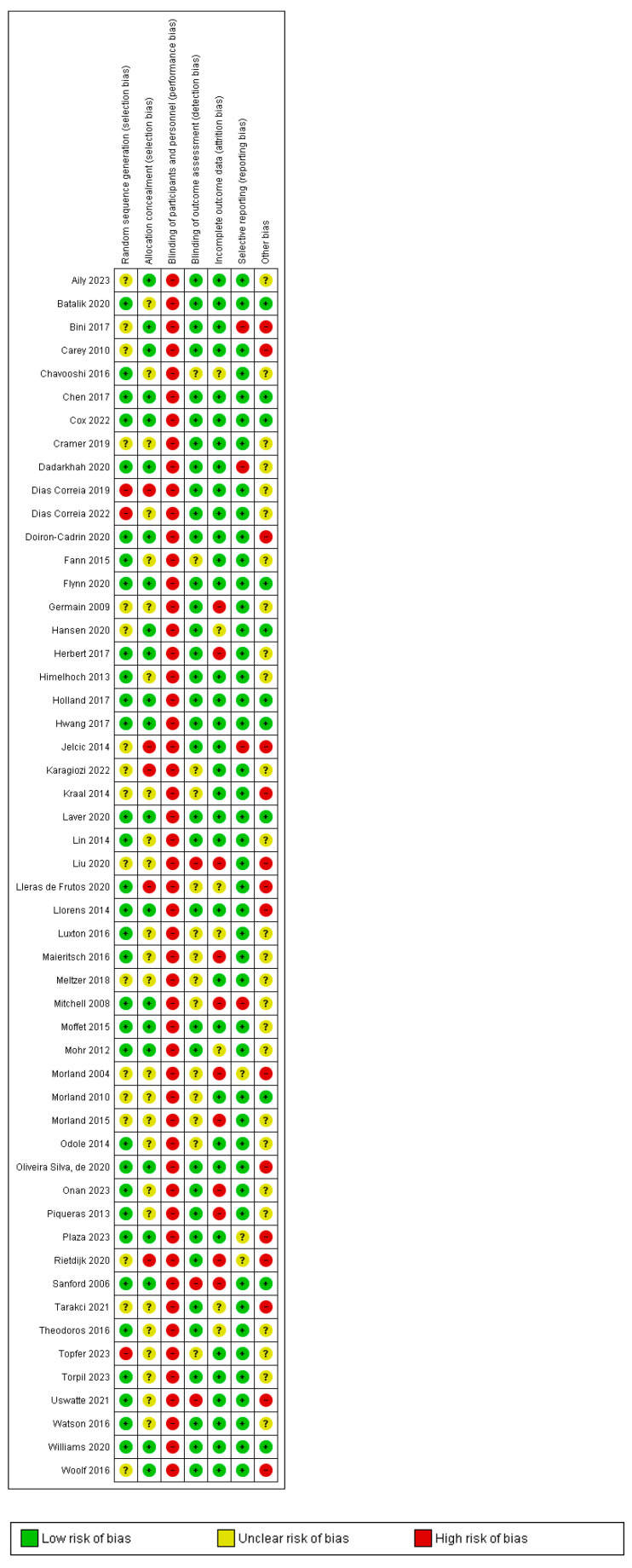
Risk of bias of included studies [25,26,27,28,29,30,31,32,33,34,35,36,37,38,39,40,41,42,43,44,45,46,47,48,49,50,51,52,53,54,55,56,57,58,59,60,61,62,63,64,65,66,67,68,69,70,71,72,73,74,75,76,77,78,79,80,81,82,83,84].

Random sequence generation was reported in 32 trials. Four trials utilized location as a means of randomization [31,41,71,80,84]. Eighteen trials were at a high risk of bias due to incomplete data. Full details are summarized in Figure 2.

### 3.5. Design and Effects of Neuropsychology Interventions

One small trial (*n* = 17) included a neuropsychological intervention providing group cognitive rehabilitation for adults with early-stage Alzheimer’s Disease [26]. Participants received lexical-semantic stimulation either face-to-face or via VC. Both modes of delivery significantly improved global cognitive performance measured by the Mini-Mental State Examination although there were no between-group differences: MD = 1.20, 95%CI: −0.73, 3.13, *p* = 0.22. Only the telehealth group improved semantic fluency (MD = −7.10, 95%CI: −13.31, −0.89, *p* = 0.03) and improvements favored telehealth for phonemic fluency (MD = −8.50, 95%CI−17.46, 0.46, *p* = 0.06). Only face-to-face intervention improved measures of immediate episodic memory (immediate recall: MD = −8.80, 95%CI: −16.61, −0.99, *p* = 0.03). There were no between-group differences for working memory, visual-spatial memory, or attention.

### 3.6. Design and Effects of Occupational Therapy Interventions

All six trials compared home or center-based face-to-face intervention with home-based synchronous telehealth intervention via VC [25,30,32,33,34,35]. Three trials also included physiotherapists, which are reported in this section [30,32,34]. There was a wide variety of interventions which included strength training, functional tasks, and/or a range of motion exercises [25,32,34], Constraint-Induced Movement Therapy (CIMT) [30], electromyography-triggered neuromuscular stimulation [25], cognitive rehabilitation [35], and a carer program [33].

#### 3.6.1. Cognition

In older adults with amnestic mild cognitive impairment, Torpil et al. [35] reported larger improvements were demonstrated with center-based face-to-face cognitive rehabilitation over VC in three of the eight subscales: visual perception (*p* < 0.001), spatial perception (*p* < 0.001), and motor praxis (*p* < 0.001), as well as total scores (*p* = 0.006); effect estimates were not reported [35]. For the other subscales including visuomotor, thinking operation, memory, and attention/concentration, no differences were found (Table 2).

#### 3.6.2. Burden of Care

Two trials examined the effect of telehealth versus face-to-face intervention on burden of care [25,33]. A carer program including education and problem-solving skills for carers of adults with dementia delivered at home with or without VC revealed no significant differences between groups in carer confidence and perceptions of care with the Caregiving Mastery Index or the Perceived Change Scale [33]. Similarly, the Bobath program, proprioceptive neuromuscular facilitation and electromyography-triggered neuromuscular stimulation for people living with dementia (home-based VC or outpatient setting), showed no differences post-intervention or at a 24-week follow-up for caregivers on the Caregiver Strain Index [25] (Table 2).

#### 3.6.3. Motor Outcomes

Three studies investigated motor outcomes in people with stroke delivered at home via VC or in an outpatient setting [25,30,32]. There were no between-group differences post-intervention for the modified Barthel Index or upper and lower limb muscle activity after physical therapies and electromyography-triggered neuromuscular stimulation [25], the Fugl-Meyer assessment after motor therapy and stroke education [32], or the Motor Activity Log—Arm Use Subscale and Wolf Motor Function Test (measuring motor arm capacity) after CIMT [30]. Sanford and colleagues compared VC with in-person home visits by occupational therapists and physiotherapists for adults with new mobility aids [34]. Although there was a significant improvement for the face-to-face group only on mobility self-efficacy scores, there were no differences between groups: MD = 3.10, 95%CI: −6.6, 12.80, *p* = 0.53.

### 3.7. Design and Effects of Physiotherapy Interventions

Twenty-six trials (29 reports) examined face-to-face versus telehealth physiotherapy intervention; three are described above as they also included occupational therapists. There were a wide variety of interventions which included single or combined interventions; not all were reported in detail. Interventions included aerobic exercise (including cardiac and pulmonary rehabilitation programs), strength training, balance training, flexibility exercises, and functional and/or gait retraining.

Eight trials included telehealth-supervised exercise [27,39,43,46,48,54,57,58], fourteen trials compared independent exercise with follow-up telehealth for monitoring and progression [36,37,38,40,41,42,43,44,47,50,51,59,60] and one trial was a combination of both [58]. Three trials were based at home for face-to-face interventions [41,42,53], one in a residential aged-care facility [27], and the remainder were center-/outpatient-based.

#### 3.7.1. Joint Range of Motion

Five trials reported joint range of motion across musculoskeletal populations [29,41,42,53,58]. Two trials reported no significant differences between groups for shoulder range abduction [42] and all joints measured [58] (Table 3). The pooled data from two trials revealed no difference between groups for active knee flexion (MD = −0.23 degrees, 95%CI: −1.96, 1.50; I^2^ = 0%) and knee extension (MD = 0.17 degrees, 95%CI: −0.80, 1.15; I^2^ = 44%) [29,53] whereas one trial observed greater gains in hip range in the telehealth group [41]. Three of the four trials that reported longer-term outcomes reported no significant between-group differences for the range of motion at long-term follow-up [29,42,53]; the fourth reported that 100% of burns patients receiving face-to-face intervention achieved a full range at 3 months compared with 70% who received telehealth interventions, *p* = 0.005 [58].

#### 3.7.2. Strength

Four trials in musculoskeletal populations reported on strength outcomes with varied results [29,36,53,58] (Table 3). Two trials reported comparable improvements in knee extension [53,58] and grip strength [58] between groups, which remained at a long-term follow-up. Conversely, two trials reported greater improvement in knee extension strength [29,36] in favor of telehealth, in one of which these differences were maintained at a 3-month follow-up [29].

#### 3.7.3. Pain

**Figure 3:**
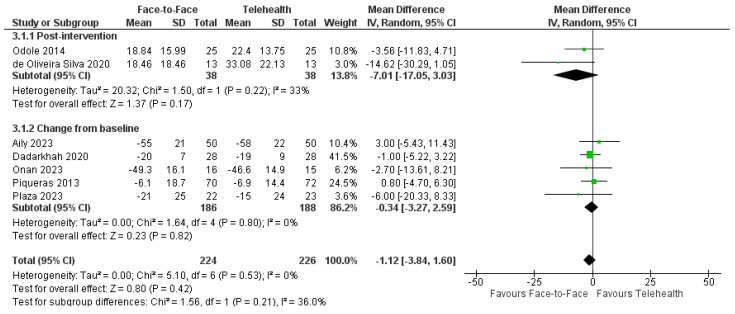
Physiotherapy for musculoskeletal conditions: Pain Visual Analogue Scale (VAS: 0–100 mm) [29,36,40,54,55,57,58].

Ten trials assessed pain outcomes and reported similar results when comparing telehealth with face-to-face physiotherapy for musculoskeletal conditions [29,36,40,41,42,53,54,55,57,58]. The data pooled from seven trials (*n* = 450) utilizing the Visual Analogue Scale (VAS; 0–100 mm) for pain revealed no statistically or clinically significant difference between telehealth and face-to-face therapy in reducing pain across a variety of musculoskeletal conditions, MD = −1.12 mm, 95%CI: −3.84, 1.60; I^2^ = 0% (Figure 3). The three trials that were not included in the meta-analyses (as they utilized a variety of subscales) also showed consistent results with no differences between groups on pain outcomes [41,42,53]. Long-term VAS change scores were pooled from three musculoskeletal trials (*n* = 278) and revealed no significant difference between delivery modes for VAS pain scores: MD = −3.10 mm 95%CI: −13.95, 7.76; I^2^ = 71%, *p* = 0.58 [29,36,58].

#### 3.7.4. Gait and Physical Activity

Overall, trials that investigated gait/walking time and physical activity outcomes reported no significant differences between telehealth and face-to-face physiotherapy (Table 3). Two trials reported using the 10-Meter Walk Test time and Self-Paced Walk Test and both reported no significant differences between delivery modes post-intervention [43,48] or at follow-up [48]. The pooled data of gait speed from three trials (*n* = 182) revealed comparable results with telehealth and face-to-face interventions: MD = −0.02 m/s, 95%CI −0.31, 0.27; I^2^ = 69% [36,44,60]. Two trials reported gait speed at a long-term follow-up (*n* = 117) with pooled data revealing no significant difference between groups: MD = −0.16, 95%CI −0.35, 0.04, I^2^ = 0%, *p* = 0.12 [36,60]. There were no differences between delivery modes in the freezing of gait in adults with PD post-intervention or long-term [44] or for the Performance Orientated Mobility Assessment—Gait subscale in adults post-stroke [51]. One trial reported on physical activity levels post-intervention and long-term and demonstrated no significant difference between telehealth and face-to-face cardiac rehabilitation [49].

#### 3.7.5. Balance

**Figure 4:**
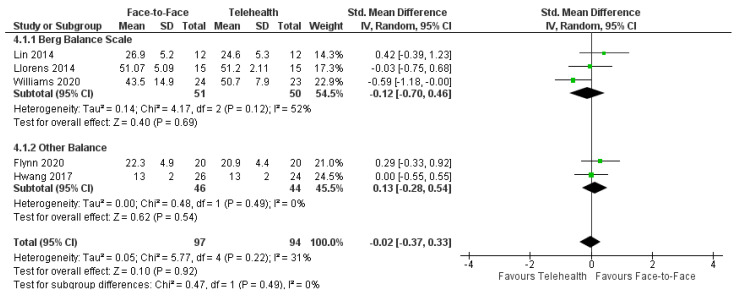
Physiotherapy for neurological conditions: balance outcomes for face-to-face compared to telehealth interventions [27,44,48,51,60].

Balance outcomes were measured in five trials using the Berg Balance Scale [27,51,60], Mini-BESTest [44], and the Balance Outcome Measure for Elder Rehabilitation [48]. A meta-analysis of these trials (*n* = 191) revealed no significant difference between face-to-face and telehealth for balance outcomes: SMD = −0.02, 95%CI −0.37, 0.33; I^2^ = 31% (Figure 4). Three trials reported no significant between-group differences for balance outcomes at a long-term follow-up [48,51,60].

##### Exercise Capacity

**Figure 5:**
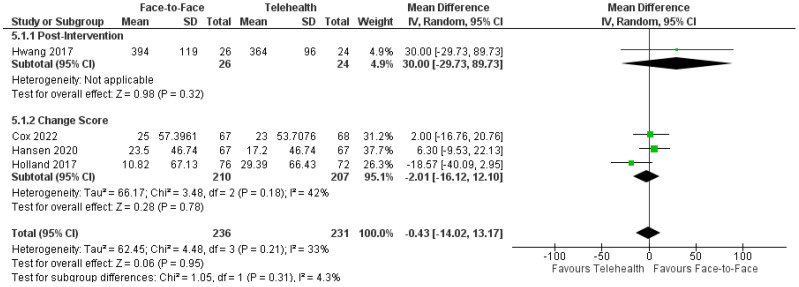
Physiotherapy for cardiopulmonary conditions: 6 Minute Walk Test Distance for face-to-face versus telehealth interventions [39,46,47,48].

Six trials reported functional exercise capacity in neurological, cardiopulmonary, and musculoskeletal populations using the distance walked during a 6 Minute Walk Test [39,46,47,48,53,60]. The pooled data from four cardiopulmonary trials (*n* = 467) revealed no significant difference between delivery modes for distance post-intervention: MD = −0.43 m, 95%CI: −14.02, 13.17; I^2^ = 33% (Figure 5) and for a long-term follow-up: MD = −3.86 m, 95%CI: 21.57, 13.85, I^2^ = 32% [39,46,47,48]. The remaining two trials, not included in the meta-analysis due to intervention heterogeneity, both reported no between-group differences post-intervention or at a follow-up in adults with MS [60] and knee arthroplasty [53]. Comparable effects were also reported on physical fitness outcome measures in two trials for face-to-face and telehealth intervention: Peak VO_2_, MD = −0.36 mL/min/kg, 95%CI: −2.57, 1.85; I^2^ = 0%, and peak workload, MD = −7.01 watts, 95%CI: −26.58, 12.57; I^2^ = 0% [37,49]. Fatigue severity was measured in one trial in adults with MS with a greater reduction in fatigue for face-to-face over telehealth delivery [59].

#### 3.7.6. Function and Disability

Function and disability were reported using a variety of measures (Table 1). Thirteen musculoskeletal trials reported no difference in function or disability between modes of delivery as measured by the Anterior Knee Pain Scale [54], Constant-Murley and QuickDash [42], Hip Disability and Osteoarthritis Outcome Score [41], Ibadan Knee/Hip Osteoarthritis Outcome Measure [55], Knee Injury and Osteoarthritis Outcome Score [38,53], Neck Disability Index, [57], Oswestry Disability Questionnaire [40], and Western Ontario and McMaster Universities Osteoarthritis Index (WOMAC) [29,36,43,53]. Likewise, there were no differences between face-to-face and telehealth interventions in neurological populations using the Barthel Index [27] and the Functional Independence Measure (FIM) [59].

A pooled analysis of Timed Up and Go times from three musculoskeletal trials and one cardiopulmonary trial (*n* = 281) revealed a non-significant trend favoring telehealth: MD = 4.36 s, 95%CI: −0.03, 8.76; I^2^ = 95%, with significant heterogeneity [29,41,43,48]. Three trials reported a long-term follow-up, with a pooled analysis demonstrating no significant difference between delivery modes: MD = 0.92 s, 95%CI: −4.40, 6.23; I^2^ = 92% [29,41,48].

#### 3.7.7. Anxiety and Depression

Symptoms of anxiety and depression were reported in three cardiopulmonary rehabilitation trials (*n* = 347) using the Hospital Anxiety Depression Scale (HADS) [39,47,49]. The pooled data revealed comparable outcomes for a reduction in HADS-depression: MD = 0.28, 95%CI: −0.35, 0.92; I^2^ = 0%), which remained comparable longer-term: MD = −0.08, 95%CI: −0.81, 0.66; I^2^ = 17%.

For HADS-anxiety, the pooled data revealed a non-significant moderate effect size favoring telehealth over face-to-face: MD = 0.64, 95%CI: −0.04, 1.32; I^2^ = 0%, *p* = 0.07. There were no significant between-group differences at a long-term follow-up: MD= 0.39, 95%CI: −0.33, 1.11; I^2^ = 0% [39,47,49].

#### 3.7.8. Health-Related Quality of Life

**Figure 6:**
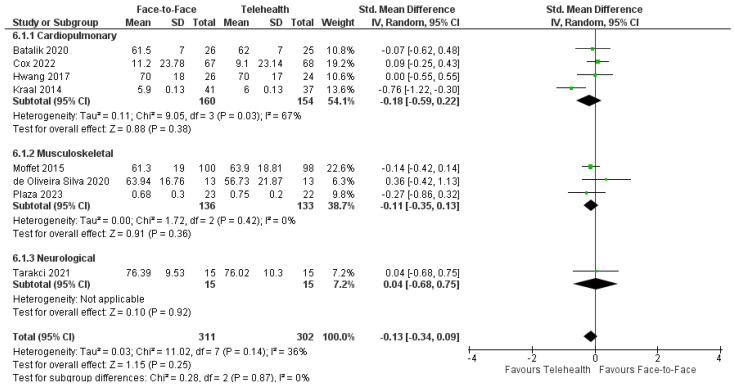
Physiotherapy across all conditions: Health-Related Quality of Life total scores for face-to-face compared to telehealth intervention [37,39,48,49,53,58,59].

Health-Related Quality of Life (HRQoL) was assessed using a variety of outcome measures in fifteen physiotherapy trials (Table 1). Variations in the way data were reported limited meta-analyses; however, the pooled data of total HRQoL post-intervention scores from eight trials (*n* = 613) [37,39,48,49,53,54,58,59] revealed no significant difference between delivery modes: SMD = −0.13; 95%CI: −0.34, 0.09; I^2^ = 36% (Figure 6). All of the trials not included in the meta-analysis due to insufficient data/reporting of total scores revealed no differences between face-to-face and telehealth groups, including reported HRQoL subdomains [38,41,43,46,47,56]. Seven trials reported a long-term follow-up [39,46,47,48,50,53,54]. A pooled analysis of the available data from five trials (*n* = 484) [39,48,50,53,54] revealed SMD = −0.53, 95%CI: −1.40, 0.34; I^2^ = 95% with high heterogeneity unexplained by differences in interventions. Two trials not included in the meta-analysis also reported no between-group differences in change scores at follow-up [46,47].

### 3.8. Design and Effects of Psychology Interventions

Interventions delivered by psychologists using telehealth compared with face-to-face were investigated in seventeen trials (19 reports); [28,31,61,62,63,64,65,66,67,68,69,70,71,72,73,74,75,76,77]. Most trials evaluated Cognitive Behavioral Therapy (CBT) or components of CBT [28,31,62,63,64,65,69,71,72,73,75,77], or Cognitive Processing Therapy (CPT) [61,67,70,76]. Five trials delivered telehealth via telephone [28,31,62,65,77] and the remainder were via VC.

Six trials (7 reports) included adults with Post Traumatic Stress Disorder (PTSD) [63,67,70,71,74,75,76], four included adults with depression [28,62,65,69], and the remaining trials included adults with cancer [68,77], bulimia nervosa [72,73], chronic or medically unexplained pain [61,64], and carers of people living with dementia [31,66].

#### 3.8.1. Depression

Nine trials evaluated the effects of CBT or components of CBT on depression [28,31,62,64,65,69,71,72,77]. A meta-analysis of these trials (*n* = 848) revealed a small non-significant effect size favoring face-to-face CBT over telehealth for reducing the symptoms of depression (SMD = −0.13, 95%CI: −0.27, 0.01; I^2^ = 5%) (Figure 6). There were no significant differences between delivery modes at a long-term follow-up (*n* = 742) (SMD = −0.13, 95%CI: −0.23, 0.19; I^2^ = 49% [28,31,62,64,69,73].

**Figure 7:**
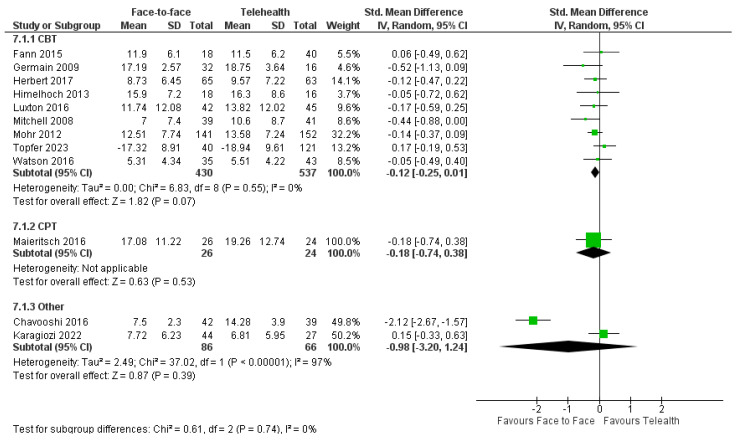
Psychology: depression outcomes for face-to-face compared to telehealth intervention [28,31,61,62,63,64,65,66,69,70,73,77].

Four trials evaluated the effects of psychotherapy, CPT, and other psychological treatments on depression [61,66,68,70]; available data from three trials (Figure 7) showed no significant difference between delivery modes [61,66,70]. The fourth trial reported no difference between groups in HADS-depression scores with positive psychotherapy for cancer survivors [68]. 

A sensitivity analysis revealed a significant but smaller effect on depression favoring face-to-face treatment: SMD = −0.18, 95%CI: −0.36, −0.01; I^2^ = 0%, [28,64,73].

#### 3.8.2. Anxiety

**Figure 8:**
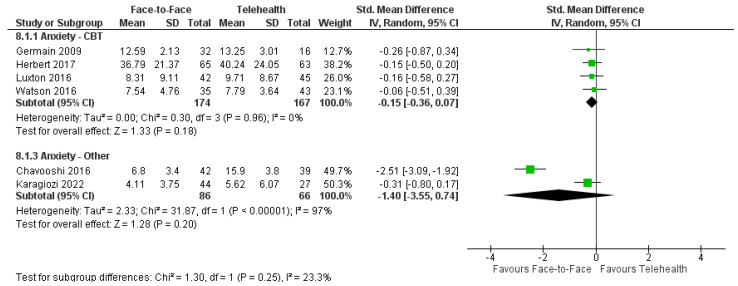
Psychology: anxiety outcomes for face-to-face compared to telehealth intervention [61,63,64,66,69,77].

Seven trials reported on anxiety symptoms using the Beck Anxiety Inventory [63,66,69], DASS-21 [61], HADS [68,77], and the Pain Anxiety Symptoms Scale-Short Form (PASS-20) [64]. Five trials reported significant reductions in anxiety for both delivery modes for adults with chronic and medically unexplained pain [61,64], cancer [77], PTSD [63], and caregivers of people with dementia [66]. A meta-analysis of four trials using CBT interventions (*n* = 341) revealed no significant difference between modes of delivery: SMD = −0.15, 95%CI: −0.36, 0.07; I^2^ = 0% (Figure 8) [63,64,69,77]. Three trials utilized non-CBT-based approaches [61,66,68]. Available data from two of these trials (*n* = 152) also revealed no significant difference between groups for anxiety outcomes, with high heterogeneity: SMD = −1.40, 95%CI: −3.55, 0.74; I^2^ = 97% [61,66]. The third trial in adults with cancer reported a significant reduction in anxiety with group telehealth delivery only [68]. Three trials reported on long-term follow-ups: one trial reported significantly greater reductions in anxiety with face-to-face psychotherapy [61] and two trials reported no significant between-group differences [64,69].

#### 3.8.3. PTSD

**Figure 9:**
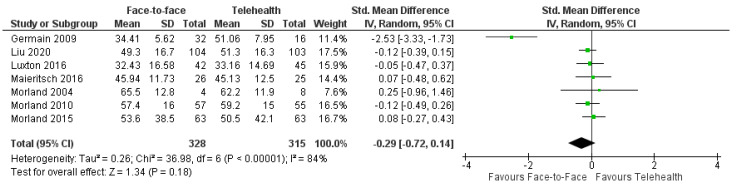
Psychology: PTSD symptoms for face-to-face compared to telehealth intervention [63,67,69,70,74,75,76].

The effect of psychology intervention on PTSD symptoms was investigated in eight trials using CBT [63,69,75], CPT [67,70,76], or other psychotherapy [68,74]. All trials reported reductions in PTSD symptoms and a pooled analysis of seven trials (*n* = 642) revealed SMD = −0.29, 95%CI: −0.72, 0.14; I^2^ = 84% (Figure 9). Four of these trials reported long-term results which also showed no between-group differences: MD = 0.12, 95%CI: −0.07, 0.30; I^2^ = 0% [67,69,70,74]. The one trial which was not included in the meta-analysis due to differences in reporting also found no between-group differences [68].

#### 3.8.4. Pain

Pain was reported in two trials using the Numeric Pain Rating Scale [61] and the Brief Pain Inventory Severity subscale [64]. The data were pooled using post-intervention and follow-up data (*n* = 209): SMD = −0.59, 95%CI: −1.31, 0.13; I^2^ = 84%, *p* = 0.11.and SMD = −0.75, 95%CI: −2.12, 0.61, I^2^ = 95%, *p* = 0.28, respectively. These moderate and large effect sizes favored face-to-face intervention, although this difference was not statistically significant possibly due to low power.

#### 3.8.5. Binge Eating and Purging

A trial of CBT for people with bulimia nervosa found similar reductions in binge eating and purging behavior with no difference between delivery modes, although the authors noted improvements occurred faster with face-to-face therapy [73].

#### 3.8.6. Health-Related Quality of Life

Five trials measured HRQoL and the results were inconsistent [31,61,63,64,73]. In adults with chronic pain [64] or bulimia nervosa [73], comparable results in HRQoL were reported post-intervention and at a longer-term follow-up for telehealth and face-to-face delivery. Two trials reported no significant differences between delivery modes for HRQoL with the exclusion of the physical domain, which favored telehealth [31,63]. These differences were not maintained at a 6-month follow-up in either trial. One trial reported a significantly greater improvement in HRQoL outcomes with face-to-face over telehealth as measured by the Quality of Life Inventory (*p* < 0.001) [61].

### 3.9. Design and Effects of Speech Pathology Interventions

Five trials reported on the outcomes of speech and language therapy in adults with chronic stuttering [78], Parkinson’s Disease [83], traumatic brain injury [80,81,82], and post-stroke speech and language difficulties including aphasia and cognitive-linguistic communication disorders [79,84] (Table 4). A wide range of interventions were utilized, including education and problem-solving [74], supported conversation techniques for partners [79,80,81,82], the Lee Silverman Voice Treatment [83], and semantic verification and picture naming [84].

#### 3.9.1. Cognition–Communication

One trial assessed measures of cognitive and linguistic function in adults post-stroke using the Cognitive-Linguistic Quick Test and reported no significant differences between delivery modes for all sub-scores including language and executive function [79] (Table 4).

#### 3.9.2. Communication

One trial investigated conversation techniques delivered center-based, in-person, or home-based via VC to adults with aphasia and cognitive-linguistic communication disorders. There were greater improvements in participant-reported communication confidence in favor of face-to-face delivery in both adults with aphasia and cognitive-linguistic communication disorders: MD = 2.61, 95%CI: 0.48, 4.74, *p* = 0.02 and MD = 2.90, 95%CI: 0.37, 5.43, *p* = 0.02, respectively [79]. Patient-reported communication ability was investigated in one trial of TBIconneCT training via home visits or home-based VC with no significant differences between delivery modes [80].

Communication partner ratings were investigated in three trials. All found no significant difference between delivery modes for PD-associated dysarthria [83], aphasia [79], cognitive-linguistic communication disorders [79] and TBI [80], except for the transaction subscale in Measure of Participation in Conversation where there were greater improvements in face-to-face participants over telehealth participants (mean(SD) face-to-face: 2.31(0.66) versus telehealth: 19(0.68), *p* = 0.03) [80]. This latter trial reported no differences between groups on these outcomes at the 3-month follow-up [80].

Aphasia was assessed in two trials using the Western Aphasia Battery—Revised, Part A [79] and word retrieval in conversation and spoken picture naming [84]. One trial reported no significant difference between face-to-face and telehealth intervention in Western Aphasia Battery—Revised scores [79]. The other trial investigated telehealth delivered by clinicians based at a university lab versus an outpatient setting versus face-to-face face. There were differences between the two telehealth groups: outpatient-delivered telehealth resulted in better word retrieval compared to university-delivered telehealth, and face-to-face therapy scored more highly than university-delivered telehealth. However, further analysis of reported data revealed that there were no differences between telehealth overall (combined groups) and face-to-face intervention: MD = −2.80 (−16.84, 11.24), *p* = 0.70. (*p* = 0.05) [84].

Two trials investigated telehealth and face-to-face speech and language therapy on fluency in people with chronic stuttering [78] and voice function outcomes in people with PD-associated dysarthria [83]. There were no differences between groups as measured by stuttering frequency, self-reported fluency, and stuttering severity rating scale post-intervention, with no difference between groups 9 months after randomization [78]. For voice functions, there were similar significant improvements in monologue and sustained phonation, and reading sound pressure levels (dB), with no difference between groups [83].

#### 3.9.3. Health-Related Quality of Life

One trial investigated HRQoL in adults with PD and reported no significant difference between delivery modes as measured by the Dysarthria Impact Profile and the Parkinson’s Disease Questionnaire [83].

### 3.10. Design and Effects of Podiatry Interventions

Although there were trials evaluating wound care and diabetic foot ulcer management via telehealth, the care teams did not include podiatrists or other allied health professionals and were therefore not included [85,86].

### 3.11. Allied Health: Adverse Events, Adherence, and Satisfaction

#### 3.11.1. Study-Related and Possibly Study-Related Adverse Events

There were no significant differences in rates or the severity of adverse events between face-to-face and telehealth occupational therapy in the three trials that reported on this outcome [25,32,33]. Similarly, ten physiotherapy trials also reported no significant difference between delivery modes for the occurrence or severity of adverse events [37,39,41,42,44,47,49,53,54,60]. The pooled data of study-related and possibly study-related events from four trials revealed that RR = 1.01, 95%CI: 0.36, 2.89; I^2^ = 29% [37,41,48,53]. No major events were reported. Two trials reported the occurrences of diagnosis-related hospitalizations during the trial and follow-up period [39,56]. One reported that four telehealth participants and two face-to-face participants experienced a respiratory-related hospitalization (4% of the participant group) [39] and the other reported that two telehealth participants and eight face-to-face participants were hospitalized for cardiac reasons [56]. Four psychology trials reported on the presence of adverse events [28,62,69,75]; one reported that one of the 42 telehealth participants required further evaluation for distress during the trial [69]. No adverse events were reported in the remaining trials. Adverse events were not reported in neuropsychology nor speech pathology trials.

#### 3.11.2. Adherence

**Figure 10:**
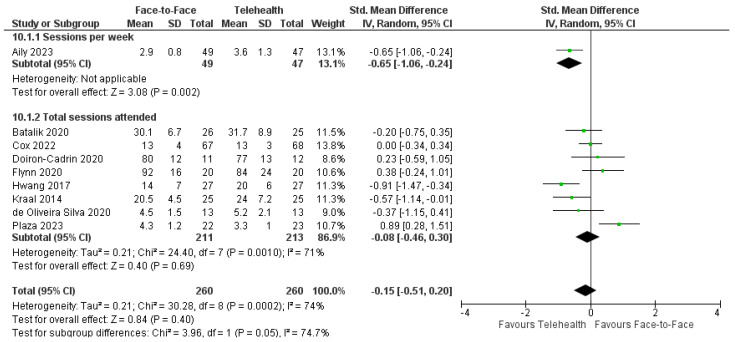
Physiotherapy: adherence to sessions for face-to-face compared to telehealth intervention [36,37,39,43,44,48,50,54,58].

For occupational therapy trials, one trial reported similarly high levels of adherence to sessions with no difference between delivery modes [32] and one trial reported that adherence was significantly higher in the telehealth group [34]. Meta-analyses of available data from nine physiotherapy trials (*n* = 520) revealed no significant differences in attendance between telehealth and face-to-face delivery: SMD = −0.15, 95%CI: −0.51, 0.20; I^2^ = 74% (Figure 10) [36,37,39,43,44,48,50,54,58]. Three physiotherapy trials had insufficient data to be included in the meta-analysis [42,47,60]. One trial had significantly poorer adherence to telehealth compared with face-to-face [60], whereas two trials reported significantly higher adherence to telehealth than face-to-face [42,47]. For psychology interventions, five trials reported no significant difference between groups for adherence [61,62,65,75,76]. One trial reported greater adherence to telehealth over face-to-face whereas one trial found greater attrition with telehealth compared with face-to-face psychology [64]. Adherence to speech therapy was reported in one trial with similar high attendance rates (≥84%) across both groups; around 94% of those who attended completed all training sessions [82].

#### 3.11.3. Satisfaction

**Figure 11:**
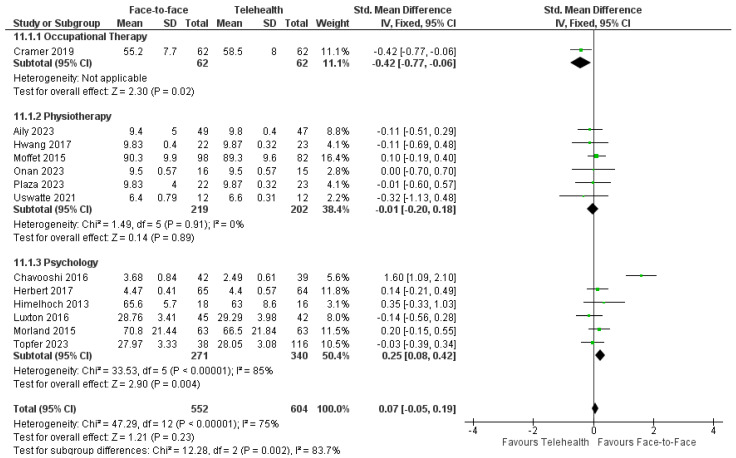
Occupational therapy, physiotherapy, and psychology: satisfaction for face-to-face compared to telehealth intervention [30,31,32,36,48,53,57,58,61,64,65,69,76].

Patient and/or clinician satisfaction was reported in one neuropsychology trial [26], one occupational therapy trial [32], twelve physiotherapy trials [27,30,36,38,41,43,44,48,49,52,57,58], nine psychology trials [31,61,62,64,65,69,74,75,76] and two speech pathology trials [78,84]. Overall, most trials reported no difference between face-to-face and telehealth interventions for patient satisfaction [27,30,36,48,52,57,58] and satisfaction levels were moderate to high [32,61,62,64,65,69,75]. Available data from thirteen trials (occupational therapy, physiotherapy, and psychology, *n* = 1175) were pooled to reveal no significant difference between delivery modes in satisfaction levels: SMD = −0.07, 95%CI: −0.05, 0.19; I^2^ = 85% (Figure 11). Although there was insufficient data for the meta-analysis in speech pathology trials, satisfaction ratings were also comparable across telehealth and face-to-face [78,84], with the exception of one subscale item where telehealth was significantly more frequently rated as ‘extremely convenient’ compared with face-to-face participant responses [78]. The remaining allied health trials with insufficient data for the meta-analysis reported higher satisfaction for telehealth over face-to-face physiotherapy [49], higher satisfaction for face-to-face physiotherapy over telehealth [44], and two trials reported no significant difference between delivery modes for psychology intervention [62,75]. Four trials surveyed telehealth participants only, with all reporting high levels of satisfaction for neuropsychology [26] and physiotherapy [38,41,43] delivered via telehealth. Clinician satisfaction was investigated in two trials: physiotherapists providing telehealth reported high levels of satisfaction [58] and psychologists reported similar high levels of satisfaction for both telehealth and face-to-face psychology interventions [74].

### 3.12. Feasibility of Telehealth in Allied Health

Fourteen trials reported that the provision of allied health interventions for various populations via telehealth was feasible [26,27,33,34,35,43,44,54,58,67,69,75,80,84]. Some trials optimized the feasibility of telehealth by providing initial in-person support for the set-up of telehealth equipment/environment/familiarization with the intervention [26,30,32,33,34,35,38,41,43,49,53,58,83], in-person support for the facilitation of the intervention or engagement with a remote therapist [26,27,34], and technical assistance as required [41,51,58,66,68,69,74,76,84]. Additionally some trials approved participants’ own devices [58,80] or offered backup options for VC software (SkypeV^®^ (Microsoft Corporation, Redmond, WA, USA) and Facetime^®^ (Apple, Cupertino, CA, USA)) [43] or telephone. Hardware, software, and internet connectivity issues were tolerated well [29,58,83,84]. One trial reported that 25% of sessions had minor technical issues and 8% had major technical issues and were unable to be completed. Despite these technical problems, satisfaction with telehealth remained high [58]. Another trial reported that although connectivity issues occurred only sporadically, they were mostly experienced by those using their mobile phone network which was affected by factors such as location, weather, and internet traffic [83]. For some participants, partner/carer support was vital when using technology and/or correctly donning remote monitoring devices [41,84]. Two trials only included participants who had high-speed internet [53,68] and three trials included participants’ existing devices for VC [35,58,80].

The therapist time was reported in three trials. For synchronous telehealth, the time taken to deliver therapy was lower for telehealth participants (10 min per telehealth participant versus 98 min per face-to-face participant) [44]. Another trial reported that travel time was reduced for telehealth participants (M = 255.9 min versus M = 77.2 min, *p* < 0.0001) [33]. Total therapist time was also significantly reduced in asynchronous telehealth (M = 6.5 h, IQR 1.2 versus M = 32.1 h, IQR = 5.2, *p* < 0.01) [41].

## 4. Discussion

The findings of this rapid review demonstrate that telehealth can be successfully implemented as an alternate delivery mode to face-to-face allied health interventions for occupational therapists, physiotherapists, psychologists, and speech pathologists for some conditions in adults. Overall, a wide range of allied health interventions delivered via telehealth resulted in similar outcomes as comparable interventions delivered via face-to-face. These included outcomes such as balance after stroke [27,51,60], walking distance [47,48,53,60], HRQoL [37,38,47,48,53,54,56,61,64,71], and communication [78,79,84]. Most trials were comparable in terms of patient satisfaction, adherence, and attendance. Allied health delivered via telehealth demonstrated similar rates of adverse events as interventions provided face-to-face.

Effectiveness studies of podiatry interventions delivered via telehealth have been reported to be acceptable to residents of aged care facilities [87] and show positive outcomes for wound and diabetic foot ulcer management [85,86]. However, no RCTs that included podiatrists in the care team were identified for inclusion in the present review. Therefore, further robust research using RCT methods to evaluate the benefits and risks of telehealth use by podiatrists is still required.

Imposed travel and service restrictions in response to COVID-19 led to a forced rapid uptake of telehealth as an acceptable and viable modality for the delivery of health services [88,89], with good levels of satisfaction reported by patients and a range of healthcare providers during COVID-19 restrictions [90,91]. The continued delivery of treatment using telehealth-based models of care may be particularly beneficial for people with mobility limitations, chronic health conditions, living remotely, or with limited ability to travel. Only a small number of trials specifically relied on a partner/carer or assistant presence for support with the intervention; therefore, the presence of a carer should not be a barrier to offering allied health intervention via telehealth. Telehealth affords greater access for patients to clinicians with the potential for improving health outcomes in patients who may otherwise miss out. For example, in patients with chronic pain, the intensity and nature of treatment in an outpatient pain clinic differed in relation to the distance that patients had traveled; telehealth may afford similar intensity and nature of treatment regardless of location [20,92]. When considering convenience, flexibility, and outcomes for patients, allied health professionals may incorporate telehealth as a single or mixed model of care [93].

Although it has been suggested that telehealth may be limited to those with access to a high-speed internet connection [43], the range of technology options reported in this review demonstrates the potential to cater to an individual’s needs and their existing technology while maintaining healthcare data security [69].

### Limitations

Limitations to this review include the use of a rapid review process; however, we followed the Cochrane [18] criteria for rapid reviews and integrated technological tools to expedite several processes. The authors acknowledge that this rapid review process may have resulted in missing relevant RCTs published prior to 2017 that were not included in any systematic review, hence impacting the results. The use of machine learning technology to support citation screening, and text mining for the first stage of the full-text review of systematic review eligibility, may have resulted in the omission of relevant trials. Trials and reviews in languages other than English were excluded, which may have influenced the results and likely made our findings most relevant to service provision in English-speaking countries. Future trials could consider including trials in languages other than English. Trial authors were not contacted for additional data which may have limited the scope of the evidence synthesis, particularly for meta-analyses. We limited dual review for trial selection which may have led to missing relevant trials. In addition to screening systematic reviews and reference lists of included reports, several databases were searched from 2017 forward. This approach was taken to reduce duplication of previous work undertaken by other systematic review authors, and a complete search of RCTs from database inception was not undertaken. However, given that 361 systematic reviews were identified that examined telehealth treatments, with the most recent searches conducted in 2017, it would be unlikely that these reviews all missed relevant trials conducted prior to 2017. Nonetheless, the possibility of missing published RCTs not included in a systematic review prior to 2017 is acknowledged which may have influenced the results. There were insufficient studies to allow for a comparison of the differences in the effectiveness of telehealth by modality. In addition, there was a wide variety of interventions tested, and potential variations in efficacy across different types of interventions were not explored. This meta-analysis was limited to a subset of included studies, which limits the generalizability of findings and highlights the need for the provision of all trial results in telehealth research. Allied health disciplines were limited to physiotherapy, occupational therapy, psychology, speech pathology, podiatry, and neuropsychology; provision of telehealth by other allied health disciplines was not investigated. Future research should consider the inclusion of other allied health disciplines, differences between telehealth modalities, longer-term outcomes, and outcomes such as cost-effectiveness and clinician satisfaction.

## 5. Conclusions

This rapid systematic review provides evidence that telehealth interventions provided by occupational therapists, physiotherapists, psychologists, and speech pathologists result in similar increases in walking, balance, HRQoL, depression and anxiety symptoms, and communication ability as face-to-face interventions across a range of clinical presentations. Clinicians should be confident in using telehealth interventions for clients who have a preference for this modality, or who may not otherwise be able to access treatment. Few trials were identified that evaluated the efficacy of telehealth for interventions commonly provided by occupational therapists, podiatrists, and neuropsychologists in areas such as self-care training, cognitive rehabilitation, behavioral management, and podiatry interventions. Further RCTs are needed to address these gaps in knowledge, ideally conducted in partnership with clinician–researchers who have increased their clinical use of telehealth modalities during the COVID-19 pandemic.

## Figures and Tables

**Table 1:** Summary of included trials.

Study	Participant Characteristics Number of Participants Mean Age, Years (SD) Sex (F/M) Diagnosis/Population	Intervention and Comparator	Outcome Measures
***Neuropsychology***			
Jelcic 2014 [26]	*n* = 17 Age = 84.1 (5.7) Sex = 12 F, 5 M Early-stage Alzheimer’s disease	Lexical-semantic stimulation to enhance semantic verbal processing FTF = Day center-based group therapy (3–4 participants), 60 min daily Telehealth = Day center-based group VC (3–4 participants) via Skype^®^ (Microsoft Corporation, Redmond, WA, USA) and computer program with exercises, trained operator present to facilitate interaction with therapist, 60 min daily Duration = 3 months	Attention and executive functions (DCT and TMT) Feasibility Global cognitive performance (MMSE) Lexical-semantic abilities (VNT and phonemic and semantic fluency) Memory (FDST, ROCF, Brief Story Recall, RAVLT) Satisfaction Visual-spatial abilities (ROCF Copy Test)
***Occupational Therapy***			
Chen 2017 [25]	*n* = 54 Age = 61.2 (12.1) Sex = 21 F, 33 M Stroke with hemiplegia	Rehabilitation exercise (Bobath and PNF) and electromyography-triggered neuromuscular stimulation FTF = Outpatient setting, exercises 1 h × per weekday and ETNS 20 min × 2 per weekday Telehealth = Home-based VC, training logs, same program as FTF Duration = 12 weeks (60 sessions total)	Adverse events Balance (BBS) Caregiver Strain (CSI) Disability (MRS) Function (MBI)
Laver 2020 [33]	*n* = 63 Age = 70.3 (14.5) (carers) 80 (6.8) (adults with dementia) Sex = 25 F, 38 M Dementia, carers of adults with dementia	Carer program to problem solve, educate, and build skills FTF = Home visits, approximately 60 min Telehealth = 2 home visits followed by home-based VC via h264 videoconference codecs (Tandberg 550 MXP; Cisco Systems, San Jose, CA, USA) on laptop, tablet, or smartphone, ≤6 sessions Duration = 16 weeks, ≤8 sessions	Adverse events Caregiver mastery (CMI) Function (CAFU) Perceived change (PCS)
Torpil 2023 [35]	*n* = 68 Age = 70.0 (2.7) Sex = 10 F, 24 M Amnesic mild cognitive impairment	Cognitive rehabilitation FTF = Center-based, 2 × 45 min sessions per week Telehealth = Home-based VC via Skype^®^ (Microsoft Corporation, Redmond, WA, USA), Zoom (Zoom Video Communications, San Jose, CA, USA), or WhatsApp Messenger (Meta Platforms, Mountain View, CA, USA) according to participants’ preferences and existing technology, 2 × 45 min sessions per week Duration = 12 weeks	Cognitive skills (LOTCA-G) Feasibility
***Occupational Therapy and Physiotherapy***			
Cramer 2019 [32]	*n* = 124 Age = 61.0 (14.0) Sex = 38 F, 90 M Stroke—within 3 months of stroke onset	Arm motor therapy plus stroke education FTF = Outpatient setting with 18 supervised and 18 unsupervised 70 min sessions, use of standard exercise hardware for functional tasks Telehealth = Home-based VC as per FTF, content matched with FTF; use of 12 input devices (e.g., PlayStation^®^ Move controller (Sony, Japan) or trackpad) for functional tasks Duration = 30 days	Adverse events Motor skills (FM)
Sanford 2006 [34]	*n* = 32 Age 61.4 (12.9) Sex = 5 F, 27 M Community-dwelling older adults with new mobility devices	Mobility and transfer tasks FTF = Home visits, 1 × 60 min session per week of PT/OT Telehealth = Home-based VC operated by a research assistant in the home 1 × 60 min session per week with remote PT/OT Duration = 4 weeks, 4 sessions	Mobility self-efficacy (FES) Self-reported physical functioning
Uswatte 2021 [30]	*n* = 24 Age = 59.6 (13.2) Sex = 10 F, 14 M Chronic stroke	Upper Limb Constraint-Induced Movement Therapy FTF = In lab, 1:1 sessions 3.5 h per day for 10 consecutive weekdays Telehealth = VC 1:1 sessions at home using Tele-AutoCITE (developed in C#.NET 2007 (Microsoft, Redmond, WA, USA), as per FTF Duration = 2 weeks, 10 sessions	Arm Use (MAL) Motor capacity (WMFT) Satisfaction
***Physiotherapy***			
Aily 2023 [36]	*n* = 100 Age = 55.0 (8.0) Sex = 60 F, 40 M Knee osteoarthritis	Circuit training of upper and lower limb, trunk, and global exercises FTF = Center-based group exercise, 3 sessions per week Telehealth = Home-based asynchronous exercise 3 sessions per week via video recordings (DVD), with 20 min telephone calls every 1–3 weeks (total of 7) Duration = 14 weeks	Adherence, acceptability Body composition (fat, lean mass, muscle architecture) Gait speed (40 m fast-paced walk test) Pain (VAS and Pain Catastrophizing Syndrome) Pain, stiffness, and functional capacity (WOMAC, stair climb test, 30-second chair stand test) Strength (isometric peak torque)
Batalik 2020 [37]	*n* = 56 Age = 57.1 (7.2) Sex = 14F, 42M Cardiovascular disease and myocardial revascularization	Cardiac rehabilitation including exercise and educational booklet FTF = Center-based group exercise and education, 3 × 60 min sessions per week Telehealth = Home-based asynchronous exercise, 3 × 60 min sessions per week, weekly telephone feedback as recommendations, advice, and motivation Duration = 12 weeks, 36 sessions	Adherence Adverse events Health-Related Quality of Life (SF-36) Physical fitness (maximal CPET text)
Bini 2017 [38]	*n* = 29 Age = 63.3 (NR) Sex = 13 F, 15 M Total knee arthroplasty	Exercise protocol (customized type/number and frequency of exercises) FTF = Outpatient setting Telehealth = Home-based asynchronous video application (CaptureProof, San Francisco, CA, USA) on iPod touch (Apple, Cupertino, CA, USA) and web-based interface; participants videoed themselves performing exercises which were reviewed with feedback and progression by physiotherapist. Outpatient physiotherapy was available Duration = Minimum 3 months, sessions as required	Function (KOOS-PS) Health-Related Quality of Life (VR-12) Pain (VAS) Range
Cox 2022 [39]	*n* = 142 Age = 67.5 (9.0) Sex = 76 F, 66 M Chronic respiratory disease	Pulmonary rehabilitation including exercise physiologists and nurses, at least 30 min of lower limb aerobic training at 60% Peak VO2 plus progressive resistance training. Home exercises 3 sessions per week (unsupervised) with home diary, self-management education (book and brochure for online resources) FTF = Center-based, group sessions (8–12 participants) Telehealth = Home-based VC using iPad (Apple, Cupertino, CA, USA) and Zoom (Zoom Video Communications, San Jose, CA, USA) with initial home visit by physiotherapist, followed by group sessions (4–6 participants), stationary exercise bicycle, pulse oximeter Duration = 8 weeks, 16 sessions total	Adverse events Anxiety and depression (HADS) Change in dyspnoea (Modified Medical Research Council scale) Functional exercise capacity (6MWT) Health-Related Quality of Life (CRQ) Hospitalization Psychological well-being Self-efficacy Symptoms
Dadarkhah 2021 [40]	*n* = 56 Age = 49.5 (8.9) Sex = 32 F, 24 M Chronic and non-specific low back pain	Core stability, flexibility, and strengthening program with logbook FTF = Clinic-based exercise, 3 sessions per week Telehealth = Home-based asynchronous exercise 2 sessions per day for 4 weeks, 3 × 10 min telephone calls per week Duration = 4 weeks, 12 sessions	Disability (ODQ) Pain (VAS)
Dias Correia 2019 [41]	*n* = 66 Age = 64.4 (9.2) Sex = 31 F, 35 M Arthroscopic rotator cuff repair	Shoulder strengthening and range of movement exercises FTF = Home visits for supervised exercise 3 × 30–60 min sessions per week plus ≥ 2 sessions unsupervised per week (30 sessions) Telehealth = Home-based asynchronous exercise via app, 5 × 15–30 min per week, digitally monitored via inertial motion trackers (chest, upper arm, and wrist) and progressed via PT through web portal. Plus, 13 × home visits by PT Duration = 8 weeks	Adherence Adverse events Shoulder function (QuickDASH)
Dias Correira 2022 [42]	*n* = 50 Age = 61.7 (6.9) Sex = 39 F, 11 M Total hip arthroplasty	Hip strengthening and range of movement exercises FTF = Home visits for supervised exercise 3 × 60 min sessions per week plus ≥ 2 sessions unsupervised per week Telehealth = Home-based exercise via app 5–7 ×x ≥ 30 min sessions per week, asynchronous monitoring by physiotherapist. Telephone call weeks 2 and 6, with FTF visit week 4, additional home visits as required Duration = 8 weeks	Adherence Adverse events Hip function (HOOS) Hip range Mobility (TUG) Patient satisfaction
Doiron-Cadrin 2020 [43]	*n* = 23 Age = 65.6 (9.5) Sex = 17 F, 6 M Awaiting total hip or knee replacement	Lower limb strengthening, proprioception, cardiovascular warm-up and education, with exercise logbook FTF = Outpatient setting, 2 sessions per week, with independent exercise program 3 sessions per week Telehealth = Home-based supervised exercise 1:1 VC through web platform REACTS LiteVR^®^ (Technologies innovatrices d’imagerie, Montreal, QC, Canada) on iPad (Apple, Cupertino, CA, USA) 2 × week plus independent exercise program as per FTF. Backup VC options of Skype^®^ (Microsoft Corporation, Redmond, WA, USA) and FaceTime^®^ (Apple, Cupertino, CA, USA) Duration = 12 weeks	Feasibility Function (Lower Extremity Functional Scale, WOMAC, TUG, GRC, Stair test) Gait speed (self-paced walk) Satisfaction
Flynn 2020 [44]	*n* = 40 Age = 72 (6.9) Sex = 10 F, 30 M Parkinson’s Disease	Exercises in 2 phase study: first phase center-based, second phase split into FTF and telehealth groups. FTF = Center-based group exercises 3 × 60 min sessions per week Telehealth = Home-based with prescribed exercises from PhysioTherapy eXercises website (https://www.physiotherapyexercises.com), 3 × 45–60 min sessions per week (unsupervised), telephone calls to monitor and progress Duration = 5 weeks	Adherence Acceptability Feasibility
Hansen 2020 [45,46]	*n* = 134 Age = 68.3 (9.0) Sex = 74 F, 60 M Severe COPD	Pulmonary rehabilitation including nurses, Structured exercise and education with activity monitor (activePAL) (PAL Technologies Ltd., Glasgow, UK) FTF = Outpatient group program, 2 × 60 min sessions per week Telehealth = Home-based group VC on single touch screen device, 3 × 35 min sessions per week Duration = 10–12 weeks	Activity levels (steps per day) Anxiety and depression (HADS) Function (30 second-STS) Functional exercise capacity (6MWT) Health-Related Quality of Life (EQ-5D)
Holland 2017 [47]	*n* = 166 Age = 69 (11.5) Sex = 67 F, 99 M COPD	Pulmonary rehabilitation (aerobic and resistance exercise and self-management education) FTF = Center-based group program, 2 sessions per week Telehealth = Home-based: first week goal setting and supervision of exercise, ≥30 min of unsupervised aerobic training on most days of week. Weekly telephone call by physiotherapist including motivational interviewing. Education component, pedometer, and exercise diary Duration = 8 weeks	Adverse events Anxiety and depression (HADS) Attendance Dyspnea (modified Medical Research Council dyspnoea scale) Functional exercise capacity (6MWT) Health-Related Quality of Life (CRQ) Self-efficacy (PRAISE)
Hwang 2017 [48]	*n* = 53 Age = 67.5 (12.3) Sex = 13 F, 40 M Chronic heart failure	Cardiac rehabilitation including exercise and multidisciplinary education FTF = Hospital-based (outpatient) group program Telehealth = Home-based group VC (Adobe Connect 9.2) (Adobe, Systems Inc., San Jose, CA, USA) on laptop with chat function. Pre-recorded education as slides with audio. Telephone technical support was available. Self-monitored vital signs (equipment supplied) Duration = 12 weeks, 2 sessions per week	Attendance Adverse events Balance (BOOMER) Functional exercise capacity (6MWT) Functional outcomes (TUGT) Health-Related Quality of Life (EQ-5D) Strength (grip, quadriceps) Patient satisfaction
Kraal 2014, 2017 [49,50]	*n* = 90 Age = 59.2 (8.5) Sex = 10 F, 80 M Cardiac disease—low-moderate risk after myocardial infarction, unstable angina or revascularization procedure	Cardiac rehabilitation including exercise specialists FTF = Center-based group training at 70–85% maximal heart rate, 2–3 × 45–60 min sessions per week Telehealth = Home/personal gym-based training at 70–85% maximal heart rate, ≥3 × 45–60 min sessions per week. First 3 sessions were outpatient supervised. Weekly telephone calls by physiotherapist or exercise specialist for goal setting, feedback/progression, and motivational interviewing. Web application (Garmin Connect) and heart rate monitor (Garmin Fore-runner 70) (Garmin™, Kansas, MO, USA) Duration = 12 weeks	Adherence Adverse events Exercise capacity (Peak VO_2_) Health-Related Quality of Life (SF-36) Physical activity level (PAEE)
Lin 2014 [27]	*n* = 17 Age = 75.1 (2.9) Sex = 7 F, 10 M Stroke with proximal active movement in upper extremity in hemiparetic side, living in long-term care facilities	Standing balance training including volunteers FTF = Care-facility-based group physiotherapy (2 people), 3 × 50 min sessions per week Telehealth = Group VC (2 people) with 3D animation exercise videos and interactive games (National Taiwan University and Lunghwa University of Science and Technology, Taiwan) with touch screen capabilities exercises matched as per FTF, 3 × 50 min sessions per week, vital signs monitored with bi-directional sensor devices. Volunteer assisting on participant-end guided by remote physiotherapist Duration = 4 weeks, 12 sessions	Balance (BBS) Function (Barthel Index) Satisfaction
Llorens 2015 [51]	*n* = 30 Age = 55.5 (8.4) Sex = 13 F, 17 M Stroke with residual hemiparesis (stroke onset >6 months)	Virtual-Reality balance and complementary exercises with motion-sensing device (Microsoft Kinect) (Microsoft Corporation, Redmond, WA, USA) FTF = Clinic-based VR 3 × 45 min sessions per week plus 2 sessions per week conventional physiotherapy Telehealth = Home-based VR (laptop and television), 3 × 45 min sessions per week plus clinic-based conventional physiotherapy 2 sessions per week, weekly interview to progress Duration = 7 weeks, 20 sessions	Balance (BBS) Cost Gait (POMA-G) Mobility (POMA-B) Motivation (IMI) Usability (SUS)
Moffet 2015, 2017 {Moffet, 2017 #20647;Moffet, 2015 #20646}	*n* = 205 Age = 66.0 (8.0) Sex = 105 F, 100 M Total knee arthroplasty	Progressive exercise program of mobility, strength, function, and balance, with home exercise program and education FTF = Home visits, 2 × 45–60 min sessions per week Telehealth = Home-based VC sessions on h264 videoconference codecs (Tandberg 550 MXP; Cisco Systems, San Jose, CA, USA), 2 × 45–60 min sessions per week Duration = 2 months, 16 sessions	Adverse events Function, symptoms, and Quality of Life (KOOS) Functional exercise capacity (6MWT) Knee Range Knee Strength Pain, stiffness, physical function (WOMAC) Satisfaction
Odole 2013, 2014 {Odole, 2014 #16766;Odole, 2013 #16765}	*n* = 50 Age = 55.5 (7.6) Sex = 24 F, 26 M Osteoarthritis of the knee	Structured Exercise (not specified) FTF = Clinic-based exercise 3 sessions per week Telehealth = Home-based asynchronous exercise 3 sessions per week (as per FTF), with structured telephone monitoring and coaching 3 sessions per week, logbook Duration = 6 weeks, 18 sessions	Disability Function (IKHOAM) Health-Related Quality of Life (WHOQoL-Bref) Pain (VAS)
de Oliveira Silva 2020 [54]	*n* = 35 Age = 31.5 (5.9) Sex = 27 F, 8 M Patellofemoral pain, 6 weeks after self-management exercise program	Education and exercise, content as per ‘My Knee Cap’ website (mykneecap.trekeducation.org) FTF = Private practice clinic Telehealth = VC via Skype^®^ (Microsoft Corporation, Redmond, WA, USA) Duration = 12 weeks, maximum of 8 sessions	Adverse Events Disability (AKPS) Feasibility Knee self-efficacy (K-SES) Pain (VAS and PCS) Quality of Life (Knee Injury Quality of Life subscale) Recovery (GROC)
Onan 2023 [57]	*n* = 31 Age = 38.5 (10.7) Sex = 22 F, 9 M Neck pain	Spinal stabilization exercises FTF = Clinic-based 3 sessions per week Telehealth = Home-based VC and video exercises, 3 sessions per week Duration = 8 weeks, 24 sessions	Disability (NDI) Neck function (Neck functional capacity evaluation test, Neck Awareness Questionnaire) Neck muscle size Pain (VAS) Satisfaction
Piqueras 2013 [29]	*n* = 181 Age = 73.3 (6.5) Sex = 131 F, 50 M Total knee arthroplasty	TKA Rehabilitation Clinical Protocol FTF = Outpatient setting—60 min sessions each weekday for 10 days Telehealth = Home-based 60 min sessions each weekday for 10 days, with interactive virtual software–hardware platform; in total, 5 sessions supervised by physiotherapist, 5 sessions at home with remote monitoring and progression, telephone contact if necessary. Wireless sensor WAGYRO (Wireless accelerometer and gyroscope) (Shimmer Research, Dublin, Ireland) to monitor knee range and movement Duration = 2 weeks, 10 sessions	Knee range Muscle strength (kg) Mobility (TUG) Pain (VAS) Pain, stiffness, and functional capacity (WOMAC)
Plaza 2023 [58]	*n* = 45 Age = 46.8 (15.0) Sex = 14 F, 31 M Burns ≤ 25% Total Body Surface Area	Individualized including range of motion and strengthening exercises, education, and home exercise program FTF = Outpatient-based, 30–60 min 1:1 sessions Telehealth = Home-based VC using eHAB^®^ (NeoRehab, Brisbane, Australia) with patient’s existing technologies, 30–60 min 1:1 sessions, as per FTF Duration = 6 weeks, range 3–12 sessions (minimum 1 per fortnight—maximum 2 per week)	Adherence Burn-scar impact (BBSIP) Grip and quadriceps strength Feasibility Health-Related Quality of Life (AQoL-4D) Joint range (degrees) Pain (VAS) Patient and therapist satisfaction Self-efficacy for exercise Technical disruptions
Tarakci 2021 [59]	*n* = 30 Age = 40.3 (10.7) Sex = 23 F, 7 M Relapsing-Remitting Multiple Sclerosis	Structured exercise program of progressive resistance exercise with PNF, stretching, balance, coordination, and ambulation FTF = Center-based exercise, 3 × 60 min supervised sessions per week (non-consecutive days) Telehealth = Home-based exercise, 3 × 60 min sessions per week, with VC calls to monitor adherence and progression, plus center-based session 1 per month to check/revise exercises Duration = 12 weeks, 36 sessions	Fatigue (FSS) Function (FIM) Health-Related Quality of Life (NHP, QoLS)
Williams 2020 [60]	*n* = 50 Age = 52 (10.5) Sex = 38 F, 12 M Multiple Sclerosis	Progressive functional and balance training FTF = Center-based group sessions, 2 × 60 min sessions per week, at least 2 days apart Telehealth = Home-based exercise 2 × 60 min sessions per week (independent), with activity diary and telephone support by a physiotherapist each fortnight Duration = 8 weeks, 16 sessions	Adherence Adverse events Balance (BBS) Functional walking capacity (6MWT) Gait speed (10MWT)
***Psychology***			
Chavooshi 2017 [61]	*n* = 81 Age = 32 (6.2) Sex = 29 F, 52 M Medically unexplained pain	Intensive short-term dynamic psychotherapy, based on CPT FTF = Clinic-based, 1 × 60 min session per week Telehealth = Home-based VC via Skype^®^ (Microsoft Corporation, Redmond, WA, USA), 1 × 60 min session per week Duration = 16 weeks, 16 sessions	Anxiety and depression (DASS-21) Emotion regulation (ERQ) Pain intensity (NPRS) Health-Related Quality of Life (QOLI) Stress (MAAS)
Fann 2015 [62]	*n* = 58 Age = 45.4 (14.1) Sex = 24 F, 34 M Major depression after Traumatic Brain Injury	CBT with workbook and homework, encouraged to attend with support persons FTF = Center-based, 1 × 30–60 min session per week, personalized mailed follow-up letter with mutually agreed-upon exercises, workbook, and homework Telehealth = Home-based telephone calls, as per FTF Duration = 12 weeks, 12 sessions	Adverse events Depression (HAMD-17, SCL-20) Satisfaction
Germain 2009 [63,71]	*n* = 68 Age = 42.1 (12.1) Sex = 44 F, 24 M PTSD	CBT FTF = Center-based, 1 x 60 min session per week Telehealth = Center-based VC and fax (to send/receive written material during therapy), 1 × 60 min session per week Duration = 16–25 weeks	Anxiety (BAI) Depression (BDI-II) Function (ACF) Health-Related Quality of Life (SF-12) PTSD (MPSS)
Herbert 2017 [64]	*n* = 128 Age = 52 (13.3) Sex = 23 F, 105 M Chronic pain in veterans	Acceptance and Commitment Therapy FTF = Medical center/outpatient clinic, 1 × 60 min session per week Telehealth = VC (individual) at clinic of their choice–1 × 60 min session per week Duration = 8 weeks, 8 sessions	Activity level/disability/function (MPI) Anxiety—pain-related (PASS-20) Depression Health-Related Quality of Life (SF-12) Pain (BPI, CPAQ) Satisfaction (CSQ) Sleep quality (PSQI)
Himelhoch 2013 [65]	*n* = 31 Age = 45.1 (8.3) Sex = 25 F, 6 M Population = People living with HIV/AIDS with major depression	CBT FTF = Clinic-based–10 × 60 min sessions Telehealth = Telephone CBT —10 × 60 min sessions Duration = 14 weeks	Adverse events Depression (HAM-D) Satisfaction (SIMH)
Karagiozi 2022 [66]	*n* = 82 Age = 44.5 (10.6) Sex = not stated Caregivers of people living with dementia	Psychoeducational Program FTF = Day Center Group Sessions, 1 × 60 min session per week Telehealth = Home-based Group VC, 1 × 60 min session per week Duration = 4 months, 16 sessions	Anxiety (BAI) Carer Burden (ZBI) Depression (BDI)
Liu 2020 [67]	*n* = 207 Age = 48.4 (14.1) Sex = 45 F, 154 M PTSD in veterans	CPT: Cognitive, including social workers and counsellors FTF = VA Hospital-based, 1 × 60 min 1:1 session per week Telehealth = Veterans Affairs outpatient setting VC, 1 × 60 min 1:1 session per week Duration = 12 weeks, 12 sessions	Depression (PHQ-9) PTSD (CAPS, PCL-S)
Lleras de Frutos 2020 [68]	*n* = 269 Age = 49.9 (8.6) Sex = 269 F, 0 M Cancer in women	Positive Psychotherapy for Cancer FTF = Center-based group therapy (8–12 patients) 1 × 90–120 min session per week Telehealth = Home-based group VC (5–6 patients) via ViTAM (Video Teleassistance and Monitoring, University of Girona, Girona, Spain); 1 × 90–120 min session per week and 1 × FTF session (final) Duration = 12 weeks, 12 sessions	Adherence Anxiety and Depression (HADS) PTSD (PCL, PTGI)
Luxton 2016 [69]	n = 121 Age = 45% aged 19–29 Sex = 22 F, 99 M Depression in veterans	Behavioral Activation Treatment for Depression (a component of CBT) FTF = Clinic-based, 1 session per week Telehealth = Home-based VC, 1 session per week Duration = 8 weeks, 8 sessions	Adverse events Anxiety (BAI) Depression (BDI-II) Hopelessness (BHS) PTSD (PCL) Satisfaction (CSQ-8)
Maieritsch 2016 [70]	*n* = 90 Age = 30.9 (6.1) Sex = 7 F, 84 M PTSD	CPT including social workers FTF = Hospital setting, 1–2 × 50 min 1:1 sessions per week Telehealth = VA Hospital, VC with clinician at a distant VA hospital, 1–2 × 50 min 1:1 sessions per week Duration = 18 weeks	Depression severity PTSD (CAPS)
Mitchell 2008, Marrone [72,73]	*n* = 128 Age = 29.0 (10.6) Sex = 126 F, 2 M Bulimia nervosa	CBT FTF = Center-based, 60 min sessions Telehealth = VC at a distal site, 60 min sessions Duration = 16 weeks, 20 sessions	Absence of binge eating and purging Depression (HAM-D, BDI) Health-Related Quality of Life (SF-36)
Mohr 2012 [28]	*n* = 325 Age = 47.7 (13.0) Sex = 252 F, 73 M Depression	CBT with workbook FTF = Clinic-based sessions, 45 min sessions (2 in first 2 weeks, then weekly for 12 weeks, and 2 sessions over 4 weeks) Telehealth = Home-based telephone calls, same as FTF Duration = 18 weeks, 18 sessions	Adverse events Attendance Depression (HAM-D, PHQ-9)
Morland 2004 [74]	*n* = 18 Age = Not stated, inclusion criteria range 18–60 Sex = M PTSD in veterans	Psychoeducation for coping skills FTF = Veterans Center, group sessions Telehealth = Veterans Center, VC group sessions, with backup clinician on-site and technician available for technical problems Duration = 8 weeks, 8 sessions	Information retention PTSD (PCL) Satisfaction—patient and clinician
Morland 2010 [75]	n = 125 Age = 54.7 (9.6) Sex = 125 M PTSD in combat veterans	Anger management therapy (CBT) FTF = VA clinical site, Group sessions, 2 sessions per week Telehealth = VA clinical site group VC sessions with remote therapist, 2 sessions per week Duration = 6 weeks, 12 sessions	Adherence Adverse events Anger (NAS, STAXI-2) PTSD (CAPS, PCL) Satisfaction (CPOSS-VA)
Morland 2015 [76]	*n* = 126 Age = 46.4 (11.9) Sex = 126 F PTSD in women	CPT FTF = VA clinical site, 1–2 × 90 min 1:1 sessions per week Telehealth = VA clinical site VC with remote psychologist, 1–2 × 90 min 1:1 sessions per week Duration = 6 weeks, 12 sessions	Adherence PTSD (CAPS) Satisfaction (CPOSS-VA, TSAS) Treatment engagement (TEQ)
Topfer [31]	*n* = 188 Age = 63.5 (11.4) Sex = 150 F, 38 M Caregivers of people living with dementia	CBT with extended Tele.TAnDem FTF = Home visits, 50 min Telehealth = Home-based telephone sessions, 50 min Duration = 6 months, 12 sessions	Caregiver burden (BEHAVE-AD) Depression (CES–D) Emotional wellbeing (VAS) Health complaints Health-Related Quality of Life (WHOQoL-BREF) Satisfaction (CSQ-8)
Watson 2017 [77]	*n* = 118 Age = 50.4 (13.3) Sex = 85 F, 33 M Cancer	CBT with workbook and CD FTF = Center-based Telehealth = Home-based via telephone Duration = 12 weeks, up to 8 sessions	Anxiety and depression (HADS) Helpless/hopelessness (MAC) Patient-reported outcomes (PRO) Satisfaction
**Speech Pathology**			
Carey 2010 [78]	*n* = 40 Age = 65% 18–30 years old Sex = 7 F, 33 M Chronic stuttering	‘Camperdown Program’ FTF = Center-based ‘Camperdown Program’—individual teaching, group practice, individual problem solving, and maintenance Telehealth = Home-based via telephone, adapted ‘Camperdown Program’ (content as per FTF), mailed audiotapes, voicemail for speech samples, feedback by SP as needed, and home practice Duration = Not specified	Fluency (% syllables stuttered) Patient satisfaction
Meltzer 2017 [79]	*n* = 44 Age = 64.2 (10.8) Sex = 17 F, 27 M Communication disorders with chronic stroke	Supported conversation techniques to partners FTF = Center-based, 1 × 60 min session per week Telehealth = Home-based VC via WebEx^®^ (Cisco, San Jose, CA, USA) or VSee (VSee, San Jose, CA, USA), 1 × 60 min session per week at home or telehealth site, supported with iPad (Apple, Cupertino, CA, USA) with TalkPath Software (Lingraphica Inc., Princeton, NJ, USA; Steele et al., 2014) for exercises/home program Duration = 10 weeks, 10 sessions	Communication (confidence and effectiveness) (WAB-R, CCRSA, CETI) Cognition (CLQT)
Rietdijk 2020 [80,81,82]	*n* = 36 Age = 54 (range 20–68), 42 (range 19–66) Sex = 31 F, 5 M Moderate to severe Traumatic Brain Injury with social communication skills deficits	TBIconneCT training with participant and communication partner, with manual and email summary of session content FTF = Home visits, TBIconneCT training, 1 × 90 min 1:1 session per week Telehealth = Home-based TBIconneCT training delivered via VC using Skype^®^ (Microsoft Corporation, Redmond, USA) on their own home computer, 1 × 90 min 1:1 session per week Duration = 10 weeks, 10 sessions	Adherence Attendance Conversation Function (FAVRES)
Theodoros 2016 [83]	*n* = 31 Age = 71.0 (8.8) Sex = 10 F, 21 M Parkinson’s Disease	Lee Silverman Voice Treatment FTF = Clinic-based, 4 × 60 min sessions per week Telehealth = Home-based VC using eHAB^®^ (V2.0, NeoRehab, Brisbane, Australia) with acoustic software and microphone, treatment as per FTF Duration = 4 weeks, 16 sessions	Acoustic Measures Health-Related Quality of Life (Dysarthria Impact Profile, PDQ-39) Perceptual measures
Woolf 2016 [84]	*n* = 20 Age = 59.7 (12.1) Sex = 6 F, 14 M Chronic post-stroke aphasia following left hemisphere stroke	Semantic verification, picture naming, and self-administered practice FTF = University lab, 2 × 60 min sessions per week plus computer-based homework Telehealth = Home-based VC via FaceTime^®^ (Apple, Cupertino, CA, USA) using iPad (Apple, Cupertino, CA, USA), workbook, 2 × 60 min sessions per week computer-based homework. Therapist was either university-based or at a clinical site Duration = 4 weeks, 8 sessions	Compliance and satisfaction Feasibility Word retrieval

6MWT—6 Minute Walk Test, 10MWT—10 Meter Walk Test, 30 second STS—30 Second Sit to Stand, ACF—Assessment of Current Functioning, AQoL-4D—Assessment of Quality of Life-4D, BAI—Beck Anxiety Inventory, BBS—Berg Balance Scale, BBSIP—Brisbane Burn Scar Impact Profile, BDI—Beck Depression Inventory, BEHAVE-AD—Behavioral Pathology in Alzheimer’s Disease Rating Scale, BHS—Beck Hopelessness Scale, BOOMER—Balance Outcome Measure for Elder Rehabilitation, BPI— Brief Pain Inventory Short Form Interference Scale, CAFU—Caregiver Assessment of Function and Upset, CAPS—Clinician-Administered PTSD Scale, CBT—Cognitive Behavioral Therapy, CCRSA—Communication Confidence Rating Scale for Aphasia, CD—Compact Disc, CES-D—Center for Epidemiological Studies Depression Scale, CETI—Communicative Effectiveness Index, CLCD—Cognitive-Linguistic Quick Test, CLQT—Cognitive Linguistic Quick Test, CMI—Caregiver Mastery Index, CPET—Cardiopulmonary Exercise Testing, CPOSS-VA—Charleston Psychiatric Outpatient Satisfaction Scale-VA, CRQ—Chronic Respiratory Questionnaire, CSI—Caregiver Strain Index, CSQ-8—Client Satisfaction Questionnaire, CPT—Cognitive Processing Therapy, DASS-21—Depression and Anxiety Stress Scale-21, DCT—Digit Cancellation Test, ERQ—Emotional Regulation Scale, EQ-5D—EuroQol 5D, F—Female, FAVRES—Functional Assessment of Verbal Reasoning and Executive Strategies, FDST—Forward Digit Span Test, FES—Falls Efficacy Scale, FIM—Functional Independence Measure, FM—Fugl-Meyer score, FSS—Fatigue Severity Scale, FTF—Face-to-face, GRC—Global Rating of Change Scale, GROC—Global Rating of Change, HADS—Hospital Anxiety and Depression Scale, HAMD-17—Hamilton Depression Rating Scale-17 Item, HOOS—Hip Disability and Osteoarthritis Outcome Score, IKHOAM—Ibadan Knee/Hip Osteoarthritis Outcome Measure, IMI—Intrinsic Motivation Inventory, IQR—Interquartile Range, KOOS—Knee Injury and Osteoarthritis Score, K-SES—Knee Self Efficacy Scale, LOTCA-G—Loewenstein Occupational Therapy Cognitive Assessment, M—Male, MAAS—Mindful Attention Awareness Scale, MAC—Mental Adjustment to Cancer Scale, MAL—Motor Activity Log, Min—minutes, MBI—Modified Barthel Index, MMSE—Mini-Mental State Exam, MPI—West Haven-Yale Multidimensional Pain Inventory, MRS—Modified Rankin Scale, NAS—Novaco Anger Scale, NDI—Neck Disability Index, NHP—Nottingham Health Profile, NPRS—Numerical Pain Rating Scale, ODQ—Oswestry Disability Questionnaire, OT—Occupational Therapist, PAEE = Physical Activity Energy Expenditure, PASS-20—Pain Anxiety Symptoms Scale-Short Form, PCL—Post-Traumatic Stress Disorder Checklist, PCS—Perceived Change Scale, PDQ-39—Parkinsons Disease Questionnaire-39, PET—Prolonged Exposure Therapy, PHQ-9—Patient Health Questionnaire, PNF—Proprioceptive Neuromuscular Facilitation, POMA-B—Performance-Oriented Mobility Assessment Balance Subscale, POMA-G—Performance-Oriented Mobility Assessment Gait Subscale, PRAISE—Pulmonary Rehabilitation Adapted Index of Self-Efficacy, PRO—Patient Reported Outcomes, SQI—Pittsburgh Sleep Quality Index, PT—Physiotherapist, PTGI—Post Traumatic Growth Inventory, PTSD—Post-Traumatic Stress Disorder, QOLI—Quality of Life Inventory, QoLS—Quality of Life Scale, QuickDASH—Quick Disability of Arm, Shoulder and Hand, RAVLT—Rey Auditory Verbal Learning test, RMS—Root Mean Square of Muscle Contraction, ROCF—Rey–Osterrieth Complex Figure, SCL—Symptom Checklist-20, SF-12—Short Form-12, SF-36—Short Form 36, SIMH—Satisfaction Index-Mental Health, SP—Speech Pathologist, STAXI-2—State-Trait Anger Expression Inventory, SUS—System Usability Scale, Tele-Auto CITE—Tele-Automated Constraint-Induced Therapy Extension, TEQ—Treatment Expectancy Questionnaire, TKA—Total Knee Arthroplasty, TMT—Trail Making Test, TSAS—Telemedicine Satisfaction and Acceptance Scale, TUG—Timed Up and Go, VA—Veterans Affairs, VAS—Visual Analogue Scale, VC—Videoconferencing—Real Time, VNT—Verbal Naming Test, VR—Virtual Reality, VR-12—Veterans Rand 12 Item Survey, WHOQoL-BREF—World Health Organization Quality-of-Life Scale, WOMAC—Western Ontario and McMaster Universities Arthritis Index, WAB-R—Western Aphasia Battery-Revised, Part 1, WMFT—Wolf Motor Function Test.

**Table 2:** Results for telehealth versus face-to-face occupational therapy and combined occupational therapy and physiotherapy by outcome.

Outcome	Studies	Sample Size Telehealth	Sample Size Face-to-Face	Outcome Measure	Mean Difference between Groups (95%CI)	*p*-Value
Cognition	Torpil 2023 [35]	34	34	LOTCA-G Orientation Visual perception Spatial perception Motor praxis Visuomotor Thinking operation Memory Attention/concentration Total test	0.18 (−0.09, 0.45) 0.83 (0.55, 1.11) 0.67 (0.34, 1.00) 0.83 (0.49, 1.17) 0.20 (−0.53, 0.93) 0.15 (−0.22, 0.52) −0.02 (−0.42, 0.38) −0.09 (−0.33, 0.15) 2.73 (0.98, 4.48)	0.20 <0.001 * <0.001 * <0.001 * 0.59 0.43 0.92 0.45 0.002 *
Burden of Care	Laver 2020 [33]	25	27	Caregiving Mastery Index	0.09 (−1.26, 1.45)	0.891
	Laver 2020 [33]	26	27	Perceived Change Scale	0.07 (−1.31, 1.16)	0.905
	Chen 2017 [25]	27	27	Caregiver Strain Index	0.41 (−0.66, 1.49)	0.666
Motor outcomes	Chen 2017 [25]	27	27	Function—mBI	2.08 (−5.17, 9.34)	0.897
	Sanford 2006	16	16	Mobility—Falls Efficacy Scale	Effect estimates not reported	NR
	Chen 2017 [25]	27	27	Berg Balance Scale	0.92 (−1.27, 3.10)	0.912
	Chen 2017 [25]	27	27	Upper limb activity—mRS	2.33 (−8.86, 13.52)	0.901
	Cramer 2019 [32]	62	62	Upper limb activity—Fugl-Meyer assessment	0.06 (−2.14, 2.26)	NR
	Cramer 2019 [32]	62	62	Box and Block	0.95 (−1.90, 3.80)	0.512
	Cramer 2019 [32]	62	62	Stroke Impact Scale—hand motor domain	−5.19 (−11.80, 1.42)	0.124
	Chen 2017 [25]	27	27	Lower limb activity—RMS	5.08 (−11.11, 21.28)	0.583
	Uswatte 2021 [30]	12	12	Motor Activity Log—Arm Use Scale	0.20 (−0.78, 1.18)	0.69
	Uswatte 2021 [30]	12	12	Wolf Motor Function Test (repetitions per minute)	−0.30 (−4.72, 4.12)	0.89

* In favor of face-to-face intervention, LOTCA-G—Lowenstein Occupational Therapy Cognitive Assessment: Geriatric; mRS—Modified Rankin Scale; RMS—Mean Square; FTF—Face-to-face; NR—Not Reported.

**Table 3:** Results for immediate effects of telehealth versus face-to-face physiotherapy by outcome for outcomes not included in pooled analysis.

Outcome	Studies	Sample Size Telehealth	Sample Size Face-to-Face	Outcome Measure	Mean Difference between Groups (95%CI)	*p*-Value
Range of motion—active (degrees)						
	Dias Correia 2019 [41]	35	31	Hip Supine flexion Supine abduction Standing flexion Standing hyperextension Standing abduction	26.4 (13.32, 39.5) 15.1 (6.91, 23.25) 12.0 (1.81, 22.33) −10.1 (−15.75, −4.38) 14.1 (7.51, 20.76)	<0.001 * <0.001 * 0.02 * 0.001 * <0.001 *
	Dias Correia 2022 [42]	27	23	Shoulder Scapular elevation Shoulder flexion Shoulder abduction External rotation	6.69 (−15.00, 28.38) 6.10 (−13.32, 25.52) 25.70 (−2.18, 49.22) 2.92 (−6.78, 12.63)	0.53 0.53 0.03 * 0.54
	Moffet 2015 [53]	104	101	Knee Flexion Extension	1.0 (−2.4, 4.3) 0.1 (1.0, 1.2)	>0.05 >0.05
	Piqueras 2013 [29]	72	70	Knee Flexion Extension	−0.82 (−3.03, 1.39) 0.70 (−0.38, 1.78)	0.47 0.2
Strength						
	Piqueras 2013 [29]	72	70	Knee Extension Flexion	−1.34 (−2.29, −0.39) −0.48 (−1.27, 0.31)	0.006 * 0.24
	Plaza 2023 [58]	23	22	Knee extension (kg) Right Left	1.0 (−1.66, 3.66) 1.80 (−0.78, 4.38)	0.46 0.17
Function	Doiron-Cadrin 2020 [43]	11	11	Self-paced walk (seconds)	0.80 (−3.58, 5.18)	0.72
	Doiron-Cadrin 2020 [43]	11	11	Stair test	0.10 (−3.60, 3.80)	0.96

* In favor of telehealth.

**Table 4:** Results for telehealth versus face-to-face speech pathology.

Outcome	Studies	Sample Size		Outcome Measure	Mean Difference between Groups (95%CI)	*p*-Value
		Telehealth	Face-to-Face			
Cognition	Meltzer 2017 [79]	CLCD 5	6	Cognitive-Linguistic Quick Test Language Memory Executive Attention Visuospatial	1.0 (−3.20, 5.20) −2.30 (−22.99, 18.39) −1.50 (−5.18, 2.18) −23.10 (−49.97, 3.77) NR	0.64 0.83 0.42 0.09 NR
Communication	Meltzer 2017 [79]	Aphasia 14 CLCD 4	14 5	Communication Confidence Rating Scale for Aphasia Communication Effectiveness Index (partner rating)	2.61 (0.48, 4.74) 2.90 (0.37, 5.43) 6.38 (5.76)	0.02 * 0.02 * 0.87
	Theodoros 2016 [83]	15	16	Communication Partner Rating Easy to Understand Repetition Initiate with familiar Initiate with unfamiliar Overall rating	0.4 (−0.16, 0.96) 0.60 (−0.23, 1.43) 0.30 (−0.51, 1.11) 0.40 (−0.55, 1.35) 0.60 (−0.33, 1.53)	0.212 0.418 0.632 0.289 0.263
	Rietdijk 2020 [80]			Measures of Support in Conversation Reveal Competence CC Reveal Competence PC Acknowledge Competence CC Acknowledge Competence PC Measures of Participation in Conversation Interaction CC Interaction PC Transaction CC Transaction PC	−0.33 (−0.66, 0.00) −0.10 (−0.36, 0.16) −0.17 (−0.54, 0.02) −0.23 (−0.61, 0.15) −0.12 (−0.61, 0.37) −0.04 (−0.51, 0.43) −0.13 (−0.55, 0.,29) −0.12 (−0.32, 0.56)	0.05 0.45 0.37 0.24 0.63 0.87 0.54 0.59
	Meltzer 2017 [79]	14	14	Western Aphasia Battery	−2.61 (−4.74, −0.48)	0.02 *
	Woolf 2016 [84]	5/5 ‡ †	5	Clinical Site Content words per turn Nouns per turn University Site Content words per turn Nouns per turn	0.72 (−2.72, 1.28) −0.05 (−0.58, 0.48) −1.19 (−5.54, 3.16) −1.0 (−3.09, 1.09)	0.48 0.85 0.59 0.35
Voice Function	Theodoros 2016 [83]	15	16	Acoustic Measures Sustained Phonation (dB) Reading (dB) Monologue (dB) Maximum F_0_ range (Hz)	3.50 (1.22, 5.78) 2.30 (−1.19, 5.79) 0.70 (−3.00, 4.40) 42.20 (−5.32, 89.72)	0.443 0.388 0.596 0.296
	Carey 2010 [78]	20	20	Stuttering frequency Speech naturalness Self-reported stuttering severity Daily Talking to friends/family members	NR NR NR NR	0.9 0.24 0.7 0.2
Quality of Life	Theodoros 2016 [83]	15	16	Dysarthria Impact Profile Effect on person Acceptance Others’ reactions Communication with others Total score Parkinson’s Disease Questionnaire-39 Communication Activities of daily living Cognition Emotion Social support Stigma Bodily discomfort Mobility PQD-39 Summary Index	0.40 (−0.16, 0.96) 0.60 (−0.23, 1.43) 0.30 (−0.51, 1.11) 0.40 (−0.55, 1.35) 0.60 (−0.33, 1.53) −2.40 (−15.01, 10.21) −5.60 (−18.44, 7.24) 5.40 (−9.95, 20.75) 3.60 (−6.49, 13.69) 3.80 (−3.31, 10.91) −1.50 (−12.29, 9.29) 0.70 (−14.05, 15.45) −6.20 (−20.48, 8.08) −0.30 (−8.52, 7.92)	0.766 0.354 0.775 0.812 0.746 0.71 0.39 0.49 0.48 0.29 0.79 0.93 0.39 0.94

* Favored face-to-face; † 5 participants received telehealth from therapist at university site, 5 participants received telehealth from therapist at clinical site; ‡ Favored telehealth at clinic site; SD— Standard Deviation; CLCD—Cognitive-Linguistic Communication Disorder; CC—Casual Conversation; PC—Purposeful Conversation.

## Data Availability

All data contained within are as described.

## Supplementary Materials

The following supporting information can be downloaded at: https://www.mdpi.com/article/10.3390/healthcare12121217/s1, Table S1: Search Strategy, Table S2: Allied Health Dictionary for QDA Miner; Table S3: Excluded Full Texts.

## List of Abbreviations

HRQoL	Health-Related Quality of Life
CBT	Cognitive Behavioral Therapy
PTSD	Post-Traumatic Stress Disorder
CPT	Cognitive Processing Therapy
PET	Prolonged Exposure Therapy
