# Supplementary material for: Delivery of Allied Health Interventions Using Telehealth Modalities: A Rapid Systematic Review of Randomized Controlled Trials
Source: Healthcare (Basel). 2024 Jun 18;12(12):1217. doi: 10.3390/healthcare12121217 (PMC11203162; doi:10.3390/healthcare12121217)
Supplement: Supplementary file 1 [file healthcare-12-01217-s001.zip › healthcare-2967478-supplementary.pdf]

Supplementary Files– Table S1. Search Strategy

Ovid MEDLINE(R)– Systematic Reviews

1 telemedicine.mp.

2 exp Telemedicine/

3 exp Telemedicine/

4 telemetry.mp.

5 exp Videoconferencing/

6 telecommunications.mp.

7 remote consultation.mp.

8 remote sensing technology.mp

9 exp Telephone

10 electronic mail.mp

11 text messaging.mp.

12 computers.mp.

13 exp Cell Phone/

14 (tele-rehabilitation or telerehab or telehealth or tele-health or telehomecare or tele-homecare or telecoaching or tele-coaching or telecommunication* or videoconference$ or video-conferenc* or videoconsultation or video-consultation or telestroke or teleconference* or tele-conference* or teleconsultation or tele-consultation or telecare or ehealth or e-health).mp.

15 ((rehabilitation or therap* or treatment or communication or consultation) adj5 (telephone* or phone* or video* or internet* or computer* or sensor* or modem or webcam or website* or email)).mp

16 (remote* or distance* or distant).mp. adj5 ((rehabilitation or therap*).mp. or *interven*/ or treatment.mp. or physio*.mp. or physical therap*.mp. or occupational therap*.mp. or neuropsycholog*.mp. or psycholog*.mp. or speech therap*.mp. or speech patholog*.mp. or communication.mp. or podiatr*.mp. or consultation.mp. or care.mp. or specialist*.mp. or monitor*.mp. or virtual reality.mp. or virtual environment*.mp. or technolog*.mp.)

17 (mhealth or m-health or m health or mobile health).mp.

18 (smartphone or text-messag* or (tablet adj3 (device* or comput*))).mp.

19 ((cell* or smart* or mobile or android or internet or web) adj3 (comput* or device or app* or phone)).mp.

20 1 or 2 or 3 or 4 or 5 or 6 or 7 or 8 or 9 or 10 or 11 or 12 or 13 or 14 or 15 or 16 or 17 or 18 or 19

21 allied health.mp.

22	occupational therapy/ or occupational therap*.mp

23	(physiotherap* or physical therap*).mp.

24	speech therapy/ or speech therap*.mp.

25	podiatry/ or podiatr*.mp.

26	neuropsychology/ or neuropsycholog*.mp.

27	psychology/ or psycholog*.mp.

28	21 or 22 or 23 or 24 or 25 or 26 or 27

29	Systematic Review/

30	systematic review.ti.

31	meta-analys*.ti.

32	29 or 30 or 31

33	20 and 28 and 32

Cochrane Central Register of Controlled Trials – CENTRAL – Systematic Reviews

1	exp Telemedicine/

2	Telemetry/

3	exp Videoconferencing/

4	Telecommunications/

5	Remote Consultation/

6	Remote Sensing Technology/

7	exp Telephone/

8	Electronic Mail/

9	exp Internet/

10	Text Messaging/

11	Computers/

12	exp Microcomputers/

13	Minicomputers/

14	Cell Phone/

15	(telemedicine or telemetry or telerehabilitation or tele-rehabilitation or telerehab or telehealth or tele-health or telehomecare or tele-homecare or telecoaching or tele-coaching or telecommunication* or videoconference$ or video-conferenc* or videoconsultation or video-

consultation or telestroke or teleconference* or tele-conference* or teleconsultation or tele-consultation or telecare or ehealth or e-health).mp.

16 (telespeech or tele-speech or teleOT or tele-OT or telepractice or teletherap*).mp.

17 ((rehabilitation or therap* or treatment or communication or consultation) adj5 (telephone* or phone* or video* or internet* or computer* or sensor* or modem or webcam or website* or email)).mp.

18 ((remote* or distance* or distant) adj5 (rehabilitation or therap* or treatment or physio* or physical therap* or occupational therap* or neuropsycholog* or psycholog* or speech therap* or speech patholog* communication or consultation or care or specialist* or monitor* or virtual reality or virtual environment* or technolog*)).mp.

19 ((cell* or smart* or mobile or android or internet or web) adj3 (comput* or device or app* or phone)).mp.

20 (smartphone or text-messag* or (tablet adj3 (device* or comput*))).mp.

21 (mhealth or m-health or m health or mobile health).mp.

22 (tele adj3 (game* or game* or exergame* or virtual reality*)).mp.

23 1 or 2 or 3 or 4 or 5 or 6 or 7 or 8 or 9 or 10 or 11 or 12 or 13 or 14 or 15 or 16 or 17 or 18 or 19 or 20 or 21 or 22

24 allied health.mp.

25 Occupational Therapy/ or occupational therap*.mp.

26 (physiotherap* or physical therap*).mp. or Physical Therapy Modalities/

27 Speech Therapy/ or speech therap*.mp. or (speech adj 2 language therap*).mp. or speech patholog*.mp.

28 Psychology/ or psycholog*.mp.

29 neuropsycholog*.mp. or Neuropsychology/

30 Podiatry/ or podiatr*.mp.

31 24 or 25 or 26 or 27 or 28 or 29 or 30

32 "Systematic Review"/

33 review.m_titl.

34 (meta-analysis or meta-analyses).m_titl.

40 32 or 33 or 34

41 23 and 31 and 40

EMBASE – Systematic Reviews

1 exp Telemedicine/

2 telemetry.mp.

3 exp Videoconferencing/

4 telecommunications.mp.

5 remote consultation.mp.

6 remote sensing technology.mp.

7 exp Telephone/

8 electronic mail.mp.

9 text messaging.mp.

10 computers.mp.

11 exp Cell Phone/

12 (tele-rehabilitation or telerehab or telehealth or tele-health or telehomecare or tele-homecare or telecoaching or tele-coaching or telecommunication* or videoconference$ or video-conferenc* or videoconsultation or video-consultation or telestroke or teleconference* or tele-conference* or teleconsultation or tele-consultation or telecare or ehealth or e-health).mp.

13 ((rehabilitation or therap* or treatment or communication or consultation) adj5 (telephone* or phone* or video* or internet* or computer* or sensor* or modem or webcam or website* or email)).mp.

14 (remote* or distance* or distant).mp. adj5 ((rehabilitation or therap*).mp. or *interven*/ or treatment.mp. or physio*.mp. or physical therap*.mp. or occupational therap*.mp. or neuropsycholog*.mp. or psycholog*.mp. or speech therap*.mp. or speech patholog*.mp. or communication.mp. or podiatr*.mp. or consultation.mp. or care.mp. or specialist*.mp. or monitor*.mp. or virtual reality.mp. or virtual environment*.mp. or technolog*.mp)

15 (mhealth or m-health or m health or mobile health).mp.

16 (smartphone or text-messag* or (tablet adj3 (device* or comput*))).mp.

17 ((cell* or smart* or mobile or android or internet or web) adj3 (comput* or device or app* or phone)).mp.

18 Internet/

19 Microcomputers/

20 Minicomputers/

21 (telemedicine or telemetry or telerehabilitation or tele-rehabilitation or telerehab or telehealth or tele-health or telehomecare or tele-homecare or telecoaching or tele-coaching or

telecommunication* or videoconference$ or video-conferenc* or videoconsultation or video-consultation or telestroke or teleconference* or tele-conference* or teleconsultation or tele-consultation or telecare or ehealth or e-health).mp. 161326

22 (telespeech or tele-speech or teleOT or tele-OT or telepractice or teletherap*).mp. 4953

23 (tele adj3 (game* or game* or exergame* or virtual reality*)).mp. 31

24 1 or 2 or 3 or 4 or 5 or 6 or 7 or 8 or 9 or 10 or 11 or 12 or 13 or 14 or 15 or 16 or 17 or 18 or 19 or 20 or 21 or 22 or 23

25 allied health.mp.

26 Podiatry/ or podiatr*.mp.

27 neuropsycholog*.mp.

28 psycholog*.mp.

29 Occupational Therapy/ or occupational therap*.mp.

30 (physiotherap* or physical therap*).mp.

31 Speech-Language Pathology/ or Speech Therapy/ or speech patholog*.mp. or speech therap*.mp.

32 25 or 26 or 27 or 28 or 29 or 30 or 31

33 Meta-Analysis/ or "Systematic Review"/

34 (meta-analysis or meta-analyses).m_titl.

35 review.m_titl.

36 33 or 34 or 35

37 24 and 32 and 36

CINAHL – Systematic Reviews

1	(telemedicine or telehealth or telerehabilitation or telemetry or videoconferencing or telecommunications or remote consultation or telephone or email or internet or electronic mail or text messaging or cellular phones or (tele-rehabilitation or telerehab or telehealth or tele-health or telehomecare or tele-homecare or telecoaching or tele-coaching or telecommunication* or videoconference$ or video-conferenc* or videoconsultation or video-consultation or telestroke or teleconference* or tele-conference* or teleconsultation or tele-consultation or telecare or ehealth or e-health) or ((rehabilitation or therap* or treatment or communication or consultation) N5 (telephone* or phone* or video* or internet* or computer* or sensor* or modem or webcam or website* or email)) or ((cell* or smart* or mobile or android or internet or web) n3 (comput* or device or app* or phone)) or (smartphone or text-messag* or (tablet ADJ3 (device* or comput*))) or (mhealth or m-health or m health or mobile health))
2	(MH "Telemedicine+")
3	(MH "Telemedicine+")
4	(MH "Telemedicine+")
5	(MH "Telehealth")
6	(MH "Telerehabilitation")
7	(MH "telemetry")
8	(MH "videoconferencing")
9	(MH "Telecommunications")
10	(MH "Remote Consultation")
11	(MH "Telephone")
12	email
13	(MH "Internet")
14	(MH "Text Messaging")
15	(MH "Cellular Phone")
16	(tele-rehabilitation or telerehab or telehealth or tele-health or telehomecare or tele-homecare or telecoaching or tele-coaching or telecommunication* or videoconference$ or video-conferenc* or videoconsultation or video-consultation or telestroke or teleconference* or tele-conference* or teleconsultation or tele-consultation or telecare or ehealth or e-health)
17	((rehabilitation or therap* or treatment or communication or consultation) N5 (telephone* or phone* or video* or internet* or computer* or sensor* or modem or webcam or website* or email))
18	((cell* or smart* or mobile or android or internet or web) n3 (comput* or device or app* or phone))
19	(smartphone or text-messag* or (tablet ADJ3 (device* or comput*)))
20	(mhealth or m-health or m health or mobile health)
21	physiotherapy or physical therapy or physiotherapist or physical therapist
22	occupational therapy or occupational therapist or occupational therapists or ot
23	psychologist or psychology
24	neuropsychologist or neuropsychology
25	speech language pathology or speech pathology or speech therapy or language therapy or slp or speech language pathologist
26	podiatrist or podiatry
27	allied health professionals or allied healthcare professionals or allied health workers
28	21 OR 22 OR 23 OR 24 OR 25 OR 26 OR 27

29	TI (meta-analysis or systematic review or literature review or meta analysis or overview or review or meta-synthesis or meta synthesis)
30	(MH "Systematic Review")
31	29 OR 30
32	1 OR 2 OR 3 OR 4 OR 5 OR 6 OR 7 OR 8 OR 9 OR 10 OR 11 OR 12 OR 13 OR 14 OR 15 OR 16 OR 17 OR 18 OR 19 OR 20
33	28 AND 31 AND 32

Ovid MEDLINE(R) – Randomized Controlled Trials

1 telemedicine.mp.

2 exp Telemedicine/

3 exp Telemedicine/

4 telemetry.mp.

5 exp Videoconferencing/

6 telecommunications.mp.

7 remote consultation.mp.

8 remote sensing technology.mp

9 exp Telephone

10 electronic mail.mp

11 text messaging.mp.

12 computers.mp.

13 exp Cell Phone/

14 (tele-rehabilitation or telerehab or telehealth or tele-health or telehomecare or tele-homecare or telecoaching or tele-coaching or telecommunication* or videoconference$ or video-conferenc* or videoconsultation or video-consultation or telestroke or teleconference* or tele-conference* or teleconsultation or tele-consultation or telecare or ehealth or e-health).mp.

15 ((rehabilitation or therap* or treatment or communication or consultation) adj5 (telephone* or phone* or video* or internet* or computer* or sensor* or modem or webcam or website* or email)).mp

16 (remote* or distance* or distant).mp. adj5 ((rehabilitation or therap*).mp. or *interven*/ or treatment.mp. or physio*.mp. or physical therap*.mp. or occupational therap*.mp. or neuropsycholog*.mp. or psycholog*.mp. or speech therap*.mp. or speech patholog*.mp. or

communication.mp. or podiatr*.mp. or consultation.mp. or care.mp. or specialist*.mp. or monitor*.mp. or virtual reality.mp. or virtual environment*.mp. or technolog*.mp.)

17 (mhealth or m-health or m health or mobile health).mp.

18 (smartphone or text-messag* or (tablet adj3 (device* or comput*))).mp.

19 ((cell* or smart* or mobile or android or internet or web) adj3 (comput* or device or app* or phone)).mp.

20 randomi$ control$ trial.mp.

21 Randomized Controlled Trial/

22 Controlled clinical trial/

23 random*.ab.

24 20 or 21 or 22 or 23

25 1 or 2 or 3 or 4 or 5 or 6 or 7 or 8 or 9 or 10 or 11 or 12 or 13 or 14 or 15 or 16 or 17 or 18 or 19

26 occupational therapy/ or occupational therap*.mp.

27 allied health.mp.

28 (physiotherap* or physical therap*).mp.

29 speech therapy/ or speech therap*.mp.

30 podiatry/ or podiatr*.mp.

31 neuropsychology/ or neuropsycholog*.mp.

32 psychology/ or psycholog*.mp.

33 26 or 27 or 28 or 29 or 30 or 31 or 32

34 25 and 26 and 34

35 limit 34 to yr="2017 - 2023"

Cochrane Central Register of Controlled Trials CENTRAL – Randomized Controlled Trials

1 exp Telemedicine/

2 Telemetry/

3 exp Videoconferencing/

4 Telecommunications/

5 Remote Consultation/

6 Remote Sensing Technology/

7 exp Telephone/

8 Electronic Mail/

9 exp Internet/

10 Text Messaging/

11 Computers/

12 exp Microcomputers/

13 Minicomputers/

14 Cell Phone/

15 (telemedicine or telemetry or telerehabilitation or tele-rehabilitation or telerehab or telehealth or tele-health or telehomecare or tele-homecare or telecoaching or tele-coaching or telecommunication* or videoconference$ or video-conferenc* or videoconsultation or video-consultation or telestroke or teleconference* or tele-conference* or teleconsultation or tele-consultation or telecare or ehealth or e-health).mp.

16 (telespeech or tele-speech or teleOT or tele-OT or telepractice or teletherap*).mp.

17 ((rehabilitation or therap* or treatment or communication or consultation) adj5 (telephone* or phone* or video* or internet* or computer* or sensor* or modem or webcam or website* or email)).mp.

18 ((remote* or distance* or distant) adj5 (rehabilitation or therap* or treatment or physio* or physical therap* or occupational therap* or neuropsycholog* or psycholog* or speech therap* or speech patholog* communication or consultation or care or specialist* or monitor* or virtual reality or virtual environment* or technolog*)).mp.

19 ((cell* or smart* or mobile or android or internet or web) adj3 (comput* or device or app* or phone)).mp.

20 (smartphone or text-messag* or (tablet adj3 (device* or comput*))).mp.

21 (mhealth or m-health or m health or mobile health).mp.

22 (tele adj3 (game* or game* or exergame* or virtual reality*)).mp.

23 1 or 2 or 3 or 4 or 5 or 6 or 7 or 8 or 9 or 10 or 11 or 12 or 13 or 14 or 15 or 16 or 17 or 18 or 19 or 20 or 21 or 22

24 Random Allocation/

25 Controlled Clinical Trial/

26 Control Groups/

27 Randomized Controlled Trial/

28 24 or 25 or 26 or 27

29 allied health.mp.

30 Occupational Therapy/ or occupational therap*.mp.

31 (physiotherap* or physical therap*).mp. or Physical Therapy Modalities/

32 Speech Therapy/ or speech therap*.mp. or (speech adj 2 language therap*).mp. or speech patholog*.mp.

33 Psychology/ or psycholog*.mp.

34 neuropsycholog*.mp. or Neuropsychology/

35 Podiatry/ or podiatr*.mp.

36 29 or 30 or 31 or 32 or 33 or 34 or 35

37 23 and 28 and 37

38 limit 41 to yr="2017 - 2023"

EMBASE – Randomized Controlled Trials

1 exp Telemedicine/

2 telemetry.mp.

3 exp Videoconferencing/

4 telecommunications.mp.

5 remote consultation.mp.

6 remote sensing technology.mp.

7 exp Telephone/

8 electronic mail.mp.

9 text messaging.mp.

10 computers.mp.

11 exp Cell Phone/

12 (tele-rehabilitation or telerehab or telehealth or tele-health or telehomecare or tele-homecare or telecoaching or tele-coaching or telecommunication* or videoconference$ or video-conferenc* or videoconsultation or video-consultation or telestroke or teleconference* or tele-conference* or teleconsultation or tele-consultation or telecare or ehealth or e-health).mp.

13 ((rehabilitation or therap* or treatment or communication or consultation) adj5 (telephone* or phone* or video* or internet* or computer* or sensor* or modem or webcam or website* or email)).mp.

14 (remote* or distance* or distant).mp. adj5 ((rehabilitation or therap*).mp. or *interven*/ or treatment.mp. or physio*.mp. or physical therap*.mp. or occupational therap*.mp. or neuropsycholog*.mp. or psycholog*.mp. or dietetici*.mp. or speech therap*.mp. or speech patholog*.mp. or communication.mp. or social work*.mp. or podiatr*.mp. or consultation.mp. or care.mp. or specialist*.mp. or monitor*.mp. or virtual reality.mp. or virtual environment*.mp. or technolog*.mp.)

15 (mhealth or m-health or m health or mobile health).mp.

16 (smartphone or text-messag* or (tablet adj3 (device* or comput*))).mp.
((cell* or smart* or mobile or android or internet or web) adj3 (comput* or device or app* or phone)).mp.

17 Internet/

18 Microcomputers/

19 Minicomputers/

20 (telemedicine or telemetry or telerehabilitation or tele-rehabilitation or telerehab or telehealth or tele-health or telehomecare or tele-homecare or telecoaching or tele-coaching or telecommunication* or videoconference$ or video-conferenc* or videoconsultation or video-consultation or telestroke or teleconference* or tele-conference* or teleconsultation or tele-consultation or telecare or ehealth or e-health).mp.

21 (telespeech or tele-speech or teleOT or tele-OT or telepractice or teletherap*).mp.

22 (tele adj3 (game* or game* or exergame* or virtual reality*)).mp.

23 1 or 2 or 3 or 4 or 5 or 6 or 7 or 8 or 9 or 10 or 11 or 12 or 13 or 14 or 15 or 16 or 17 or 18 or 19 or 20 or 21 or 22 or 23

24 Randomized Controlled Trial/

25 Controlled Clinical Trial/

26 Random Allocation/

27 Control Groups/

28 25 or 26 or 27 or 28

30 allied health.mp.

31 Podiatry/ or podiatr*.mp.

32 neuropsycholog*.mp.

33 psycholog*.mp.

34 Occupational Therapy/ or occupational therap*.mp.

35 (physiotherap* or physical therap*).mp.

36 Speech-Language Pathology/ or Speech Therapy/ or speech patholog*.mp. or speech therap*.mp.

37 30 or 31 or 32 or 33 or 34 or 35 or 36

38 23 and 28 and 37

39 limit 38 to yr="2017 - 2023"

CINAHL – Randomized Controlled Trials

1	(MH "Telemedicine")
2	(MH "Telemetry")
3	(MH "Videoconferencing+")
4	(MH "Remote Consultation")
5	(MH "Telephone+")
6	(MH "Telecommunications")
7	(MH "Email")
8	(MH "Internet+")
9	(MH "Text Messaging")
10	(MH "Computers and Computerization")
11	(MH "Microcomputers+")
12	(MH "Minicomputers")
13	(MH "Cellular Phone+")
14	(telemedicine or telemetry or telerehabilitation or tele-rehabilitation or telerehab or telehealth or tele-health or telehomecare or tele-homecare or telecoaching or tele-coaching or telecommunication* or videoconference$ or video-conferenc* or videoconsultation or video-consultation or telestroke or teleconference* or tele-conference* or teleconsultation or tele-consultation or telecare or ehealth or e-health)
15	(telespeech or tele-speech or teleOT or tele-OT or telepractice or teletherap*)
16	(telespeech or tele-speech or teleOT or tele-OT or telepractice or teletherap*)
17	((rehabilitation or therap* or treatment or communication or consultation) N5 (telephone* or phone* or video* or internet* or computer* or sensor* or modem or webcam or website* or email))
18	((cell* or smart* or mobile or android or internet or web) n3 (comput* or device or app* or phone))
19	(smartphone or text-messag* or (tablet ADJ3 (device* or comput*)))
20	(mhealth or m-health or m health or mobile health)
21	(tele N3 (game* or game* or exergame* or virtual reality*))
22	(MH "Randomized Controlled Trials")
23	(MH "Control Group")

24 (MH "Random Assignment")
25 (MH "Clinical Trials")
26 allied health
27 physiotherapy or physical therapy or physiotherapist or physical therapist
28 occupational therapy or occupational therapist or occupational therapists or ot
29 psychologist or psychology
30 neuropsychologist or neuropsychology
31 speech language pathology or speech pathology or speech therapy or language therapy or slp or speech language pathologist
32 podiatrist or podiatry
33 allied health professionals or allied healthcare professionals or allied health workers
34 S21 OR S22 OR S23 OR S24 OR S25 OR S26 OR S27
35 Limit 34 to yr="2017 – 2023"

Supplementary Table S2. Allied Health Dictionary for QDA Miner

ALLIEDHEALTH	@AH1 [ALLIED_HEALTH BEFORE THERAP /A /S5] (1)<br>@AH2 [A_L_L_I_E_D_H_E_A_L_T_H BEFORE T_H_E_R_A_P /A /S5] (1)
NEURO	N_E_U_R_O (1)<br>NEURO (1)
OCCUPATIONAL	@OT1 [VOCATIONAL BEFORE THERAP /A /S3] (1)<br>@OT2 [VOCATIONAL BEFORE PROGRAM /A /S3] (1)<br>@OT3 [VOCATIONAL BEFORE REHAB /A /S3] (1)<br>O_C_C_U_P_A_T_I_O_N_A_L_T_H_E_R (1)<br>OCCUPATIONAL_THERAP (1)<br>V_O_C_A_T_I_O_N_A_L_R_E_H_A_B (1)<br>VOCATIONAL_REHAB (1)<br>VOCATIONAL_THERAP (1)<br>V_O_C_A_T_I_O_N_A_L_T_H_E_R_A_P (1)
PHYSIOTHERAPY	PHYSICAL_THERAP (1)<br>P_H_Y_S_I_C_A_L_T_H_E_R_A_P (1)<br>P_H_Y_S_I_O (1)<br>PHYSIO (1)<br>PHYSIOTHERAPIST (1)<br>PHYSIOTHERAPY (1)
PSYCHOLOGY	@PSYCH1 [MENTAL_HEALTH BEFORE THERAP /A /S2] (1)<br>@PSYCH2 [COGNIT BEFORE THERAP /A /S4] (1)<br>@PSYCH3 [BEHAV BEFORE THERAP /A /S2] (1)<br>P_S_Y_C_H_O_L (1)<br>PSYCHOL (1)
RCT	@RCT1 [RANDOM AND CONTROL AND TRIAL /A /S/D] (1)<br>@RCT2 [RANDOM AND CLINICAL AND TRIAL /A /S/D] (1)

	@RCT3 [RANDOM BEFORE CONTROL BEFORE STUDY /A /S5/D5] (1)<br>@RCT4 [RANDOM BEFORE CLINICAL BEFORE STUDY /A /S5/D5] (1)<br>R_C_T (1)<br>RCT (1)
SPEECH	@SPEECH1 [SPEECH BEFORE THERAP /A /S3] (1)<br>@SPEECH2 [SPEECH BEFORE PATHOLOG /A /S3] (1)<br>@SPEECH3 [LANGUAGE AND THERAP /A /S] (1)
TELEHEALTH	@TELE1 [VIDEO BEFORE CONFERENCE /A /S2] (1)<br>@TELE2 [MOBILE BEFORE HEALTH /A /S2] (1)<br>@TELE3 [TELEPHONE BEFORE CONFERENCE /A /S2] (1)<br>EHEALTH (1)<br>MHEALTH (1)<br>T_E_L_E_H_E_A_L_T_H (1)<br>T_E_L_E_P_H_O_N_E (1)<br>TELEHEALTH (1)<br>TELEPHONE (1)

Supplementary Table S3 – Excluded Full Texts

Author	Reason for exclusion
(Acierno, Gros et al. 2016)	Did not include our specified allied health
(Adams, Ellington et al. 2023)	Not comparable interventions
(Adewuya, Momodu et al. 2019)	Not comparable interventions
(Aily, Castilho de Almeida et al. 2020)	Abstract only
(Alegría, Ludman et al. 2014)	Intervention not telehealth vs FTF
(Alp, Mengi et al. 2014)	Not comparable interventions
(Aneshensel, Frerichs et al. 1982)	Assessment only or single session only
(Appel, Bleiberg and Noiseux 2002)	Not comparable interventions
(Arthur, Smith et al. 2002)	Did not include our specified allied health
(Avila, Claes et al. 2018)	Inadequate detail on comparator / intervention
(Ayan, Kara and Erbayraktar 2023)	Abstract only
(Azma, RezaSoltani et al. 2018)	Not comparable interventions

(Bahadori, Sami et al. 2023)	Did not include our specified allied health
(Barcelo-Soler, Banos et al. 2019)	Intervention not telehealth vs FTF
(Barnason, Zimmerman et al. 2009)	Did not include our specified allied health
(Baron, Corden et al. 2011)	Intervention not telehealth vs FTF
(Barton, Pazzinatto et al. 2022)	Abstract only
(Bravo-Escobar, Gonzalez-Represas et al. 2021)	Not comparable interventions
(Bell, Temkin et al. 2005)	Intervention not telehealth vs FTF
(Bell, Hoffman et al. 2008)	Not comparable interventions
(Bell, Brockway et al. 2011)	Not comparable interventions
(Bell, Fann et al. 2017)	Not comparable interventions
(Bennell, Campbell et al. 2017)	Not comparable interventions
(Bergquist, Gehl et al. 2009)	Intervention not telehealth vs FTF
(Bettger, Green et al. 2020)	Inadequate detail on comparator / intervention
(Blanca, Raquel and Antonio 2020)	Abstract only
(Bombardier, Bell et al. 2009)	Intervention not telehealth vs FTF
(Bonato, Turrini et al. 2020)	Did not include our specified allied health
(Borgen, Løvstad et al. 2023)	Not comparable interventions
(Bossen, Veenhof et al. 2014)	Not comparable interventions
(Boter 2004)	Did not include our specified allied health
(Bouchard, Paquin et al. 2004)	Intervention not telehealth vs FTF
(Bravo-Escobar, Gonzalez-Represas et al. 2021)	Intervention not telehealth vs FTF
(Brouwers, van der Poort et al. 2021)	Not comparable interventions
(Bulguroglu and Bulguroglu 2023)	Intervention not telehealth vs FTF
(Bury and Stokes 2020)	Not RCT
(Campbell, Robertson et al. 1999)	Intervention not telehealth vs FTF

(Carey, Durfee et al. 2007)	Intervention not telehealth vs FTF
(Celano, Gomez-Bernal et al. 2020)	Intervention not telehealth vs FTF
(Chen, Lin et al. 2021)	Not comparable interventions
(Chen, Lin et al. 2020)	Not RCT
(Chien, Lee et al. 2011)	Not comparable interventions
(Choi, Marti et al. 2013)	Did not include our specified allied health
(Choi, Marti et al. 2014)	Did not include our specified allied health
(Choi, Marti and Conwell 2016)	Did not include our specified allied health
(Chumbler Neale, Quigley et al. 2012)	Intervention not telehealth vs FTF
(Chumbler, Li et al. 2015)	Intervention not telehealth vs FTF
(Chun, Carson et al. 2020)	Did not include our specified allied health
(Church and Clond 2019)	Not RCT
(Cil, Serif et al. 2023)	Not comparable interventions
(Claes, Cornelissen et al. 2020)	Intervention not telehealth vs FTF
(Collins, Burns et al. 2017)	Not RCT
(Compen, Bisseling et al. 2018)	Abstract only
(Compen, Adang et al. 2020)	Did not include our specified allied health
(Corcoran, Hui and Woo 2003)	Not RCT
(Correia, Nogueira et al. 2019)	Not RCT
(Cosio, Jin et al. 2011)	Intervention not telehealth vs FTF
(Crow, Mitchell et al. 2009)	Outcomes - not relevant or not reported
(Cui, Janela et al. 2023)	Not comparable interventions
(Czaja, Loewenstein et al. 2013)	Intervention not telehealth vs FTF
(Dahmen, Gao et al. 2022)	Not published in English
(Dalal, Evans et al. 2007)	Did not include our specified allied health
(Dallolio, Menarini et al. 2008)	Not comparable interventions

(Davis, Burgio et al. 2004)	Outcomes - not relevant or not reported
(De Las Heras, Balbino et al. 2020)	Not comparable interventions
(De Luca, Aragona et al. 2018)	Intervention not telehealth vs FTF
(De Luca, Russo et al. 2021)	Did not include our specified allied health
(de Toledo, Jiménez et al. 2006)	Did not include our specified allied health
(Dear, Zou et al. 2015)	Not comparable interventions
(Diokno, Newman et al. 2018)	Intervention not telehealth vs FTF
(Dixon, Hollinghurst et al. 2016)	Did not include our specified allied health
(Donkers, Nickel et al. 2020)	Not comparable interventions
(Dorsey, Deuel et al. 2010)	Did not include our specified allied health
(Dorstyn, Mathias et al. 2012)	Intervention not telehealth vs FTF
(Doze, Simpson et al. 1999)	Not RCT
(Drozd, Skeie et al. 2014)	Intervention not telehealth vs FTF
(Durst, Roesel et al. 2020)	Intervention not telehealth vs FTF
(Dwight-Johnson, Aisenberg et al. 2011)	Not comparable interventions
(Egede, Acierno et al. 2016)	Did not include our specified allied health
(Egede, Gebregziabher et al. 2017)	Outcomes - not relevant or not reported
(Egede, Walker et al. 2018)	Outcomes - not relevant or not reported
(Egner, Phillips et al. 2003)	Did not include our specified allied health
(Emmerson, Harding and Taylor 2017)	Intervention not telehealth vs FTF
(Eriksson, Lindström et al. 2009)	Not RCT
(Everitt, Landau et al. 2019)	Not comparable interventions

(Fanuscu, Öz and Ulger 2023)	Abstract only
(Fernandez, Bergado Rosado et al. 2017)	Intervention not telehealth vs FTF
(Finlayson, Preissner et al. 2011)	Intervention not telehealth vs FTF
(Flynn, Preston et al. 2020)	Abstract only
(Forducey, Glueckauf et al. 2012)	Intervention not telehealth vs FTF
(Fortney, Pyne et al. 2007)	Did not include our specified allied health
(Franklin, Cuccurullo et al. 2017)	Not comparable interventions
(Frederix, Solmi et al. 2017)	Did not include our specified allied health
(Frederix, Van Driessche et al. 2015)	Intervention not telehealth vs FTF
(Frueh, Monnier et al. 2007)	Did not include our specified allied health
(Garcia-Palacios, Herrero et al. 2015)	Not comparable interventions
(Giallauria, Lucci et al. 2006)	Assessment only or single session only
(Gibson, Coulson et al. 2011)	Intervention not telehealth vs FTF
(Ginis, Nieuwboer et al. 2016)	Not comparable interventions
(Glueckauf, Davis et al. 2012)	Intervention not telehealth vs FTF
(Gohir, Eek et al. 2021)	Protocol
(Gordon, English et al. 2002)	Did not include our specified allied health
(Greene, Morland et al. 2010)	Outcomes - not relevant or not reported
(Gros, Yoder et al. 2011)	Not RCT
(Gros, Lancaster et al. 2018)	Did not include our specified allied health
(Grubbs, Fortney et al. 2015)	Outcomes - not relevant or not reported
(Grzincich, Gagliardini et al. 2010)	Assessment only or single session only
(Gustafsson 2020)	Not RCT
(Gutiérrez, Galán Del Río et al. 2013)	Not RCT

(Hagen and Grotle 2017).	Intervention not telehealth vs FTF
(Hagovská, Dzvoník and Olekszyová 2017)	Intervention not telehealth vs FTF
(Hall, Lattie et al. 2017).	Did not include our specified allied health
(Hanssen, Nordrehaug et al. 2007)	Did not include our specified allied health
(Hanssen, Nordrehaug et al. 2009)	Did not include our specified allied health
(Hassall, Wootton and Guilfoyle 2003)	Assessment only or single session only
(Heckman, Heckman et al. 2013)	Intervention not telehealth vs FTF
(Heckman, Heckman et al. 2017)	Not comparable interventions
(Heckman, Markowitz et al. 2018)	Not comparable interventions
(Hedman, Andersson et al. 2011)	Not comparable interventions
(Hegel, Lyons et al. 2011)	Outcomes - not relevant or not reported
(Hernandez-Tejada, Zoller et al. 2014)	Outcomes - not relevant or not reported
(Hernando-Requejo, Huertas-González et al.)	Did not include our specified allied health
(Horton, Marland et al. 2021)	Not RCT
(Hou, Yang et al. 2019).	Intervention not telehealth vs FTF
(Hurley, Vasavada et al. 2022)	Abstract only
(Iles, Taylor et al. 2011)	Not comparable interventions
(Jaconis, Santa Ana et al. 2017)	Not RCT
(Jansen-Kosterink, Huis in ’t Veld et al. 2015)	Not RCT
(Jansons, Robins et al. 2017)	Did not include our specified allied health
(Jarbandhan, Toelsie et al. 2022)	Not comparable interventions
(Jarvela-Reijonen, Karhunen et al. 2018)	Not comparable interventions

(Jelinek, Vale et al. 2009)	Did not include our specified allied health
(Jiang, Koh et al. 2021)	Did not include our specified allied health
(Jódar-Sánchez, Ortega et al. 2014)	Did not include our specified allied health
(Jolly, Lip et al. 2003)	Did not include our specified allied health
(Jolly, Taylor et al. 2007)	Did not include our specified allied health
(Jolly, Lip et al. 2009)	Did not include our specified allied health
(Jung, Park et al. 2023)	Did not include our specified allied health
(Kalapatapu, Ho et al. 2014)	Outcomes - not relevant or not reported
(Kaldo, Levin et al. 2008)	Did not include our specified allied health
(Kallestad, Scott et al. 2021)	Not comparable interventions
(Kalron, Tawil et al. 2018)	Intervention not telehealth vs FTF
(Kalron, Tawil et al. 2018)	Not comparable interventions
(Kasnakova, Mihaylova et al. 2022)	Inadequate detail on comparator / intervention
(Kenny, Gilmartin and Thompson 2022)	Not comparable interventions
(Keskin, Gurcan Atci et al. 2020)	Intervention not telehealth vs FTF
(Khalil, Busse et al. 2017)	Intervention not telehealth vs FTF
(Kidholm, Rasmussen et al. 2016)	Outcomes - not relevant or not reported
(Kim, Jhoo and Jang 2017)	Not RCT
(Kirkness, Cain et al. 2017)	Inadequate detail on comparator / intervention
(Kizony, Weiss et al. 2013)	Assessment only or single session only
(Klee, Stacy et al. 2016)	Outcomes - not relevant or not reported
(Kloek, Bossen et al. 2018)	Not comparable interventions
(Kloek, van Dongen et al. 2018)	Not comparable interventions
(Kroenke, Krebs et al. 2014)	Did not include our specified allied health
(Krpič, Savanović and Cikajlo 2013)	Not comparable interventions

(Kryger, Crytzer et al. 2019)	Not comparable interventions
(Kuster, Dalsbø et al. 2017)	Intervention not telehealth vs FTF
(Lee, Hur et al. 2013)	Not comparable interventions
(Li, Hung et al. 2022)	Intervention not telehealth vs FTF
(Li, Zhao et al. 2023)	Did not include our specified allied health
(Lightstone, Bailey and Voros 2015)	Did not include our specified allied health
(Linder, Rosenfeldt et al. 2015)	Not comparable interventions
(Lindsay, Kauth et al. 2015)	Not RCT
(LoSavio, Worley et al. 2023)	Not RCT
(Lovell, Fullalove et al. 2000)	Intervention not telehealth vs FTF
(Luangapichart, Saisavoey and Viravan 2022)	Not comparable interventions
(Luxton, Pruitt et al. 2015)	Intervention not telehealth vs FTF
(Maddison, Pfaeffli et al. 2015)	Intervention not telehealth vs FTF
(Maisiak, Austin and Heck 1996)	Intervention not telehealth vs FTF
(Mallet, Shamloul et al. 2023)	Intervention not telehealth vs FTF
(Maltais, Bourbeau et al. 2008)	Not comparable interventions
(Man, Soong et al. 2006)	Intervention not telehealth vs FTF
(Maresca, Maggio et al. 2019)	Inadequate detail on comparator / intervention
(Marino, Chilà et al. 2020)	Not comparable interventions
(Marziali 2009)	Not comparable interventions
(Marziali and Donahue 2006)	Intervention not telehealth vs FTF
(Mashima, Birkmire-Peters et al. 2003)	Co-located telehealth
(Mayo, Nadeau et al. 2008)	Not comparable interventions
(Mayor-Silva, Romero-Saldaña et al. 2021)	Assessment only or single session only
(McBeth, Prescott et al. 2012)	Not comparable interventions
(Meichsner, Töpfer et al. 2019)	Not comparable interventions

(Meng, Marino et al. 2021)	Did not include our specified allied health
(Messinis, Nasios et al. 2017)	Intervention not telehealth vs FTF
(Miller, Haskell et al. 1984)	Did not include our specified allied health
(Mohr, Carmody et al. 2011)	Not comparable interventions
(Mohr, Hart and Vella 2007)	Intervention not telehealth vs FTF
(Mohr, Likosky et al. 2000)	Not comparable interventions
(Mojica, Vasavada et al. 2023)	Inadequate detail on comparator / intervention
(Morland, Raab et al. 2013)	Outcomes - not relevant or not reported
(Mottaghi, Rahimian Boogar et al. 2022)	Not published in English
(Munro, Shaw et al. 1994)	Not comparable interventions
(Nambi, Alghadier et al. 2023)	Intervention not telehealth vs FTF
(Naylor, Keefe et al. 2008)	Intervention not telehealth vs FTF
(Nelson, Bourke et al. 2020)	Did not include our specified allied health
(Neubeck, Freedman et al. 2011)	Intervention not telehealth vs FTF
(North 2020)	Not RCT
(Novella, Ng and Samuolis 2022)	Did not include our specified allied health
(Oerkild, Frederiksen et al. 2011)	Not comparable interventions
(Ofoegbu, Asogwa et al. 2020)	Not comparable interventions
(Oka, De Marco et al. 2000)	Not comparable interventions
(Onan and Ulger 2022)	Abstract only
(Ora, Kirmess et al. 2020)	Not comparable interventions
(Ortiz-Gutiérrez, Cano-de-la-Cuerda et al. 2013)	Not comparable interventions
(Ownsworth, Chambers et al. 2023)	Not comparable interventions
(Padraig, Sandra et al. 2016)	Did not include our specified allied health
(Pak, Janela et al. 2023)	Not comparable interventions

(París, Saleta et al. 2011)	Not comparable interventions
(Pastora-Bernal, Martin-Valero and Baron-Lopez 2018)	Not comparable interventions
(Pastora-Bernal, Martin-Valero et al. 2018)	Not comparable interventions
(Patel, Ojo et al. 2017)	Not comparable interventions
(Paul, Renfrew et al. 2019)	Intervention not telehealth vs FTF
(Phillips, Vesmarovich et al. 2001)	Not comparable interventions
(Pidgeon 2017)	Not RCT
(Pignato, Arbeeva et al. 2018)	Not comparable interventions
(Piotrowicz, Baranowski et al. 2010)	Not comparable interventions
(Piotrowicz, Mierzyńska et al. 2022)	Not comparable interventions
(Piron, Turolla et al. 2009)	Did not include our specified allied health
(Poon, Hui et al. 2005)	Inadequate detail on comparator / intervention
(Poulin, Korner-Bitensky et al. 2017)	Intervention not telehealth vs FTF
(Price and Gros 2014)	Outcomes - not relevant or not reported
(Pyne, Fortney et al. 2010)	Outcomes - not relevant or not reported
(Raina, Morse et al. 2022)	Not comparable interventions
(Rakhshan, Khoshnood et al. 2022)	Intervention not telehealth vs FTF
(Ransom, Heckman et al. 2008)	Intervention not telehealth vs FTF
(Redfern, Briffa et al. 2008)	Not comparable interventions
(Redfern, Briffa et al. 2009)	Not comparable interventions
(Redzuan, Engkasan et al. 2012)	Not comparable interventions
(Reuter, Mehnert et al. 2012)	Intervention not telehealth vs FTF
(Russell, Buttrum et al. 2003, Riegler,	Not RCT

Neils-Strunjas et al. 2013)	
(Russell, Buttrum et al. 2003)	Co-located telehealth
(Russell, Buttrum et al. 2011)	Co-located telehealth
(Salazar, Warden et al. 2000)	Did not include our specified allied health
(Salvetti, Oliveira et al. 2008)	Intervention not telehealth vs FTF
(Sankaran, Dendale and Coninx 2019)	Intervention not telehealth vs FTF
(Sari, Oskay and Tufan 2022)	Abstract only
(Scheenen, Visser-Keizer et al. 2017)	Not comparable interventions
(Scherr, Kastner et al. 2009)	Did not include our specified allied health
Schultz 2009	Intervention not telehealth vs FTF
(Seto, Leonard et al. 2012)	Did not include our specified allied health
(Severe, Pfeiffer et al. 2022)	Not comparable interventions
(Shahidi, Zargar et al. 2023)	Not comparable interventions
(Shetty, Ku and Chippala 2022)	Intervention not telehealth vs FTF
(Smith, Arthur et al. 2004)	Did not include our specified allied health
(Smith, McKelvie et al. 2011)	Did not include our specified allied health
(Smith, Egbert et al. 2012)	Intervention not telehealth vs FTF
(Smith, Faux et al. 2019)	Intervention not telehealth vs FTF
(Stefan 2013)	Assessment only or single session only
(Stein, Herman et al. 2007)	Not comparable interventions
(Strachan, Gros et al. 2012)	Did not include our specified allied health
(Streater, Spector et al. 2017)	Not comparable interventions
(Stubbings, Rees et al. 2013)	Did not include our specified allied health
(Stuifbergen, Becker et al. 2012)	Did not include our specified allied health
(Tan, Teo et al. 2013)	Not RCT
(Taylor, Watt et al. 2007)	Intervention not telehealth vs FTF
(Taylor, Peterson et al. 2017)	Not comparable interventions

(Torpil and Kaya 2022)	Inadequate detail on comparator / intervention
(Torrisi, Maresca et al. 2019)	Intervention not telehealth vs FTF
(Tousignant, Boissy et al. 2006)	Not RCT
(Tousignant, Moffet et al. 2011)	Not comparable interventions
(Tousignant, Moffet et al. 2015)	Outcomes - not relevant or not reported
(Tuerk, Yoder et al. 2010)	Not RCT
(Türkmen, Analay Akbaba and Altun 2020)	Intervention not telehealth vs FTF
(Turolla, Rossettini et al. 2020)	Not RCT
(Vale, Jelinek et al. 2003)	Not comparable interventions
(Vallejo, Ortega et al. 2015)	Not comparable interventions
(van den Berg, Crotty et al. 2016)	Did not include our specified allied health
(van der Linden, Sitskoorn et al. 2018)	Intervention not telehealth vs FTF
(van Vugt, Heymans et al. 2020)	Not comparable interventions
(Varnfield, Karunanithi et al. 2014)	Not comparable interventions
(Vasconcellos, Silva et al. 2023)	Not comparable interventions
(Vauth 2008)	Not published in English
(Vazquez, Lopez et al. 2020)	Not comparable interventions
(Venter, Burns et al. 2012)	Did not include our specified allied health
(Villatoro-Luque, Rodriguez-Almagro et al. 2023)	Not comparable interventions
(Vitacca, Bianchi et al. 2009)	Did not include our specified allied health
(Vogel, Launes et al. 2012)	Intervention not telehealth vs FTF
(Vuletic, Bell et al. 2016)	Not comparable interventions
(Wakasa, Odashima et al. 2020)	Intervention not telehealth vs FTF
(Wang, Guo et al. 2022)	Did not include our specified allied health
(Shanshan, Sze Ki Cheung et al. 2021)	Did not include our specified allied health

(Wangnamthip, Tip-apakoon et al. 2023)	Did not include our specified allied health
(Whealin, Seibert-Hatalsky et al. 2015)	Outcomes - not relevant or not reported
(Wilkinson, Spindler et al. 2016)	Did not include our specified allied health
(Wilkinson, Duncan-Skingle et al. 2008)	Not comparable interventions
(Wolf, Sahu et al. 2015)	Intervention not telehealth vs FTF
(Wu, Lin et al. 2006)	Intervention not telehealth vs FTF
(Wu, Keyes et al. 2010)	Did not include our specified allied health
(Yavas, Kahraman et al. 2023)	Intervention not telehealth vs FTF
(Yilmaz Yelvar, Çırak et al. 2017)	Intervention not telehealth vs FTF
(Ying, Ji et al. 2023)	Not comparable interventions
(Young, Chen et al. 2023)	Did not include our specified allied health
(Yuen, Gros et al. 2015)	Did not include our specified allied health
(Ziemba, Bradley et al. 2014)	Inadequate detail on comparator / intervention
(Zwisler, Soja et al. 2008)	Not comparable interventions

FTF-Face-to-Face

References for Excluded Papers:

Acierno, R., D. F. Gros, K. J. Ruggiero, B. M. Hernandez-Tejada, R. G. Knapp, C. W. Lejuez, W. Muzzy, C. B. Frueh, L. E. Egede and P. W. Tuerk (2016). "Behavioral activation and therapeutic exposure for posttraumatic stress disorder: a noninferiority trial of treatment delivered in person versus home-based telehealth." Depress Anxiety **33**(5): 415-423.

Adams, R. J., A. L. Ellington, K. A. Kuccera, H. Leaman, C. Smithson and J. T. Patrie (2023). "Telehealth-Guided Virtual Reality for Recovery of Upper Extremity Function Following Stroke." OTJR (Thorofare N J) **43**(3): 446-456.

Adewuya, A. O., O. Momodu, O. Olibamoyo, A. Adegbaju, O. Adesoji and A. Adegbokun (2019). "The effectiveness and acceptability of mobile telephone adherence support for management of depression in the Mental Health in Primary Care (MeHPriC) project, Lagos, Nigeria: A pilot cluster randomised controlled trial." J Affect Disord **253**: 118-125.

Aily, J., A. Castilho de Almeida, G. da Silva Ribeiro, M. de Noronha and S. Mattiello (2020). "Is a periodized circuit training delivered by telerehabilitation effective for patients with knee osteoarthritis? a phase i randomized controlled trial." Osteoarthritis and Cartilage **28**(Supplement 1): S468-S469.

Alegría, M., E. Ludman, E. N. Kafali, S. Lapatin, D. Vila, P. E. Shrout, K. Keefe, B. Cook, A. Ault, X. Li, A. M. Bauer, C. Epelbaum, C. Alcantara, T. I. Pineda, G. G. Tejera, G. Suau, K. Leon, A. S. Lessios, R. R. Ramirez and G. Canino (2014). "Effectiveness of the Engagement and Counseling for Latinos (ECLA) intervention in low-income Latinos." Med Care **52**(11): 989-997.

Alp, A., G. Mengi, A. Avsaroglu, M. Mert and D. Sigirli (2014). "Efficacy of Core-Stabilization Exercise and Its Comparison with Home-Based Conventional Exercise in Low Back Pain Patients." Türkiye Fiziksel Tip ve Rehabilitasyon Dergisi **60**.

Aneshensel, C. S., R. R. Frerichs, V. A. Clark and P. A. Yokopenic (1982). "Measuring Depression in the Community: A Comparison of Telephone and Personal Interviews." The Public Opinion Quarterly **46**(1): 110-121.
Appel, P. R., J. Bleiberg and J. Noiseux (2002). "Self-regulation training for chronic pain: can it be done effectively by telemedicine?" Telemed J E Health **8**(4): 361-368.
Arthur, H. M., K. M. Smith, J. Kodis and R. McKelvie (2002). "A controlled trial of hospital versus home-based exercise in cardiac patients." Medicine and science in sports and exercise **34**(10): 1544-1550.
Avila, A., J. Claes, K. Goetschalckx, R. Buys, M. Azzawi, L. Vanhees and V. Cornelissen (2018). "Home-Based Rehabilitation With Telemonitoring Guidance for Patients With Coronary Artery Disease (Short-Term Results of the TRiCH Study): Randomized Controlled Trial." Journal of medical Internet research **20**(6): e225.
Ayan, H., B. Kara and R. Erbayraktar (2023). "The effects of tele-rehabilitation-based stabilization exercises on balance, gait, functionality, pain and depression in individuals with chronic idiopathic neck pain." Annals of the Rheumatic Diseases **82**(Supplement 1): 2085-2086.
Azma, K., Z. RezaSoltani, F. Rezaeimoghaddam, A. Dadarkhah and S. Mohsenolhosseini (2018). "Efficacy of tele-rehabilitation compared with office-based physical therapy in patients with knee osteoarthritis: A randomized clinical trial." Journal of telemedicine and telecare **24**(8): 560-565.
Bahadori, M., R. Sami, S. Abolhassani and V. Atashi (2023). "Effect of a Mobile Training Application on Psychological Distress of Family Caregivers of Patients With Chronic Obstructive Pulmonary Disease." Archives of Rehabilitation **24**(2): 196-211.
Barcelo-Soler, A., R. M. Banos, Y. Lopez-Del-Hoyo, F. Mayoral, M. Gili, A. Garcia-Palacios, J. Garcia-Campayo and C. Botella (2019). "Interventions of computerized psychotherapies for depression in Primary Care in Spain." Actas Espanolas de Psiquiatria **47**(6): 236-246.
Barnason, S., L. Zimmerman, J. Nieveen, P. Schulz, C. Miller, M. Hertzog and C. Tu (2009). "Influence of a symptom management telehealth intervention on older adults' early recovery outcomes after coronary artery bypass surgery." Heart Lung **38**(5): 364-376.
Baron, K. G., M. Corden, L. Jin and D. C. Mohr (2011). "Impact of psychotherapy on insomnia symptoms in patients with depression and multiple sclerosis." J Behav Med **34**(2): 92-101.
Barton, C., M. Pazzinatto, Z. Perraton, K. Crossley, K. Dundules, T. Russell, D. De Oliveira Silva, P. O'Halloran and J. Kemp (2022). "Telehealth-delivered group-based education and exercise-therapy for knee osteoarthritis: A randomised clinical trial interrupted by the COVID-19 pandemic." Journal of Science and Medicine in Sport **25**(Supplement 2): S70-S71.
Bell, K. R., J. A. Brockway, T. Hart, J. Whyte, M. Sherer, R. T. Fraser, N. R. Temkin and S. S. Dikmen (2011). "Scheduled telephone intervention for traumatic brain injury: a multicenter randomized controlled trial." Arch Phys Med Rehabil **92**(10): 1552-1560.
Bell, K. R., J. R. Fann, J. A. Brockway, W. R. Cole, N. E. Bush, S. Dikmen, T. Hart, A. J. Lang, G. Grant, G. Gahm, M. A. Reger, J. St De Lore, J. Machamer, K. Ernstrom, R. Raman, S. Jain, M. B. Stein and N. Temkin (2017). "Telephone Problem Solving for Service Members with Mild Traumatic Brain Injury: A Randomized, Clinical Trial." Journal of Neurotrauma **34**(2): 313-321.
Bell, K. R., J. M. Hoffman, N. R. Temkin, J. M. Powell, R. T. Fraser, P. C. Esselman, J. K. Barber and S. Dikmen (2008). "The effect of telephone counselling on reducing post-traumatic symptoms after mild traumatic brain injury: A randomised trial." Journal of Neurology, Neurosurgery & Psychiatry **79**(11): 1275.
Bell, K. R., N. R. Temkin, P. C. Esselman, J. N. Doctor, C. H. Bombardier, R. T. Fraser, J. M. Hoffman, J. M. Powell and S. Dikmen (2005). "The effect of a scheduled telephone intervention on outcome after moderate to severe traumatic brain injury: a randomized trial." Arch Phys Med Rehabil **86**(5): 851-856.
Bennell, K. L., P. K. Campbell, T. Egerton, B. Metcalf, J. Kasza, A. Forbes, C. Bills, J. Gale, A. Harris, G. S. Kolt, S. J. Bunker, D. J. Hunter, C. A. Brand and R. S. Hinman (2017). "Telephone

Coaching to Enhance a Home-Based Physical Activity Program for Knee Osteoarthritis: A Randomized Clinical Trial." Arthritis Care and Research **69**(1): 84-94.
Bergquist, T., C. Gehl, J. Mandrekar, S. Lepore, S. Hanna, A. Osten and W. Beaulieu (2009). "The effect of internet-based cognitive rehabilitation in persons with memory impairments after severe traumatic brain injury." Brain Injury **23**(10): 790-799.
Bettger, J. P., C. L. Green, D. N. Holmes, A. Chokshi, R. C. Mather Iii, B. T. Hoch, A. J. de Leon, F. Aluisio, T. M. Seyler, D. J. Del Gaizo, J. Chiavetta, L. Webb, V. Miller, J. M. Smith, E. D. Peterson, J. Prvu Bettger and R. C. Mather, 3rd (2020). "Effects of Virtual Exercise Rehabilitation In-Home Therapy Compared with Traditional Care After Total Knee Arthroplasty: VERITAS, a Randomized Controlled Trial." Journal of Bone & Joint Surgery, American Volume **102**(2): 101-109.
Blanca, M. M., E. B. Raquel and G. C. J. Antonio (2020). "Face to face vs. online cognitive stimulation for people with cognitive impairment. A controlled trial." International Psychogeriatrics **32**(SUPPL 1): 175.
Bombardier, C. H., K. R. Bell, N. R. Temkin, J. R. Fann, J. Hoffman and S. Dikmen (2009). "The efficacy of a scheduled telephone intervention for ameliorating depressive symptoms during the first year after traumatic brain injury." J Head Trauma Rehabil **24**(4): 230-238.
Bonato, M., F. Turrini, V. De Zan, A. Meloni, M. Plebani, E. Brambilla, A. Giordani, C. Vitobello, R. Caccia, M. F. Piacentini, A. La Torre, A. Lazzarin, G. Merati, L. Galli and P. Cinque (2020). "A Mobile Application for Exercise Intervention in People Living with HIV." Medicine & Science in Sports & Exercise **52**(2): 425-433.
Borgen, I. M. H., M. Løvstad, S. L. Hauger, M. V. Forslund, I. Kleffelgård, N. Andelic, U. Sveen, H. L. Søberg, S. Sigurdardottir, L. Winter, M. Ø. Lindstad, C. Brunborg and C. Røe (2023). "Effect of an Individually Tailored and Home-Based Intervention in the Chronic Phase of Traumatic Brain Injury: A Randomized Clinical Trial." JAMA Network Open **6**(5): e2310821-e2310821.
Bossen, D., C. Veenhof, J. Dekker and D. de Bakker (2014). "The effectiveness of self-guided web-based physical activity interventions among patients with a chronic disease: a systematic review." Journal of Physical Activity & Health **11**(3): 665-677.
Boter, H. (2004). "Multicenter randomized controlled trial of an outreach nursing support program for recently discharged stroke patients." Stroke **35**(12): 2867-2872.
Bouchard, S., B. Paquin, R. Payeur, M. Allard, V. Rivard, T. Fournier, P. Renaud and J. Lapierre (2004). "Delivering cognitive-behavior therapy for panic disorder with agoraphobia in videoconference." Telemedicine Journal & E-Health **10**(1): 13-25.
Bravo-Escobar, R., A. Gonzalez-Represas, A. M. Gomez-Gonzalez and A. Heredia-Torres (2021). "Effectiveness of e-Health cardiac rehabilitation program on quality of life associated with symptoms of anxiety and depression in moderate-risk patients." Scientific reports **11**(1): 3760.
Brouwers, R. W. M., E. K. J. van der Poort, H. M. C. Kemps, M. E. van den Akker-van Marle and J. J. Kraal (2021). "Cost-effectiveness of Cardiac Telerehabilitation With Relapse Prevention for the Treatment of Patients With Coronary Artery Disease in the Netherlands." JAMA network open **4**(12): e2136652.
Bulguroglu, H. I. and M. Bulguroglu (2023). "The effects of online pilates and face-to-face pilates in healthy individuals during the COVID-19 pandemic: a randomized controlled study." BMC sports science, medicine & rehabilitation **15**(1): 12.
Bury, T. J. and E. K. Stokes (2020). "On "Musculoskeletal Physical Therapy During the COVID-19 Pandemic: Is Telerehabilitation the Answer?" Turolla A, Rossettini G, Viceconti A, Palese A, Geri T. Phys Ther. 2020;100:1260-1264." Phys Ther **100**(10): 1883.
Campbell, A. J., M. C. Robertson, M. M. Gardner, R. N. Norton and D. M. Buchner (1999). "Falls prevention over 2 years: a randomized controlled trial in women 80 years and older." Age Ageing **28**(6): 513-518.
Carey, J. R., W. K. Durfee, E. Bhatt, A. Nagpal, S. A. Weinstein, K. M. Anderson and S. M. Lewis (2007). "Comparison of Finger Tracking Versus Simple Movement Training via Telerehabilitation

to Alter Hand Function and Cortical Reorganization After Stroke." Neurorehabilitation and Neural Repair **21**(3): 216-232.
Celano, C. M., F. Gomez-Bernal, C. A. Mastromauro, E. E. Beale, C. M. DuBois, R. P. Auerbach and J. C. Huffman (2020). "A positive psychology intervention for patients with bipolar depression: a randomized pilot trial." Journal of Mental Health **29**(1): 60-68.
Chen, S. C., C. H. Lin, S. W. Su, Y. T. Chang and C. H. Lai (2021). "Feasibility and effect of interactive telerehabilitation on balance in individuals with chronic stroke: a pilot study." J Neuroeng Rehabil **18**(1): 71.
Chen, Y. P., C. Y. Lin, M. J. Tsai, T. Y. Chuang and O. K. S. Lee (2020). "Wearable motion sensor device to facilitate rehabilitation in patients with shoulder adhesive capsulitis: Pilot study to assess feasibility." Journal of Medical Internet Research **22**(7): e17032.
Chien, C. L., C. M. Lee, Y. W. Wu and Y. T. Wu (2011). "Home-based exercise improves the quality of life and physical function but not the psychological status of people with chronic heart failure: a randomised trial." J Physiother **57**(3): 157-163.
Choi, N. G., C. N. Marti, M. L. Bruce and M. T. Hegel (2013). "Depression in homebound older adults: problem-solving therapy and personal and social resourcefulness." Behav Ther **44**(3): 489-500.
Choi, N. G., C. N. Marti, M. L. Bruce, M. T. Hegel, N. L. Wilson and M. E. Kunik (2014). "Six-month postintervention depression and disability outcomes of in-home telehealth problem-solving therapy for depressed, low-income homebound older adults." Depress Anxiety **31**(8): 653-661.
Choi, N. G., C. N. Marti and Y. Conwell (2016). "Effect of Problem-Solving Therapy on Depressed Low-Income Homebound Older Adults' Death/Suicidal Ideation and Hopelessness." Suicide Life Threat Behav **46**(3): 323-336.
Chumbler Neale, R., P. Quigley, X. Li, M. Morey, D. Rose, J. Sanford, P. Griffiths and H. Hoenig (2012). "Effects of Telerehabilitation on Physical Function and Disability for Stroke Patients." Stroke **43**(8): 2168-2174.
Chumbler, N. R., X. Li, P. Quigley, M. C. Morey, D. Rose, P. Griffiths, J. Sanford and H. Hoenig (2015). "A randomized controlled trial on Stroke telerehabilitation: The effects on falls self-efficacy and satisfaction with care." Journal of Telemedicine and Telecare **21**(3): 139-143.
Chun, H. Y., A. J. Carson, A. Tsanas, M. S. Dennis, G. E. Mead, C. Calabria and W. N. Whiteley (2020). "Telemedicine Cognitive Behavioral Therapy for Anxiety After Stroke: Proof-of-Concept Randomized Controlled Trial." Stroke **51**(8): 2297-2306.
Church, D. and M. Clond (2019). "Is Online Treatment as Effective as In-Person Treatment?: Psychological Change in Two Relationship Skills Groups." J Nerv Ment Dis **207**(5): 315-319.
Cil, E. T., T. Serif, U. Sayli and F. Subasi (2023). "The effectiveness of "Dijital Steps" web based telerehabilitation system for patient with hindfoot pain: A randomised controlled trial." Foot **56**: 102040.
Claes, J., V. Cornelissen, C. McDermott, N. Moyna, N. Pattyn, N. Cornelis, A. Gallagher, C. McCormack, H. Newton, A. Gillain, W. Budts, K. Goetschalckx, C. Woods, K. Moran and R. Buys (2020). "Feasibility, Acceptability, and Clinical Effectiveness of a Technology-Enabled Cardiac Rehabilitation Platform (Physical Activity Toward Health-I): Randomized Controlled Trial." Journal of medical Internet research **22**(2): e14221.
Collins, A., C. L. Burns, E. C. Ward, T. Comans, C. Blake, L. Kenny, P. Greenup and D. Best (2017). "Home-based telehealth service for swallowing and nutrition management following head and neck cancer treatment." J Telemed Telecare **23**(10): 866-872.
Compen, F., E. Adang, E. Bisseling, M. van der Lee and A. Speckens (2020). "Cost-utility of individual internet-based and face-to-face Mindfulness-Based Cognitive Therapy compared with treatment as usual in reducing psychological distress in cancer patients." Psycho-oncology **29**(2): 294-303.
Compen, F., E. Bisseling, M. Schellekens, R. Donders, L. Carlson, M. van der Lee and A. Speckens (2018). "Face-to-face and internet-based mindfulness-based cognitive therapy

compared with treatment as usual in reducing psychological distress in patients with cancer: A multicenter randomized controlled trial." Journal of Clinical Oncology **36**(23): 2413-2421.
Corcoran, H., E. Hui and J. Woo (2003). "The acceptability of telemedicine for podiatric intervention in a residential home for the elderly." J Telemed Telecare **9**(3): 146-149.
Correia, F. D., A. Nogueira, I. Magalhães, J. Guimarães, M. Moreira, I. Barradas, M. Molinos, L. Teixeira, J. Tulha, R. Seabra, J. Lains and V. Bento (2019). "Medium-Term Outcomes of Digital Versus Conventional Home-Based Rehabilitation After Total Knee Arthroplasty: Prospective, Parallel-Group Feasibility Study." JMIR Rehabil Assist Technol **6**(1): e13111.
Cosio, D., L. Jin, J. Siddique and D. C. Mohr (2011). "The effect of telephone-administered cognitive-behavioral therapy on quality of life among patients with multiple sclerosis." Ann Behav Med **41**(2): 227-234.
Crow, S. J., J. E. Mitchell, R. D. Crosby, S. A. Swanson, S. Wonderlich and K. Lancanster (2009). "The cost effectiveness of cognitive behavioral therapy for bulimia nervosa delivered via telemedicine versus face-to-face." Behav Res Ther **47**(6): 451-453.
Cui, D., D. Janela, F. Costa, M. Molinos, A. C. Areias, R. G. Moulder, J. K. Scheer, V. Bento, S. P. Cohen, V. Yanamadala and F. D. Correia (2023). "Randomized-controlled trial assessing a digital care program versus conventional physiotherapy for chronic low back pain." NPJ Digital Medicine **6**(1): 1-10.
Czaja, S. J., D. Loewenstein, R. Schulz, S. N. Nair and D. Perdomo (2013). "A videophone psychosocial intervention for dementia caregivers." Am J Geriatr Psychiatry **21**(11): 1071-1081.
Dahmen, A., L. Gao, F. M. Keller, D. Lehr, P. Becker and S. Lippke (2022). "Curriculum Hannover - Web-based vs. Analogue Psychotherapeutic aftercare after Psychosomatic Rehabilitation and vs. Care as Usual." Die Rehabilitation **61**(4): 287-296.
Dalal, H. M., P. H. Evans, J. L. Campbell, R. S. Taylor, A. Watt, K. L. Read, A. J. Mourant, J. Wingham, D. R. Thompson and D. J. Pereira Gray (2007). "Home-based versus hospital-based rehabilitation after myocardial infarction: A randomized trial with preference arms--Cornwall Heart Attack Rehabilitation Management Study (CHARMS)." Int J Cardiol **119**(2): 202-211.
Dallolio, L., M. Menarini, S. China, M. Ventura, A. Stainthorpe, A. Soopramanien, P. Rucci and M. P. Fantini (2008). "Functional and Clinical Outcomes of Telemedicine in Patients With Spinal Cord Injury." Archives of Physical Medicine and Rehabilitation **89**(12): 2332-2341.
Davis, L. L., L. D. Burgio, K. C. Buckwalter and M. Weaver (2004). "A comparison of in-home and telephone-based skill training interventions with caregivers of persons with Dementia." Journal of Mental Health and Aging **10**(1): 31-44.
De Las Heras, J. C., F. Balbino, O. Hilberg, A. Lokke and E. Bendstrup (2020). "Is Virtual Autonomous Physiotherapist Tele-rehabilitation Program feasible in Idiopathic Pulmonary Fibrosis?" European Respiratory Journal **56**(Supplement 64).
De Luca, R., B. Aragona, S. Leonardi, M. Torrisi, B. Galletti, F. Galletti, M. Accorinti, P. Bramanti, M. C. De Cola and R. S. Calabrò (2018). "Computerized Training in Poststroke Aphasia: What About the Long-Term Effects? A Randomized Clinical Trial." J Stroke Cerebrovasc Dis **27**(8): 2271-2276.
De Luca, R., M. Russo, S. Gasparini, S. Leonardi, M. Foti Cuzzola, F. Sciarrone, C. Zichittella, E. Sessa, M. G. Maggio, M. C. De Cola and R. S. Calabrò (2021). "Do people with multiple sclerosis benefit from PC-based neurorehabilitation? A pilot study." Appl Neuropsychol Adult **28**(4): 427-435.
de Toledo, P., S. Jiménez, F. del Pozo, J. Roca, A. Alonso and C. Hernandez (2006). "Telemedicine experience for chronic care in COPD." IEEE Trans Inf Technol Biomed **10**(3): 567-573.
Dear, B. F., J. B. Zou, S. Ali, C. N. Lorian, L. Johnston, J. Sheehan, L. G. Staples, M. Gandy, V. J. Fogliati, B. Klein and N. Titov (2015). "Clinical and cost-effectiveness of therapist-guided internet-delivered cognitive behavior therapy for older adults with symptoms of anxiety: a randomized controlled trial." Behav Ther **46**(2): 206-217.

Diokno, A. C., D. K. Newman, L. K. Low, T. L. Griebling, M. E. Maddens, P. S. Goode, T. E. Raghunathan, L. L. Subak, C. M. Sampselle, J. A. Boura, A. E. Robinson, D. McIntyre and K. L. Burgio (2018). "Effect of Group-Administered Behavioral Treatment on Urinary Incontinence in Older Women: A Randomized Clinical Trial." JAMA Intern Med **178**(10): 1333-1341.
Dixon, P., S. Hollinghurst, L. Edwards, C. Thomas, D. Gaunt, A. Foster, S. Large, A. A. Montgomery and C. Salisbury (2016). "Cost-effectiveness of telehealth for patients with raised cardiovascular disease risk: evidence from the Healthlines randomised controlled trial." BMJ Open **6**(8): e012352.
Donkers, S. J., D. Nickel, L. Paul, S. R. Wiegers and K. B. Knox (2020). "Adherence to Physiotherapy-Guided Web-Based Exercise for Persons with Moderate-to-Severe Multiple Sclerosis: A Randomized Controlled Pilot Study." Int J MS Care **22**(5): 208-214.
Dorsey, E. R., L. M. Deuel, T. S. Voss, K. Finnigan, B. P. George, S. Eason, D. Miller, J. I. Reminick, A. Appler, J. Polanowicz, L. Viti, S. Smith, A. Joseph and K. M. Biglan (2010). "Increasing access to specialty care: a pilot, randomized controlled trial of telemedicine for Parkinson's disease." Mov Disord **25**(11): 1652-1659.
Dorstyn, D., J. Mathias, L. Denson and M. Robertson (2012). "Effectiveness of Telephone Counseling in Managing Psychological Outcomes After Spinal Cord Injury: A Preliminary Study." Archives of Physical Medicine and Rehabilitation **93**(11): 2100-2108.
Doze, S., J. Simpson, D. Hailey and P. Jacobs (1999). "Evaluation of a telepsychiatry pilot project." Journal of Telemedicine and Telecare **5**(1): 38-46.
Drozd, F., L. G. Skeie, P. Kraft and D. Kvale (2014). "A web-based intervention trial for depressive symptoms and subjective well-being in patients with chronic HIV infection." AIDS Care **26**(9): 1080-1089.
Durst, J., I. Roesel, G. Sudeck, K. Sassenberg and I. Krauss (2020). "Effectiveness of Human Versus Computer-Based Instructions for Exercise on Physical Activity-Related Health Competence in Patients with Hip Osteoarthritis: Randomized Noninferiority Crossover Trial." J Med Internet Res **22**(9): e18233.
Dwight-Johnson, M., E. Aisenberg, D. Golinelli, S. Hong, M. O'Brien and E. Ludman (2011). "Telephone-based cognitive-behavioral therapy for Latino patients living in rural areas: a randomized pilot study." Psychiatr Serv **62**(8): 936-942.
Egede, L. E., R. Acierno, R. G. Knapp, R. J. Walker, E. H. Payne and B. C. Frueh (2016). "Psychotherapy for Depression in Older Veterans Via Telemedicine: Effect on Quality of Life, Satisfaction, Treatment Credibility, and Service Delivery Perception." J Clin Psychiatry **77**(12): 1704-1711.
Egede, L. E., M. Gebregziabher, R. J. Walker, E. H. Payne, R. Acierno and B. C. Frueh (2017). "Trajectory of cost overtime after psychotherapy for depression in older Veterans via telemedicine." J Affect Disord **207**: 157-162.
Egede, L. E., R. J. Walker, E. H. Payne, R. G. Knapp, R. Acierno and B. C. Frueh (2018). "Effect of psychotherapy for depression via home telehealth on glycemic control in adults with type 2 diabetes: Subgroup analysis of a randomized clinical trial." J Telemed Telecare **24**(9): 596-602.
Egner, A., V. L. Phillips, R. Vora and E. Wiggers (2003). "Depression, fatigue, and health-related quality of life among people with advanced multiple sclerosis: results from an exploratory telerehabilitation study." NeuroRehabilitation **18**(2): 125-133.
Emmerson, K. B., K. E. Harding and N. F. Taylor (2017). "Home exercise programmes supported by video and automated reminders compared with standard paper-based home exercise programmes in patients with stroke: a randomized controlled trial." Clin Rehabil **31**(8): 1068-1077.
Eriksson, L., B. Lindström, G. Gard and J. Lysholm (2009). "Physiotherapy at a distance: a controlled study of rehabilitation at home after a shoulder joint operation." J Telemed Telecare **15**(5): 215-220.

Everitt, H. A., S. Landau, G. O'Reilly, A. Sibelli, S. Hughes, S. Windgassen, R. Holland, P. Little, P. McCrone, F. L. Bishop, K. Goldsmith, N. Coleman, R. Logan, T. Chalder and R. Moss-Morris (2019). "Cognitive behavioural therapy for irritable bowel syndrome: 24-month follow-up of participants in the ACTIB randomised trial." The Lancet Gastroenterology & Hepatology **4**(11): 863-872.

Fanuscu, A., M. Öz and O. Ulger (2023). "AB1402 THE EFFECTS OF SPINAL STABILIZATION EXERCISES VIA TELEREHABILITATION ON INDIVIDUALS WITH CHRONIC LOW BACK PAIN: A RANDOMIZED CONTROLLED STUDY." Annals of the Rheumatic Diseases **82**(Suppl 1): 1930.

Fernandez, E., J. A. Bergado Rosado, D. Rodriguez Perez, S. Salazar Santana, M. Torres Aguilar and M. L. Bringas (2017). "Effectiveness of a Computer-Based Training Program of Attention and Memory in Patients with Acquired Brain Damage." Behav Sci (Basel) **8**(1).

Finlayson, M., K. Preissner, C. Cho and M. Plow (2011). "Randomized trial of a teleconference-delivered fatigue management program for people with multiple sclerosis." Mult Scler **17**(9): 1130-1140.

Flynn, A., E. Preston, S. Dennis, C. Canning and N. Allen (2020). "Remotely-monitored home-based physiotherapy is feasible for both people with Parkinson's disease and physiotherapists." Movement Disorders **35**(SUPPL 1): S543.

Forducey, P. G., R. L. Glueckauf, T. F. Bergquist, M. M. Maheu and M. Yutsis (2012). "Telehealth for persons with severe functional disabilities and their caregivers: facilitating self-care management in the home setting." Psychol Serv **9**(2): 144-162.

Fortney, J. C., J. M. Pyne, M. J. Edlund, D. K. Williams, D. E. Robinson, D. Mittal and K. L. Henderson (2007). "A randomized trial of telemedicine-based collaborative care for depression." J Gen Intern Med **22**(8): 1086-1093.

Franklin, C. L., L. A. Cuccurullo, J. L. Walton, J. R. Arseneau and N. J. Petersen (2017). "Face to face but not in the same place: A pilot study of prolonged exposure therapy." Journal of Trauma and Dissociation **18**(1): 116-130.

Frederix, I., F. Solmi, M. F. Piepoli and P. Dendale (2017). "Cardiac telerehabilitation: A novel cost-efficient care delivery strategy that can induce long-term health benefits." Eur J Prev Cardiol **24**(16): 1708-1717.

Frederix, I., N. Van Driessche, D. Hansen, J. Berger, K. Bonne, T. Alders and P. Dendale (2015). "Increasing the medium-term clinical benefits of hospital-based cardiac rehabilitation by physical activity telemonitoring in coronary artery disease patients." Eur J Prev Cardiol **22**(2): 150-158.

Frueh, B. C., J. Monnier, E. Yim, A. L. Grubaugh, M. B. Hamner and R. G. Knapp (2007). "A randomized trial of telepsychiatry for post-traumatic stress disorder." J Telemed Telecare **13**(3): 142-147.

Garcia-Palacios, A., R. Herrero, Y. Vizcaíno, M. A. Belmonte, D. Castilla, G. Molinari, R. M. Baños and C. Botella (2015). "Integrating virtual reality with activity management for the treatment of fibromyalgia: acceptability and preliminary efficacy." Clin J Pain **31**(6): 564-572.

Giallauria, F., R. Lucci, F. Pilerci, A. De Lorenzo, A. Manakos, M. Psaroudaki, M. Dagostino, A. Vitelli, L. Maresca, D. Del Forno and C. Vigorito (2006). "Efficacy of telecardiology in improving the results of cardiac rehabilitation after acute myocardial infarction." Monaldi Arch Chest Dis **66**(1): 8-12.

Gibson, K. L., H. Coulson, R. Miles, C. Kakekakekung, E. Daniels and S. O'Donnell (2011). "Conversations on telemental health: listening to remote and rural First Nations communities." Rural Remote Health **11**(2): 1656.

Ginis, P., A. Nieuwboer, M. Dorfman, A. Ferrari, E. Gazit, C. G. Canning, L. Rocchi, L. Chiari, J. M. Hausdorff and A. Mirelman (2016). "Feasibility and effects of home-based smartphone-delivered automated feedback training for gait in people with Parkinson's disease: A pilot randomized controlled trial." Parkinsonism Relat Disord **22**: 28-34.

Glueckauf, R. L., W. S. Davis, F. B. Willis, D. Sharma, D. J. Gustafson, J. Hayes, M. M. Stutzman, J. B. Proctor, M. M. Kazmer, L. Murray, J. Shipman, V. McIntyre, L. Wesley, G. Schettini, J. Xu, F. C. Parfitt, N. R. Graff-Radford, C. Baxter, K. Burnett, L. T. Noël, K. K. Haire and J. Springer (2012). "Telephone-based, cognitive-behavioral therapy for African American dementia caregivers with depression: initial findings." Rehabilitation psychology **57 2**: 124-139.
Gohir, S. A., F. Eek, A. Kelly, A. Abhishek and A. M. Valdes (2021). "Effectiveness of Internet-Based Exercises Aimed at Treating Knee Osteoarthritis: The iBEAT-OA Randomized Clinical Trial." JAMA Netw Open **4**(2): e210012.
Gordon, N. F., C. D. English, A. S. Contractor, R. D. Salmon, R. F. Leighton, B. A. Franklin and W. L. Haskell (2002). "Effectiveness of three models for comprehensive cardiovascular disease risk reduction." The American Journal of Cardiology **89**(11): 1263-1268.
Greene, C. J., L. A. Morland, A. Macdonald, B. C. Frueh, K. M. Grubbs and C. S. Rosen (2010). "How does tele-mental health affect group therapy process? Secondary analysis of a noninferiority trial." J Consult Clin Psychol **78**(5): 746-750.
Gros, D. F., C. L. Lancaster, C. M. López and R. Acierno (2018). "Treatment satisfaction of home-based telehealth versus in-person delivery of prolonged exposure for combat-related PTSD in veterans." J Telemed Telecare **24**(1): 51-55.
Gros, D. F., M. Yoder, P. W. Tuerk, B. E. Lozano and R. Acierno (2011). "Exposure therapy for PTSD delivered to veterans via telehealth: predictors of treatment completion and outcome and comparison to treatment delivered in person." Behav Ther **42**(2): 276-283.
Grubbs, K. M., J. C. Fortney, T. Dean, J. S. Williams and L. Godleski (2015). "A Comparison of Mental Health Diagnoses Treated via Interactive Video and Face to Face in the Veterans Healthcare Administration." Telemed J E Health **21**(7): 564-566.
Grzincich, G., R. Gagliardini, A. Bossi, S. Bella, G. Cimino, N. Cirilli, L. Viviani, E. Iacinti and S. Quattrucci (2010). "Evaluation of a home telemonitoring service for adult patients with cystic fibrosis: a pilot study." J Telemed Telecare **16**(7): 359-362.
Gustafsson, L. (2020). "Occupational therapy has gone online: What will remain beyond COVID-19?" Australian Occupational Therapy Journal **67**(3): 197-198.
Gutiérrez, R. O., F. Galán Del Río, R. Cano de la Cuerda, I. M. Alguacil Diego, R. A. González and J. C. M. Page (2013). "A telerehabilitation program by virtual reality-video games improves balance and postural control in multiple sclerosis patients." NeuroRehabilitation **33**(4): 545-554.
Hagen, K. B. and M. Grotle (2017). "Internet-delivered physiotherapist-prescribed exercise and pain-coping skills training is beneficial for people with chronic knee pain [synopsis]." J Physiother **63**(4): 260.
Hagovská, M., O. Dzvoník and Z. Olekszyová (2017). "Comparison of Two Cognitive Training Programs With Effects on Functional Activities and Quality of Life." Res Gerontol Nurs **10**(4): 172-180.
Hall, D. L., E. G. Lattie, S. F. Milrad, S. Czaja, M. A. Fletcher, N. Klimas, D. Perdomo and M. H. Antoni (2017). "Telephone-administered versus live group cognitive behavioral stress management for adults with CFS." J Psychosom Res **93**: 41-47.
Hanssen, T. A., J. E. Nordrehaug, G. E. Eide and B. R. Hanestad (2007). "Improving outcomes after myocardial infarction: a randomized controlled trial evaluating effects of a telephone follow-up intervention." European Journal of Cardiovascular Prevention & Rehabilitation **14**(3): 429-437.
Hanssen, T. A., J. E. Nordrehaug, G. E. Eide and B. R. Hanestad (2009). "Does a telephone follow-up intervention for patients discharged with acute myocardial infarction have long-term effects on health-related quality of life? A randomised controlled trial." J Clin Nurs **18**(9): 1334-1345.

Hassall, S. L., R. Wootton and C. Guilfoyle (2003). "The cost of allied health assessments delivered by videoconference to a residential facility for elderly people." Journal of Telemedicine and Telecare **9**: 234 - 237.
Heckman, T. G., B. D. Heckman, T. Anderson, T. I. Lovejoy, J. C. Markowitz, Y. Shen and M. Sutton (2017). "Tele-Interpersonal Psychotherapy Acutely Reduces Depressive Symptoms in Depressed HIV-Infected Rural Persons: A Randomized Clinical Trial." Behav Med **43**(4): 285-295.
Heckman, T. G., B. D. Heckman, T. Anderson, T. I. Lovejoy, D. Mohr, M. Sutton, J. A. Bianco and J. T. Gau (2013). "Supportive-expressive and coping group teletherapies for HIV-infected older adults: a randomized clinical trial." AIDS Behav **17**(9): 3034-3044.
Heckman, T. G., J. C. Markowitz, B. D. Heckman, H. Woldu, T. Anderson, T. I. Lovejoy, Y. Shen, M. Sutton and W. Yarber (2018). "A Randomized Clinical Trial Showing Persisting Reductions in Depressive Symptoms in HIV-Infected Rural Adults Following Brief Telephone-Administered Interpersonal Psychotherapy." Ann Behav Med **52**(4): 299-308.
Hedman, E., G. Andersson, B. Ljótsson, E. Andersson, C. Rück, E. Mörtberg and N. Lindefors (2011). "Internet-based cognitive behavior therapy vs. cognitive behavioral group therapy for social anxiety disorder: a randomized controlled non-inferiority trial." PLoS One **6**(3): e18001.
Hegel, M. T., K. D. Lyons, J. G. Hull, P. Kaufman, L. Urquhart, Z. Li and T. A. Ahles (2011). "Feasibility study of a randomized controlled trial of a telephone-delivered problem-solving-occupational therapy intervention to reduce participation restrictions in rural breast cancer survivors undergoing chemotherapy." Psychooncology **20**(10): 1092-1101.
Hernandez-Tejada, M. A., J. S. Zoller, K. J. Ruggiero, A. S. Kazley and R. Acierno (2014). "Early treatment withdrawal from evidence-based psychotherapy for PTSD: telemedicine and in-person parameters." International Journal of Psychiatry in Medicine **48**(1): 33-55.
Hernando-Requejo, V., N. Huertas-González, J. Lapeña-Motilva and G. Ogando-Durán "The epilepsy unit during the COVID-19 epidemic: the role of telemedicine and the effects of confinement on patients with epilepsy." (2173-5808 (Print)).
Horton, B. S., J. D. Marland, H. S. West and J. D. Wylie (2021). "Transition to Telehealth Physical Therapy After Hip Arthroscopy for Femoroacetabular Impingement." Orthopaedic Journal of Sports Medicine **9**(4): 1-6.
Hou, J., R. Yang, Y. Yang, Y. Tang, H. Deng, Z. Chen, Y. Wu and H. Shen (2019). "The Effectiveness and Safety of Utilizing Mobile Phone-Based Programs for Rehabilitation After Lumbar Spinal Surgery: Multicenter, Prospective Randomized Controlled Trial." JMIR MHealth and UHealth **7**(2): e10201.
Hurley, E., K. Vasavada, C. Lin, G. Gonzalez-Lomas, M. Alaia, E. Strauss, L. Jazrawi, K. Campbell and E. Mojica (2022). "Poster 236: There is No Difference in Early Functional Outcomes for Patients Undergoing Tele-Rehabilitation versus Standard in Office Rehab after Arthroscopic Meniscectomy: A Randomized Controlled Trial." Orthopaedic Journal of Sports Medicine **10**(7_suppl5): 2325967121S2325900797.
Iles, R., N. F. Taylor, M. Davidson and P. O'Halloran (2011). "Telephone coaching can increase activity levels for people with non-chronic low back pain: a randomised trial." J Physiother **57**(4): 231-238.
Jaconis, M., E. J. Santa Ana, T. K. Killeen, C. L. Badour and S. E. Back (2017). "Concurrent treatment of PTSD and alcohol use disorder via telehealth in a female Iraq veteran." Am J Addict **26**(2): 112-114.
Jansen-Kosterink, S., R. Huis in 't Veld, D. Wever, H. Hermens and M. Vollenbroek-Hutten (2015). "Introducing remote physical rehabilitation for patients with chronic disorders by means of telemedicine." Health and Technology **5**(2): 83-90.
Jansons, P., L. Robins, L. O'Brien and T. Haines (2017). "Gym-based exercise and home-based exercise with telephone support have similar outcomes when used as maintenance programs in adults with chronic health conditions: a randomised trial." Journal of physiotherapy **63**(3): 154-160.

Jarbandhan, A., J. Toelsie, D. Veeger, R. Bipat, L. Vanhees and R. Buys (2022). "Feasibility of a home-based physiotherapy intervention to promote post-stroke mobility: A randomized controlled pilot study." PLoS ONE **17**(3 March 2022): e0256455.
Jarvela-Reijonen, E., L. Karhunen, E. Sairanen, J. Muotka, S. Lindroos, J. Laitinen, S. Puttonen, K. Peuhkuri, M. Hallikainen, J. Pihlajamaki, R. Korpela, M. Ermes, R. Lappalainen and M. Kolehmainen (2018). "The effects of acceptance and commitment therapy on eating behavior and diet delivered through face-to-face contact and a mobile app: A randomized controlled trial." International Journal of Behavioral Nutrition and Physical Activity **15**(1): 22.
Jelinek, M., M. J. Vale, D. Liew, L. Grigg, A. Dart, D. L. Hare and J. D. Best (2009). "The COACH program produces sustained improvements in cardiovascular risk factors and adherence to recommended medications-two years follow-up." Heart Lung Circ **18**(6): 388-392.
Jiang, Y., K. W. L. Koh, H. J. Ramachandran, H. D. Nguyen, D. S. Lim, Y. K. Tay, S. Shorey and W. Wang (2021). "The effectiveness of a nurse-led home-based heart failure self-management programme (the HOM-HEMP) for patients with chronic heart failure: A three-arm stratified randomized controlled trial." International Journal of Nursing Studies **122**: N.PAG-N.PAG.
Jódar-Sánchez, F., F. Ortega, C. Parra, C. Gómez-Suárez, P. Bonachela, S. Leal, P. Pérez, A. Jordán and E. Barrot (2014). "Cost-utility analysis of a telehealth programme for patients with severe chronic obstructive pulmonary disease treated with long-term oxygen therapy." Journal of Telemedicine and Telecare **20**(6): 307-316.
Jolly, K., G. Y. Lip, J. Sandercock, S. M. Greenfield, J. P. Raftery, J. Mant, R. Taylor, D. Lane, K. W. Lee and A. J. Stevens (2003). "Home-based versus hospital-based cardiac rehabilitation after myocardial infarction or revascularisation: design and rationale of the Birmingham Rehabilitation Uptake Maximisation Study (BRUM): a randomised controlled trial [ISRCTN72884263]." BMC Cardiovasc Disord **3**: 10.
Jolly, K., G. Y. Lip, R. S. Taylor, J. Raftery, J. Mant, D. Lane, S. Greenfield and A. Stevens (2009). "The Birmingham Rehabilitation Uptake Maximisation study (BRUM): a randomised controlled trial comparing home-based with centre-based cardiac rehabilitation." Heart **95**(1): 36-42.
Jolly, K., R. Taylor, G. Y. Lip, S. Greenfield, J. Raftery, J. Mant, D. Lane, M. Jones, K. W. Lee and A. Stevens (2007). "The Birmingham Rehabilitation Uptake Maximisation Study (BRUM). Home-based compared with hospital-based cardiac rehabilitation in a multi-ethnic population: cost-effectiveness and patient adherence." Health Technol Assess **11**(35): 1-118.
Jung, Y. H., S. C. Park, J. H. Lee, M. J. Kim, S. Lee, S. J. Chung, J. Y. Moon, Y. H. Choi, J. Ju, H. J. Han and S. Y. Lee (2023). "Effect of internet-based vs. in-person multimodal interventions on patients with mild to moderate Alzheimer's disease: a randomized, cross-over, open-label trial." Frontiers in public health **11**: 1203201.
Kalapatapu, R. K., J. Ho, X. Cai, S. Vinogradov, S. L. Batki and D. C. Mohr (2014). "Cognitive-behavioral therapy in depressed primary care patients with co-occurring problematic alcohol use: effect of telephone-administered vs. face-to-face treatment-a secondary analysis." J Psychoactive Drugs **46**(2): 85-92.
Kaldo, V., S. Levin, J. Widarsson, M. Buhrman, H. C. Larsen and G. Andersson (2008). "Internet versus group cognitive-behavioral treatment of distress associated with tinnitus: a randomized controlled trial." Behav Ther **39**(4): 348-359.
Kallestad, H., J. Scott, Ø. Vedaa, S. Lydersen, D. Vethe, G. Morken, T. C. Stiles, B. Sivertsen and K. Langsrud (2021). "Mode of delivery of Cognitive Behavioral Therapy for Insomnia: a randomized controlled non-inferiority trial of digital and face-to-face therapy." Sleep **44**(12).
Kalron, A., H. Tawil, S. Peleg-Shani and J.-J. Vatine (2018). "Effect of telerehabilitation on mobility in people after hip surgery: a pilot feasibility study." International Journal of Rehabilitation Research **41**: 244–250.
Kasnakova, P., A. Mihaylova, B. Djurdjev and B. Tornyova (2022). "Randomized controlled trial of multidisciplinary rehabilitation therapy using mobile applications in cases of ankle fractures." Eur J Transl Myol **32**(2).

Kenny, M., J. Gilmartin and C. Thompson (2022). Video-guided exercise after stroke: a feasibility randomised controlled trial. Philadelphia, Pennsylvania, Taylor & Francis Ltd. **38:** 609-620.
Keskin, Y., A. Gurcan Atci, B. Urkmez, Y. S. Akgul, N. Ozaras and T. Aydin (2020). "Efficacy of a video-based physical therapy and rehabilitation system in patients with post-stroke hemiplegia: A randomized, controlled, pilot study." Turk Geriatri Dergisi **23**(1): 118-128.
Khalil, H., M. Busse, L. Quinn, M. Nazzal, W. Batyha, S. Alkhazaleh and M. A. Alomari (2017). "A pilot study of a minimally supervised home exercise and walking program for people with Parkinson's disease in Jordan." Neurodegener Dis Manag **7**(1): 73-84.
Kidholm, K., M. K. Rasmussen, J. J. Andreasen, J. Hansen, G. Nielsen, H. Spindler and B. Dinesen (2016). "Cost-Utility Analysis of a Cardiac Telerehabilitation Program: The Teledialog Project." Telemed J E Health **22**(7): 553-563.
Kim, H., J. H. Jhoo and J. W. Jang (2017). "The effect of telemedicine on cognitive decline in patients with dementia." J Telemed Telecare **23**(1): 149-154.
Kirkness, C. J., K. C. Cain, K. J. Becker, D. L. Tirschwell, A. M. Buzaitis, P. L. Weisman, S. McKenzie, L. Teri, R. Kohen, R. C. Veith and P. H. Mitchell (2017). "Randomized trial of telephone versus in-person delivery of a brief psychosocial intervention in post-stroke depression." BMC Res Notes **10**(1): 500.
Kizony, R., P. L. Weiss, Y. Feldman, M. Shani, O. Elion, S. Harel and I. Baum-Cohen (2013). "Evaluation of a Tele-Health System for upper extremity stroke rehabilitation." 2013 International Conference on Virtual Rehabilitation (ICVR): 80-86.
Klee, A., M. Stacy, R. Rosenheck, L. Harkness and J. Tsai (2016). "Interest in technology-based therapies hampered by access: A survey of veterans with serious mental illnesses." Psychiatr Rehabil J **39**(2): 173-179.
Kloek, C. J. J., D. Bossen, P. M. Spreeuwenberg, J. Dekker, D. H. de Bakker and C. Veenhof (2018). "Effectiveness of a Blended Physical Therapist Intervention in People With Hip Osteoarthritis, Knee Osteoarthritis, or Both: A Cluster-Randomized Controlled Trial." Physical therapy **98**(7): 560-570.
Kloek, C. J. J., J. M. van Dongen, D. H. de Bakker, D. Bossen, J. Dekker and C. Veenhof (2018). "Cost-effectiveness of a blended physiotherapy intervention compared to usual physiotherapy in patients with hip and/or knee osteoarthritis: a cluster randomized controlled trial." BMC Public Health **18**(1): 1082.
Kroenke, K., E. E. Krebs, J. Wu, Z. Yu, N. R. Chumbler and M. J. Bair (2014). "Telecare collaborative management of chronic pain in primary care: a randomized clinical trial." Jama **312**(3): 240-248.
Krpič, A., A. Savanović and I. Cikajlo (2013). "Telerehabilitation: remote multimedia-supported assistance and mobile monitoring of balance training outcomes can facilitate the clinical staff's effort." Int J Rehabil Res **36**(2): 162-171.
Kryger, M. A., T. M. Crytzer, A. Fairman, E. J. Quinby, M. Karavolis, G. Pramana, I. M. A. Setiawan, G. P. McKernan, B. Parmanto and B. E. Dicianno (2019). "The Effect of the Interactive Mobile Health and Rehabilitation System on Health and Psychosocial Outcomes in Spinal Cord Injury: Randomized Controlled Trial." J Med Internet Res **21**(8): e14305.
Kuster, A. T., T. K. Dalsbø, B. Y. Luong Thanh, A. Agarwal, Q. V. Durand-Moreau and I. Kirkehei (2017). "Computer-based versus in-person interventions for preventing and reducing stress in workers." Cochrane Database Syst Rev **8**(8): Cd011899.
Lee, Y. H., S. H. Hur, J. Sohn, H. M. Lee, N. H. Park, Y. K. Cho, H. S. Park, H. J. Yoon, H. Kim, C. W. Nam, Y. N. Kim and K. B. Kim (2013). "Impact of home-based exercise training with wireless monitoring on patients with acute coronary syndrome undergoing percutaneous coronary intervention." J Korean Med Sci **28**(4): 564-568.
Li, C. T., G. K. Hung, K. N. Fong, P. C. Gonzalez, S.-H. Wah and H. W. Tsang (2022). "Effects of home-based occupational therapy telerehabilitation via smartphone for outpatients after hip

fracture surgery: A feasibility randomised controlled study." Journal of telemedicine and telecare **28**(4): 239-247.
Li, X., L. Zhao, T. Xu, G. Shi, J. Li, W. Shuai, Y. Yang, W. Tian and Y. Zhou (2023). "Cardiac telerehabilitation under 5G internet of things monitoring: a randomized pilot study." Scientific reports **13**(1): 18886.
Lightstone, A., K. Bailey and P. Voros (2015). "Collaborative Music Therapy via Remote Video Technology to Reduce a Veteran's Symptoms of Severe, Chronic PTSD." Arts & Health **7**: 123-136.
Linder, S. M., A. B. Rosenfeldt, R. C. Bay, K. Sahu, S. L. Wolf and J. L. Alberts (2015). "Improving Quality of Life and Depression After Stroke Through Telerehabilitation." Am J Occup Ther **69**(2): 6902290020p6902290021-6902290010.
Lindsay, J. A., M. R. Kauth, S. Hudson, L. A. Martin, D. J. Ramsey, L. Daily and J. Rader (2015). "Implementation of video telehealth to improve access to evidence-based psychotherapy for posttraumatic stress disorder." Telemed J E Health **21**(6): 467-472.
LoSavio, S. T., C. B. Worley, S. T. Aajmain, C. S. Rosen, S. Wiltsey Stirman and D. M. Sloan (2023). "Effectiveness of written exposure therapy for posttraumatic stress disorder in the Department of Veterans Affairs Healthcare System." Psychol Trauma **15**(5): 748-756.
Lovell, K., L. Fullalove, R. Garvey and C. Brooker (2000). "TELEPHONE TREATMENT OF OBSESSIVE-COMPULSIVE DISORDER." Behavioural and Cognitive Psychotherapy **28**(1): 87-91.
Luangapichart, P., N. Saisavoey and N. Viravan (2022). "Efficacy and Feasibility of the Minimal Therapist-Guided Four-Week Online Audio-Based Mindfulness Program 'Mindful Senses' for Burnout and Stress Reduction in Medical Personnel: A Randomized Controlled Trial." Healthcare (Basel) **10**(12).
Luxton, D. D., L. D. Pruitt, K. O'Brien and G. M. Kramer (2015). "An Evaluation of the Feasibility and Safety of a Home-Based Telemental Health Treatment for Posttraumatic Stress in the U.S. Military." Telemedicine journal and e-health : the official journal of the American Telemedicine Association **21 11**: 880-886.
Maddison, R., L. Pfaeffli, R. Whittaker, R. Stewart, A. Kerr, Y. Jiang, G. Kira, W. Leung, L. Dalleck, K. Carter and J. Rawstorn (2015). "A mobile phone intervention increases physical activity in people with cardiovascular disease: Results from the HEART randomized controlled trial." European Journal of Preventive Cardiology **22**(6): 701-709.
Maisiak, R., J. Austin and L. Heck (1996). "Health outcomes of two telephone interventions for patients with rheumatoid arthritis or osteoarthritis." Arthritis & Rheumatism **39**(8): 1391-1399.
Mallet, K. H., R. M. Shamloul, J. Lecompte-Collin, J. Winkel, B. Donnelly and D. Dowlatshahi (2023). "Telerehab at Home: Mobile Tablet Technology for Patients With Poststroke Communication Deficits-A Pilot Feasibility Randomized Control Trial." J Speech Lang Hear Res **66**(2): 648-655.
Maltais, F., J. Bourbeau, S. Shapiro, Y. Lacasse, H. Perrault, M. Baltzan, P. Hernandez, M. Rouleau, M. Julien, S. Parenteau, B. Paradis, R. D. Levy, P. Camp, R. Lecours, R. Audet, B. Hutton, J. R. Penrod, D. Picard and S. Bernard (2008). "Effects of home-based pulmonary rehabilitation in patients with chronic obstructive pulmonary disease: a randomized trial." Ann Intern Med **149**(12): 869-878.
Man, D. W., W. Y. Soong, S. F. Tam and C. W. Hui-Chan (2006). "A randomized clinical trial study on the effectiveness of a tele-analogy-based problem-solving programme for people with acquired brain injury (ABI)." NeuroRehabilitation **21**(3): 205-217.
Maresca, G., M. G. Maggio, D. Latella, A. Cannavo, M. C. De Cola, S. Portaro, M. C. Stagnitti, G. Silvestri, M. Torrisi, A. Bramanti, R. De Luca and R. S. Calabro (2019). "Toward Improving Poststroke Aphasia: A Pilot Study on the Growing Use of Telerehabilitation for the Continuity of Care." Journal of Stroke and Cerebrovascular Diseases **28**(10): 104303.

Marino, F., P. Chilà, C. Failla, I. Crimi, R. Minutoli, A. Puglisi, A. Arnao, G. Tartarisco, L. Ruta, D. Vagni and G. Pioggia (2020). "Tele-Assisted Behavioral Intervention for Families with Children with Autism Spectrum Disorders: A Randomized Control Trial." Brain Sciences **10**.
Marziali, E. (2009). "E-health program for patients with chronic disease." Telemed J E Health **15**(2): 176-181.
Marziali, E. and P. Donahue (2006). "Caring for others: Internet video-conferencing group intervention for family caregivers of older adults with neurodegenerative disease." Gerontologist **46**(3): 398-403.
Mashima, P. A., D. P. Birkmire-Peters, M. J. Syms, M. R. Holtel, L. P. Burgess and L. J. Peters (2003). "Telehealth: voice therapy using telecommunications technology." Am J Speech Lang Pathol **12**(4): 432-439.
Mayo, N. E., L. Nadeau, S. Ahmed, C. White, R. Grad, A. Huang, M. J. Yaffe and S. Wood-Dauphinee (2008). "Bridging the gap: the effectiveness of teaming a stroke coordinator with patient's personal physician on the outcome of stroke." Age Ageing **37**(1): 32-38.
Mayor-Silva, L. I., M. Romero-Saldaña, A. G. Moreno-Pimentel, Á. Álvarez-Melcón, R. Molina-Luque and A. Meneses-Monroy (2021). "The role of psychological variables in improving resilience: Comparison of an online intervention with a face-to-face intervention. A randomised controlled clinical trial in students of health sciences." Nurse Education Today **99**: N.PAG-N.PAG.
McBeth, J., G. Prescott, G. Scotland, K. Lovell, P. Keeley, P. Hannaford, P. McNamee, D. P. Symmons, S. Woby, C. Gkazinou, M. Beasley and G. J. Macfarlane (2012). "Cognitive behavior therapy, exercise, or both for treating chronic widespread pain." Arch Intern Med **172**(1): 48-57.
Meichsner, F., N. F. Töpfer, M. Reder, R. Soellner and G. Wilz (2019). "Telephone-Based Cognitive Behavioral Intervention Improves Dementia Caregivers' Quality of Life." Am J Alzheimers Dis Other Demen **34**(4): 236-246.
Meng, H., V. R. Marino, K. O. Conner, D. Sharma, W. S. Davis and R. L. Glueckauf (2021). "Effects of in-person and telephone-based cognitive behavioral therapies on health services use and expenditures among African-American dementia caregivers with depressive symptoms." Ethnicity & health **26**(6): 879-892.
Messinis, L., G. Nasios, M. H. Kosmidis, P. Zampakis, S. Malefaki, K. Ntoskou, A. Nousia, C. Bakirtzis, N. Grigoriadis, P. Gourzis and P. Papathanasopoulos (2017). "Efficacy of a Computer-Assisted Cognitive Rehabilitation Intervention in Relapsing-Remitting Multiple Sclerosis Patients: A Multicenter Randomized Controlled Trial." Behavioural Neurology **2017**: 5919841.
Miller, N. H., W. L. Haskell, K. Berra and R. F. DeBusk (1984). "Home versus group exercise training for increasing functional capacity after myocardial infarction." Circulation **70**(4): 645-649.
Mohr, D. C., T. Carmody, L. Erickson, L. Jin and J. Leader (2011). "Telephone-administered cognitive behavioral therapy for veterans served by community-based outpatient clinics." J Consult Clin Psychol **79**(2): 261-265.
Mohr, D. C., S. Hart and L. Vella (2007). "Reduction in disability in a randomized controlled trial of telephone-administered cognitive-behavioral therapy." Health Psychol **26**(5): 554-563.
Mohr, D. C., W. Likosky, A. Bertagnolli, D. E. Goodkin, J. Van Der Wende, P. Dwyer and L. P. Dick (2000). "Telephone-administered cognitive-behavioral therapy for the treatment of depressive symptoms in multiple sclerosis." J Consult Clin Psychol **68**(2): 356-361.
Mojica, E. S., K. Vasavada, E. T. Hurley, C. C. Lin, S. Buzin, G. Gonzalez-Lomas, M. J. Alaia, E. J. Strauss, L. M. Jazrawi and K. A. Campbell (2023). "Despite Equivalent Clinical Outcomes, Patients Report Less Satisfaction With Telerehabilitation Versus Standard In-Office Rehabilitation After Arthroscopic Meniscectomy: A Randomized Controlled Trial." Arthroscopy, Sports Medicine, and Rehabilitation **5**(2): e395-e401.

Morland, L. A., M. Raab, M. A. Mackintosh, C. S. Rosen, C. E. Dismuke, C. J. Greene and B. C. Frueh (2013). "Telemedicine: a cost-reducing means of delivering psychotherapy to rural combat veterans with PTSD." Telemed J E Health **19**(10): 754-759.
Mottaghi, S., I. Rahimian Boogar, S. Moradi and N. Sotodehasl (2022). "Effectiveness of Face-to-Face and Online Methods of Cognitive-Behavioral Therapy Based on Stress Reduction on Diabetes Management Self-Efficacy and Adherence to Treatment among Patients with Diabetes during the Coronavirus Pandemic." zbmu-jdn **10**(2): 1844-1861.
Munro, A. J., T. Shaw, L. Clarke, L. Becker and S. Greenwood (1994). "A randomized study of telephone contact following completion of radiotherapy." Clinical oncology (Royal College of Radiologists (Great Britain)) **6 4**: 242-244.
Nambi, G., M. Alghadier, A. Vellaiyan, E. E. Ebrahim, O. R. Aldhafian, S. H. P. Mohamed, H. F. A. Albalawi, M. F. Chevidikunnan, F. Khan, P. Mani, A. K. Saleh and N. N. Alshahrani (2023). "Role of Tele-Physical Therapy Training on Glycemic Control, Pulmonary Function, Physical Fitness, and Health-Related Quality of Life in Patients with Type 2 Diabetes Mellitus (T2DM) Following COVID-19 Infection—A Randomized Controlled Trial." Healthcare (2227-9032) **11**(12): 1791.
Naylor, M. R., F. J. Keefe, B. Brigidi, S. Naud and J. E. Helzer (2008). "Therapeutic Interactive Voice Response for chronic pain reduction and relapse prevention." Pain **134**(3): 335-345.
Nelson, M., M. Bourke, K. Crossley and T. Russell (2020). "Telerehabilitation is non-inferior to usual care following total hip replacement — a randomized controlled non-inferiority trial." Physiotherapy **107**: 19-27.
Neubeck, L., S. B. Freedman, T. Briffa, A. Bauman and J. Redfern (2011). "Four-year follow-up of the Choice of Health Options In prevention of Cardiovascular Events randomized controlled trial." European Journal of Cardiovascular Prevention & Rehabilitation **18**(2): 278-286.
North, S. (2020). "Addressing Students' Mental Health Needs via Telehealth." N C Med J **81**(2): 112-113.
Novella, J. K., K.-M. Ng and J. Samuolis (2022). "A comparison of online and in-person counseling outcomes using solution-focused brief therapy for college students with anxiety." Journal of American College Health **70**(4): 1161-1168.
Oerkild, B., M. S. Frederiksen, J. F. Hansen, L. Simonsen, L. T. Skovgaard and E. I. B. Prescott (2011). "Home-based cardiac rehabilitation is as effective as centre-based cardiac rehabilitation among elderly with coronary heart disease: results from a randomised clinical trial." Age and ageing **40 1**: 78-85.
Ofoegbu, T. O., U. Asogwa, M. S. Otu, C. Ibenegbu, A. Muhammed and B. Eze (2020). "Efficacy of guided internet-assisted intervention on depression reduction among educational technology students of Nigerian universities." Medicine **99**(6): e18774-e18774.
Oka, R. K., T. De Marco, W. L. Haskell, E. Botvinick, M. W. Dae, K. Bolen and K. Chatterjee (2000). "Impact of a home-based walking and resistance training program on quality of life in patients with heart failure." The American Journal of Cardiology **85**(3): 365-369.
Onan, D. and O. Ulger (2022). "THE EFFECTS of TELEREHABILITATION with REMOTELY SPINAL STABILIZATION EXERCISES on CLINICAL VARIABLES, FUNCTIONAL ACTIVITIES, EXERCISE ADHERENCE and MUSCLE ARCHITECTURE of NECK MUSCLES in INDIVIDUALS with CHRONIC NECK PAIN." Annals of the Rheumatic Diseases **81**(Supplement 1): 229.
Ora, H. P., M. Kirmess, M. C. Brady, I. Partee, R. B. Hognestad, B. B. Johannessen, B. Thommessen and F. Becker (2020). "The effect of augmented speech-language therapy delivered by telerehabilitation on poststroke aphasia-a pilot randomized controlled trial." Clinical rehabilitation **34**(3): 369-381.
Ortiz-Gutiérrez, R., R. Cano-de-la-Cuerda, F. Galán-del-Río, I. M. Alguacil-Diego, D. Palacios-Ceña and J. C. Miangolarra-Page (2013). "A telerehabilitation program improves postural control in multiple sclerosis patients: a Spanish preliminary study." Int J Environ Res Public Health **10**(11): 5697-5710.

Ownsworth, T., S. Chambers, S. Jones, G. Parker, J. F. Aitken, M. Foote, L. G. Gordon, D. H. K. Shum, J. Robertson, E. Conlon and M. B. Pinkham (2023). "Evaluation of the telehealth making sense of brain tumor psychological support intervention for people with primary brain tumor and their caregivers: A randomized controlled trial." Psycho-oncology **32**(9): 1385-1394.
Padraig, D., H. Sandra, E. Louisa, T. Clare, G. Daisy, F. Alexis, L. Shirley, A. M. Alan and S. Chris (2016). "Cost-effectiveness of telehealth for patients with raised cardiovascular disease risk: evidence from the Healthlines randomised controlled trial." BMJ Open **6**(8): e012352.
Pak, S. S., D. Janela, N. Freitas, F. Costa, R. Moulder, M. Molinos, A. C. Areias, V. Bento, S. P. Cohen, V. Yanamadala, R. B. Souza and F. D. Correia (2023). "Comparing Digital to Conventional Physical Therapy for Chronic Shoulder Pain: Randomized Controlled Trial." Journal of Medical Internet Research **25**: e49236.
París, A. P., H. G. Saleta, M. de la Cruz Crespo Maraver, E. Silvestre, M. G. Freixa, C. P. Torrellas, S. A. Pont, M. F. Nadal, S. A. Garcia, M. V. P. Bartolomé, V. L. Fernández and À. R. Bayés (2011). "Blind randomized controlled study of the efficacy of cognitive training in Parkinson's disease." Movement Disorders **26**(7): 1251-1258.
Pastora-Bernal, J. M., R. Martin-Valero and F. J. Baron-Lopez (2018). "Cost analysis of telerehabilitation after arthroscopic subacromial decompression." Journal of telemedicine and telecare **24**(8): 553-559.
Pastora-Bernal, J. M., R. Martin-Valero, F. J. Baron-Lopez, N. G. Moyano and M. J. Estebanez-Perez (2018). "Telerehabilitation after arthroscopic subacromial decompression is effective and not inferior to standard practice: Preliminary results." Journal of telemedicine and telecare **24**(6): 428-433.
Patel, S., O. Ojo, G. Genc, S. Oravivattanakul, Y. Huo, T. Rasameesoraj, L. Wang, J. Bena, M. Drerup, N. Foldvary-Schaefer, A. Ahmed and H. H. Fernandez (2017). "A Computerized Cognitive behavioral therapy Randomized, Controlled, pilot trial for insomnia in Parkinson Disease (ACCORD-PD)." J Clin Mov Disord **4**: 16.
Paul, L., L. Renfrew, J. Freeman, H. Murray, B. Weller, P. Mattison, A. McConnachie, R. Heggie, O. Wu and E. H. Coulter (2019). "Web-based physiotherapy for people affected by multiple sclerosis: a single blind, randomized controlled feasibility study." Clin Rehabil **33**(3): 473-484.
Phillips, V. L., S. Vesmarovich, R. Hauber, E. Wiggers and A. Egner (2001). "Telehealth: Reaching Out to Newly Injured Spinal Cord Patients." Public Health Reports **116**(1_suppl): 94-102.
Pidgeon, F. M. (2017). "Use of telehealth videoconferencing as a supplement to visiting allied health services." Australian Journal of Rural Health **25**(1): 58-59.
Pignato, M., L. Arbeeva, T. A. Schwartz, L. F. Callahan, J. Cooke, Y. M. Golightly, A. P. Goode, B. C. Heiderscheit, C. Hill, K. M. Huffman, H. H. Severson and K. D. Allen (2018). "Level of participation in physical therapy or an internet-based exercise training program: associations with outcomes for patients with knee osteoarthritis." BMC Musculoskelet Disord **19**(1): 238.
Piotrowicz, E., R. Baranowski, M. Bilinska, M. Stepnowska, M. Piotrowska, A. Wójcik, J. Korewicki, L. Chojnowska, L. A. Malek, M. Klopotowski, W. Piotrowski and R. Piotrowicz (2010). "A new model of home-based telemonitored cardiac rehabilitation in patients with heart failure: effectiveness, quality of life, and adherence." European Journal of Heart Failure **12**(2): 164-171.
Piotrowicz, E., A. Mierzyńska, I. Jaworska, G. Opolski, M. Banach, W. Zaręba, I. Kowalik, M. Pencina, P. Orzechowski, D. Szalewska, S. Pluta, R. Glowczynska, Z. Kalarus, R. Irzmanski and R. Piotrowicz (2022). "Relationship between physical capacity and depression in heart failure patients undergoing hybrid comprehensive telerehabilitation vs. usual care: subanalysis from the TELEREH-HF Randomized Clinical Trial." European Journal of Cardiovascular Nursing **21**(6): 568-577.
Piron, L., A. Turolla, M. Agostini, C. Zucconi, F. Cortese, M. Zampolini, M. Zannini, M. Dam, L. Ventura, M. Battauz and P. Tonin (2009). "Exercises for paretic upper limb after stroke: a combined virtual-reality and telemedicine approach." J Rehabil Med **41**(12): 1016-1102.

Poon, P., E. Hui, D. Dai, T. Kwok and J. Woo (2005). "Cognitive intervention for community-dwelling older persons with memory problems: telemedicine versus face-to-face treatment." International Journal of Geriatric Psychiatry **20**(3): 285-286.
Poulin, V., N. Korner-Bitensky, L. Bherer, M. Lussier and D. R. Dawson (2017). "Comparison of two cognitive interventions for adults experiencing executive dysfunction post-stroke: a pilot study." Disability and rehabilitation **39**(1): 1-13.
Price, M. and D. F. Gros (2014). "Examination of prior experience with telehealth and comfort with telehealth technology as a moderator of treatment response for PTSD and depression in veterans." Int J Psychiatry Med **48**(1): 57-67.
Pyne, J. M., J. C. Fortney, S. P. Tripathi, M. L. Maciejewski, M. J. Edlund and D. K. Williams (2010). "Cost-effectiveness analysis of a rural telemedicine collaborative care intervention for depression." Arch Gen Psychiatry **67**(8): 812-821.
Raina, K. D., J. Q. Morse, D. Chisholm, E. M. Whyte and L. Terhorst (2022). "An Internet-Based Self-Management Intervention to Reduce Fatigue Among People With Traumatic Brain Injury: A Pilot Randomized Controlled Trial." American Journal of Occupational Therapy **76**(4): 1-8.
Rakhshan, M., Z. Khoshnood, L. Ansari and A. Aslani (2022). "Body Image and Adjustment among Patients with Heart Rhythm Management Devices following Cardiac Rehabilitation Program: A Randomized, Controlled Clinical Trial." Clin Med Res **20**(1): 1-8.
Ransom, D., T. Heckman, T. Anderson, J. P. Garske, K. A. Holroyd and T. B. Basta (2008). "Telephone-delivered, interpersonal psychotherapy for HIV-infected rural persons with depression: a pilot trial." Psychiatric services **59 8**: 871-877.
Redfern, J., T. Briffa, E. Ellis and S. B. Freedman (2008). "Patient-Centered Modular Secondary Prevention Following Acute Coronary Syndrome: A RANDOMIZED CONTROLLED TRIAL." Journal of Cardiopulmonary Rehabilitation and Prevention **28**(2).
Redfern, J., T. Briffa, E. Ellis and S. B. Freedman (2009). "Choice of secondary prevention improves risk factors after acute coronary syndrome: 1-year follow-up of the CHOICE (Choice of Health Options In prevention of Cardiovascular Events) randomised controlled trial." Heart **95**(6): 468-475.
Redzuan, N. S., J. P. Engkasan, M. Mazlan and S. J. Freddy Abdullah (2012). "Effectiveness of a Video-Based Therapy Program at Home After Acute Stroke: A Randomized Controlled Trial." Archives of Physical Medicine and Rehabilitation **93**(12): 2177-2183.
Reuter, I., S. Mehnert, G. Sammer, M. Oechsner and M. Engelhardt (2012). "Efficacy of a multimodal cognitive rehabilitation including psychomotor and endurance training in Parkinson's disease." J Aging Res **2012**: 235765.
Riegler, L. J., J. Neils-Strunjas, S. Boyce, S. L. Wade and P. M. Scheifele (2013). "Cognitive intervention results in web-based videophone treatment adherence and improved cognitive scores." Med Sci Monit **19**: 269-275.
Russell, T., P. Buttrum, R. Wootton and G. Jull (2011). "Internet-Based Outpatient Telerehabilitation for Patients Following Total Knee Arthroplasty." The Journal of bone and joint surgery. American volume **93**: 113-120.
Russell, T. G., P. Buttrum, R. Wootton and G. A. Jull (2003). "Low-bandwidth telerehabilitation for patients who have undergone total knee replacement: Preliminary results." Journal of Telemedicine and Telecare **9**(2_suppl): 44-47.
Salazar, A. M., D. L. Warden, K. Schwab, J. Spector, S. Braverman, J. Walter, R. Cole, M. M. Rosner, E. M. Martin, J. Ecklund and R. G. Ellenbogen (2000). "Cognitive rehabilitation for traumatic brain injury: A randomized trial. Defense and Veterans Head Injury Program (DVHIP) Study Group." Jama **283**(23): 3075-3081.
Salvetti, X. M., J. A. Oliveira, D. M. Servantes and A. A. Vincenzo de Paola (2008). "How much do the benefits cost? Effects of a home-based training programme on cardiovascular fitness, quality of life, programme cost and adherence for patients with coronary disease." Clin Rehabil **22**(10-11): 987-996.

Sankaran, S., P. Dendale and K. Coninx (2019). "Evaluating the Impact of the HeartHab App on Motivation, Physical Activity, Quality of Life, and Risk Factors of Coronary Artery Disease Patients: Multidisciplinary Crossover Study." JMIR Mhealth Uhealth **7**(4): e10874.
Sari, F., D. Oskay and A. Tufan (2022). "THE EFFECTS of TELEREHABILITATION BASED EXERCISE PROGRAM in PATIENTS with SYSTEMIC SCLEROSIS." Annals of the Rheumatic Diseases **81**(Supplement 1): 1851-1852.
Scheenen, M., A. Visser-Keizer, H. Van Der Horn, M. De Koning, P. Van De Sande, M. Van Kessel, J. Van Der Naalt and J. Spikman (2017). "Cognitive behavioural intervention compared to telephone counselling early after mild traumatic brain injury: A randomized trial." Brain Injury **31**(6-7): 761.
Scherr, D., P. Kastner, A. Kollmann, A. Hallas, J. Auer, H. Krappinger, H. Schuchlenz, G. Stark, W. Grander, G. Jakl, G. Schreier and F. M. Fruhwald (2009). "Effect of home-based telemonitoring using mobile phone technology on the outcome of heart failure patients after an episode of acute decompensation: randomized controlled trial." J Med Internet Res **11**(3): e34.
Seto, E., K. J. Leonard, J. A. Cafazzo, J. Barnsley, C. Masino and H. J. Ross (2012). "Mobile phone-based telemonitoring for heart failure management: a randomized controlled trial." J Med Internet Res **14**(1): e31.
Severe, J., P. N. Pfeiffer, K. Palm-Cruz, T. Hoeft, R. Sripada, M. Hawrilenko, S. Chen and J. Fortney (2022). "Clinical Predictors of Engagement in Teleintegrated Care and Telereferral Care for Complex Psychiatric Disorders in Primary Care: a Randomized Trial." JGIM: Journal of General Internal Medicine **37**(13): 3361-3367.
Shahidi, S., F. Zargar, H. Aghaee Khajelangi and M. J. Tarrahi (2023). "The Effect of Internet-delivered Mindfulness Stress Reduction Combined with Acceptance and Commitment Therapy on Health Anxiety and Quality of Life of Caregiver of Patients Infected by COVID-19: A Randomized Clinical Trial." Int J Community Based Nurs Midwifery **11**(2): 110-121.
Shanshan, W., D. Sze Ki Cheung, A. Yee Man Leung and P. M. Davidson (2021). "Bibliotherapy for improving caregiving appraisal of informal caregivers of people with dementia: A pilot randomized controlled trial." Research in Nursing & Health **44**(4): 692-703.
Shetty, S., D. K. Ku and P. Chippala (2022). "Supervised Physical Therapy Versus Video-Assisted Technique for Chronic Mechanical Low Back Pain: A Comparative Study." Journal of Health & Allied Sciences NU **12**(3): 277-279.
Smith, G. C., N. Egbert, M. Dellman-Jenkins, K. Nanna and P. A. Palmieri (2012). "Reducing depression in stroke survivors and their informal caregivers: a randomized clinical trial of a Web-based intervention." Rehabil Psychol **57**(3): 196-206.
Smith, J., S. G. Faux, T. Gardner, M. J. Hobbs, M. A. James, A. E. Joubert, N. Kladnitski, J. M. Newby, R. Schultz, C. T. Shiner and G. Andrews (2019). "Reboot Online: A Randomized Controlled Trial Comparing an Online Multidisciplinary Pain Management Program with Usual Care for Chronic Pain." Pain Medicine (United States) **20**(12): 2385-2396.
Smith, K. M., H. M. Arthur, R. S. McKelvie and J. Kodis (2004). "Differences in sustainability of exercise and health-related quality of life outcomes following home or hospital-based cardiac rehabilitation." European Journal of Cardiovascular Prevention & Rehabilitation **11**(4): 313-319.
Smith, K. M., R. S. McKelvie, K. E. Thorpe and H. M. Arthur (2011). "Six-year follow-up of a randomised controlled trial examining hospital versus home-based exercise training after coronary artery bypass graft surgery." Heart **97**(14): 1169-1174.
Stefan, S. D., D (2013). "Face-to-face counseling versus high definition holographic projection system. Efficacy and therapeutic alliance. A brief research report." Journal of Cognitive and Behavioral Psychotherapies **13**(2): 299-307.
Stein, M. D., D. S. Herman, D. Bishop, B. J. Anderson, E. Trisvan, R. Lopez, T. Flanigan and I. Miller (2007). "A telephone-based intervention for depression in HIV patients: negative results from a randomized clinical trial." AIDS Behav **11**(1): 15-23.

Strachan, M., D. F. Gros, K. J. Ruggiero, C. W. Lejuez and R. Acierno (2012). "An Integrated Approach to Delivering Exposure-Based Treatment for Symptoms of PTSD and Depression in OIF/OEF Veterans: Preliminary Findings." Behavior Therapy **43**(3): 560-569.
Streater, A., A. Spector, Z. Hoare, E. Aguirre, I. Russell and M. Orrell (2017). "Staff training and outreach support for Cognitive Stimulation Therapy and its implementation in practice: a cluster randomised trial." International Journal of Geriatric Psychiatry **32**(12): e64-e71.
Stubbings, D., C. Rees, L. Roberts and R. Kane (2013). "Comparing In-Person to Videoconference-Based Cognitive Behavioral Therapy for Mood and Anxiety Disorders: Randomized Controlled Trial." Journal of medical Internet research **15**: e258.
Stuifbergen, A. K., H. Becker, F. Perez, J. Morison, V. Kullberg and A. Todd (2012). "A randomized controlled trial of a cognitive rehabilitation intervention for persons with multiple sclerosis." Clin Rehabil **26**(10): 882-893.
Tan, G., I. Teo, D. Srivastava, D. Smith, S. L. Smith, W. Williams and M. P. Jensen (2013). "Improving access to care for women veterans suffering from chronic pain and depression associated with trauma." Pain Med **14**(7): 1010-1020.
Taylor, D. J., A. L. Peterson, K. E. Pruiksma, S. Young-McCaughan, K. Nicholson and J. Mintz (2017). "Internet and In-Person Cognitive Behavioral Therapy for Insomnia in Military Personnel: A Randomized Clinical Trial." Sleep **40**(6).
Taylor, R. S., A. Watt, H. M. Dalal, P. H. Evans, J. L. Campbell, K. L. Read, A. J. Mourant, J. Wingham, D. R. Thompson and D. J. Pereira Gray (2007). "Home-based cardiac rehabilitation versus hospital-based rehabilitation: a cost effectiveness analysis." Int J Cardiol **119**(2): 196-201.
Torpil, B. and Ö. Kaya (2022). "The Effectiveness of Client-Centered Intervention With Telerehabilitation Method After Total Knee Arthroplasty." OTJR: Occupation, Participation & Health **42**(1): 40-49.
Torrisi, M., G. Maresca, M. C. De Cola, A. Cannavò, F. Sciarrone, G. Silvestri, A. Bramanti, R. De Luca and R. S. Calabrò (2019). "Using telerehabilitation to improve cognitive function in post-stroke survivors: is this the time for the continuity of care?" Int J Rehabil Res **42**(4): 344-351.
Tousignant, M., P. Boissy, H. Corriveau and H. Moffet (2006). "In home telerehabilitation for older adults after discharge from an acute hospital or rehabilitation unit: A proof-of-concept study and costs estimation." Disability and Rehabilitation: Assistive Technology **1**(4): 209-216.
Tousignant, M., H. Moffet, P. Boissy, H. Corriveau, F. Cabana and F. Marquis (2011). "A randomized controlled trial of home telerehabilitation for post-knee arthroplasty." J Telemed Telecare **17**(4): 195-198.
Tousignant, M., H. Moffet, S. Nadeau, C. Mérette, P. Boissy, H. Corriveau, F. Marquis, F. Cabana, P. Ranger, L. Belzile É and R. Dimentberg (2015). "Cost analysis of in-home telerehabilitation for post-knee arthroplasty." J Med Internet Res **17**(3): e83.
Tuerk, P. W., M. Yoder, K. J. Ruggiero, D. F. Gros and R. Acierno (2010). "A pilot study of prolonged exposure therapy for posttraumatic stress disorder delivered via telehealth technology." J Trauma Stress **23**(1): 116-123.
Türkmen, E., Y. Analay Akbaba and S. Altun (2020). "Effectiveness of video-based rehabilitation program on pain, functionality, and quality of life in the treatment of rotator cuff tears: A randomized controlled trial." Journal of Hand Therapy **33**(3): 288-295.
Turolla, A., G. Rossettini, A. Viceconti, A. Palese and T. Geri (2020). "Musculoskeletal Physical Therapy During the COVID-19 Pandemic: Is Telerehabilitation the Answer?" Phys Ther **100**(8): 1260-1264.
Vale, M. J., M. V. Jelinek, J. D. Best, A. M. Dart, L. E. Grigg, D. L. Hare, B. P. Ho, R. W. Newman and J. J. McNeil (2003). "Coaching patients On Achieving Cardiovascular Health (COACH): a multicenter randomized trial in patients with coronary heart disease." Arch Intern Med **163**(22): 2775-2783.

Vallejo, M. A., J. Ortega, J. Rivera, M. I. Comeche and L. Vallejo-Slocker (2015). "Internet versus face-to-face group cognitive-behavioral therapy for fibromyalgia: A randomized control trial." J Psychiatr Res **68**: 106-113.
van den Berg, M., M. P. Crotty, E. Liu, M. Killington, G. P. Kwakkel and E. van Wegen (2016). "Early Supported Discharge by Caregiver-Mediated Exercises and e-Health Support After Stroke: A Proof-of-Concept Trial." Stroke **47**(7): 1885-1892.
van der Linden, S. D., M. M. Sitskoorn, G. M. Rutten and K. Gehring (2018). "Feasibility of the evidence-based cognitive telerehabilitation program Remind for patients with primary brain tumors." J Neurooncol **137**(3): 523-532.
van Vugt, V. A., M. W. Heymans, J. C. van der Wouden, H. E. van der Horst and O. R. Maarsingh (2020). "Treatment success of internet-based vestibular rehabilitation in general practice: development and internal validation of a prediction model." BMJ open **10**(10): e038649.
Varnfield, M., M. Karunanithi, C. K. Lee, E. Honeyman, D. Arnold, H. Ding, C. Smith and D. L. Walters (2014). "Smartphone-based home care model improved use of cardiac rehabilitation in postmyocardial infarction patients: results from a randomised controlled trial." Heart **100**(22): 1770-1779.
Vasconcellos, L. S., R. S. Silva, T. B. Pacheco, D. A. Nagem, C. O. Sousa and T. S. Ribeiro (2023). "Telerehabilitation-based trunk exercise training for motor symptoms of individuals with Parkinson's disease: A randomized controlled clinical trial." Journal of telemedicine and telecare **29**(9): 698-706.
Vauth, F., Hampel, P., Scibor, M., Handschu, R., Richter, J., & Keidel, M. (2008). "Synchronic telepractise: A new (additional) form of aphasia therapy." Forum Logopädie **22**(4): 12-19.
Vazquez, F. L., L. Lopez, A. J. Torres, P. Otero, V. Blanco, O. Diaz and M. Paramo (2020). "Analysis of the components of a cognitive-behavioral intervention for the prevention of depression administered via conference call to nonprofessional caregivers: A randomized controlled trial." International Journal of Environmental Research and Public Health **17**(6): 2067.
Venter, A., R. Burns, M. Hefford and N. Ehrenberg (2012). "Results of a telehealth-enabled chronic care management service to support people with long-term conditions at home." Journal of Telemedicine and Telecare **18**(3): 172-175.
Villatoro-Luque, F. J., D. Rodriguez-Almagro, A. Aibar-Almazan, S. Fernandez-Carnero, D. Pecos-Martin, A. J. Ibanez-Vera and A. Achalandabaso-Ochoa (2023). "In non-specific low back pain, is an exercise program carried out through telerehabilitation as effective as one carried out in a physiotherapy center? A controlled randomized trial." Musculoskeletal Science and Practice **65**: 102765.
Vitacca, M., L. Bianchi, A. Guerra, C. Fracchia, A. Spanevello, B. Balbi and S. Scalvini (2009). "Tele-assistance in chronic respiratory failure patients: a randomised clinical trial." Eur Respir J **33**(2): 411-418.
Vogel, P. A., G. Launes, E. M. Moen, S. Solem, B. Hansen, Å. T. Håland and J. A. Himle (2012). "Videoconference- and cell phone-based cognitive-behavioral therapy of obsessive-compulsive disorder: A case series." Journal of Anxiety Disorders **26**(1): 158-164.
Vuletic, S., K. R. Bell, S. Jain, N. Bush, N. Temkin, J. R. Fann, K. E. Stanfill, S. Dikmen, J. A. Brockway, F. He, K. Ernstrom, R. Raman, G. Grant, M. B. Stein and G. A. Gahm (2016). "Telephone Problem-Solving Treatment Improves Sleep Quality in Service Members With Combat-Related Mild Traumatic Brain Injury: Results From a Randomized Clinical Trial." J Head Trauma Rehabil **31**(2): 147-157.
Wakasa, M., T. Odashima, A. Saito, M. Kimoto, I. Saito, S. Handa, K. Syukunobe, Y. Kume and K. Okada (2020). "Telerehabilitation with Tablet Computers Replaces Face-to-Face Rehabilitation." Physical & Occupational Therapy in Geriatrics **38**(1): 85-97.
Wang, L., Y. Guo, Y. Liu, X. Yan and R. Ding (2022). "The effects of a mobile phone-based psychological intervention program on stress, anxiety and self-efficacy among undergraduate nursing students during clinical practice: A randomized controlled trial." J Prof Nurs **42**: 219-224.

Wangnamthip, S., I. Tip-apakoon, N. Benjangkhaprasert, N. Bunwatsana, S. Panchoowong and P. Euasobhon (2023). "Comparative Effectiveness of Video and Face-to-Face Sessions to Educate Hospitalized Patients on Cancer Pain Management." Siriraj Medical Journal **75**(4): 306-315.
Whealin, J. M., L. A. Seibert-Hatalsky, J. W. Howell and J. Tsai (2015). "E-mental health preferences of Veterans with and without probable posttraumatic stress disorder." J Rehabil Res Dev **52**(6): 725-738.
Wilkinson, J. R., M. Spindler, S. M. Wood, S. C. Marcus, D. Weintraub, J. F. Morley, M. G. Stineman and J. E. Duda (2016). "High patient satisfaction with telehealth in Parkinson disease: A randomized controlled study." Neurol Clin Pract **6**(3): 241-251.
Wilkinson, O. M., F. Duncan-Skingle, J. A. Pryor and M. E. Hodson (2008). "A feasibility study of home telemedicine for patients with cystic fibrosis awaiting transplantation." J Telemed Telecare **14**(4): 182-185.
Wolf, S. L., K. Sahu, R. C. Bay, S. Buchanan, A. Reiss, S. Linder, A. Rosenfeldt and J. Alberts (2015). "The HAAPI (Home Arm Assistance Progression Initiative) Trial: A Novel Robotics Delivery Approach in Stroke Rehabilitation." Neurorehabilitation and Neural Repair **29**(10): 958-968.
Wu, G., L. Keyes, P. Callas, X. Ren and B. Bookchin (2010). "Comparison of telecommunication, community, and home-based Tai Chi exercise programs on compliance and effectiveness in elders at risk for falls." Arch Phys Med Rehabil **91**(6): 849-856.
Wu, S.-K., Y.-W. Lin, C.-L. Chen and S.-W. Tsai (2006). "Cardiac Rehabilitation vs. Home Exercise After Coronary Artery Bypass Graft Surgery: A Comparison of Heart Rate Recovery." American Journal of Physical Medicine & Rehabilitation **85**: 711-717.
Yavas, I., T. Kahraman, O. Sagici, A. T. Ozdogar, P. Yigit, C. Baba and S. Ozakbas (2023). "Feasibility of Telerehabilitation-Based Pelvic Floor Muscle Training for Urinary Incontinence in People With Multiple Sclerosis: A Randomized, Controlled, Assessor-Blinded Study." Journal of Neurologic Physical Therapy **47**(4): 217-226.
Yilmaz Yelvar, G. D., Y. Çırak, M. Dalkılınç, Y. Parlak Demir, Z. Guner and A. Boydak (2017). "Is physiotherapy integrated virtual walking effective on pain, function, and kinesiophobia in patients with non-specific low-back pain? Randomised controlled trial." European Spine Journal **26**(2): 538-545.
Ying, Y., Y. Ji, F. Kong, M. Wang, Q. Chen, L. Wang, Y. Hou, L. Yu, L. Zhu, P. Miao, J. Zhou, L. Zhang, Y. Yang, G. Wang, R. Chen, D. Liu, W. Huang, Y. Lv, Z. Lou and L. Ruan (2023). "Efficacy of an internet-based cognitive behavioral therapy for subthreshold depression among Chinese adults: a randomized controlled trial." Psychological medicine **53**(9): 3932-3942.
Young, P. M., A. Y. Chen, A. R. Ford, M. Y. Cheng, C. J. Lane and A. W. Armstrong (2023). "Effects of online care on functional and psychological outcomes in patients with psoriasis: A randomized controlled trial." Journal of the American Academy of Dermatology **88**(2): 364-370.
Yuen, E. K., D. F. Gros, M. Price, S. Zeigler, P. W. Tuerk, E. B. Foa and R. Acierno (2015). "Randomized Controlled Trial of Home-Based Telehealth Versus In-Person Prolonged Exposure for Combat-Related PTSD in Veterans: Preliminary Results." J Clin Psychol **71**(6): 500-512.
Ziemba, S. J., N. S. Bradley, L. A. Landry, C. H. Roth, L. S. Porter and R. N. Cuyler (2014). "Posttraumatic stress disorder treatment for Operation Enduring Freedom/Operation Iraqi Freedom combat veterans through a civilian community-based telemedicine network." Telemed J E Health **20**(5): 446-450.
Zwisler, A. D., A. M. Soja, S. Rasmussen, M. Frederiksen, S. Abedini, J. Appel, H. Rasmussen, C. Gluud, L. Iversen, B. Sigurd, M. Madsen and J. Fischer-Hansen (2008). "Hospital-based comprehensive cardiac rehabilitation versus usual care among patients with congestive heart failure, ischemic heart disease, or high risk of ischemic heart disease: 12-month results of a randomized clinical trial." Am Heart J **155**(6): 1106-1113.
